# Force-responsive biomaterials drive tissue repair by harnessing endogenous growth factors

**DOI:** 10.1038/s41563-026-02682-8

**Published:** 2026-07-27

**Authors:** Magdalene Y. Ho, Nuria Oliva, Christopher Basu, Marcos R. Rodriguez, Jose Antonio Duran-Mota, Divya M. Gollapalli, Victor G. Szwarcberg, Mo Akhavani, Kyle P. Quinn, Benjamin D. Almquist

**Affiliations:** 1 Department of Bioengineering, Imperial College London, London, UK; 2 Department of Bioengineering, Institut Químic de Sarria, Universitat Ramon Llull, Barcelona, Spain; 3 School of Veterinary Medicine, University of Surrey, Guildford, UK; 4 Department of Biomedical Engineering, University of Arkansas, Fayetteville, AR USA; 5 Department of Biomedical Engineering, Tufts University, Medford, MA USA; 6 Department of Plastic and Reconstructive Surgery, Royal Free Hospital, London, UK

**Keywords:** Biomedical materials, Biomedical materials, Drug delivery, Drug delivery, Biomedical engineering

## Abstract

Materials enabling the cell-responsive delivery of endogenous biologics, such as growth factors, have the potential to modulate wound repair cost-effectively and safely. Unlike passive drug delivery strategies that require supraphysiological doses of recombinant protein or stimuli-responsive systems that rely on external triggers, we demonstrate a strategy that harnesses cellular traction forces as an intrinsic delivery trigger. Traction-force-activated payloads are bioinspired aptamer constructs attached to biomaterial scaffolds that selectively harvest, concentrate and reactivate multiple endogenous growth factors from cells, injury sites and blood lysate in vivo (rat femur and mouse skin) and ex vivo (human skin), at doses orders of magnitude lower than current clinical standards. Unmodified oligonucleotide aptamers retain functionality in enzyme-rich wound environments, substantially expanding the translational potential of nucleic-acid-based therapeutics. The ability to harvest and redeliver endogenous growth factors without exogenous triggers, recombinant proteins or cold-chain logistics via mechanoresponsive biomaterials opens possibilities for accessible, cost-effective combinatorial biologic therapies.

## Abstract (web summary)

Aptamer constructs attached to biomaterial scaffolds promote tissue repair by the harvesting and cell-activated delivery of endogenous growth factors from cells and injury sites, and are able to modulate cell responses at physiologically relevant doses.

## Main

Repairing damaged tissues is critical for survival and has been a central focus of medical science for thousands of years^1^. Typically, the body orchestrates an efficient sequence of events involving inflammatory cytokines, growth factors and multiple cell types to transition a wound from a damaged, inflamed state to a proliferative, remodelled and healed state^2^. Unfortunately, millions of individuals each year suffer from traumatic acute and chronic wounds that are unable to heal on their own, such as large burn wounds, non-union fractures and diabetic foot ulcers. These difficult wounds require extensive clinical interventions; are extremely painful; reduce a patient’s quality of life; and increase the risk of infection, mortality and/or permanent disability^3–7^. Given the substantial burden these difficult wounds place on patients, families, healthcare systems and economies^8,9^, there is an urgent need for innovative, cost-effective and accessible solutions to promote healing.

One long-standing strategy of interest has been to supplement difficult wounds with growth factors to promote tissue repair. However, its success has been limited by untargeted and inefficient delivery methods. For example, clinically approved strategies rely on passive diffusion via topical gels (Regranex, PDGF-BB), sprays (Fiblast, FGF-2), local injections (Heberprot-P, EGF) or soaked collagen sponges (INFUSE, BMP-2). In these delivery methods, where interactions between therapeutics and the extracellular matrix are limited, proteins are highly unstable, with an in vivo half-life of only a few minutes^10–12^. Thus, supraphysiological amounts of growth factors must be administered over long periods to achieve clinical efficacy, increasing the risk of side effects such as uncontrolled tumour growth or ectopic bone formation^13–17^.

To address these limitations, targeted delivery using stimuli-responsive materials has been explored, using triggers such as light, magnetic fields, pH, hydrolysis and enzymes^18,19^. Although these methods offer enhanced control over delivery mechanisms, they often face challenges such as limited access to specific anatomical locations, difficulties with multiplexed therapeutic delivery and reliance on expensive, low-yield recombinant growth factors. These limitations create regulatory and logistical challenges, especially when using multiple growth factors simultaneously. However, our bodies are optimized to integrate multiple coordinating signals to orchestrate complex, tissue-level functions required for wound healing (for example, angiogenesis, granulation tissue formation and epithelialization)^20–23^. Hence, an alternative approach is to use endogenous growth factors, typically through platelet lysate (PL) in its entirety or as a concentrated gel-like structure called platelet-rich plasma^24–26^. Although this enables a translationally feasible and cost-effective delivery of multiple growth factors, existing platelet lysate methods lack selectivity and still rely on the passive, untargeted release of growth factors. Combining the advantages of both research streams—the selectivity of stimuli-responsive systems and the endogenous sourcing of platelet-rich plasma approaches—requires a fundamentally different design principle: one in which the biological process itself provides both growth factor source and delivery trigger.

**Fig. 1:**
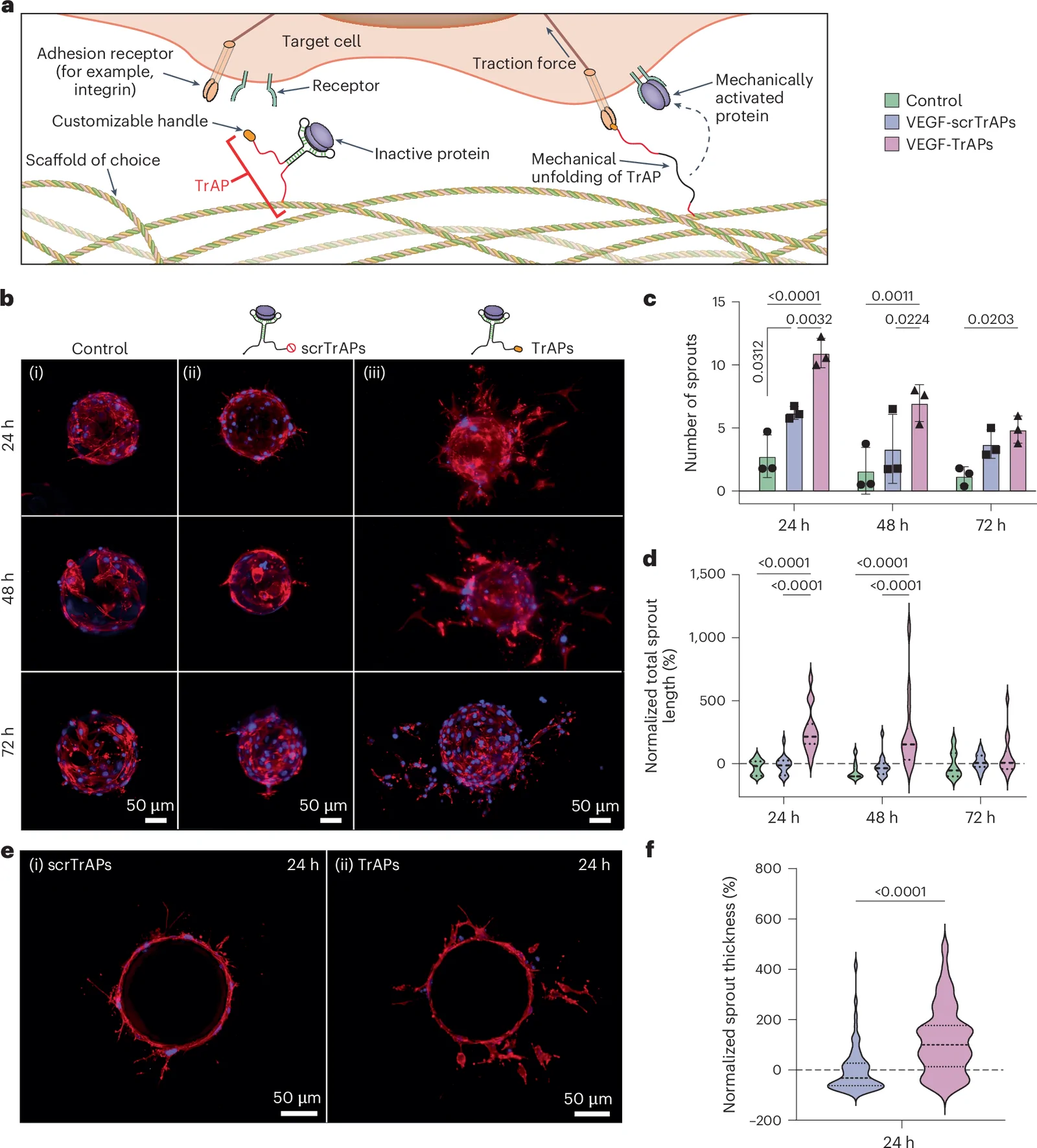
TrAPs release VEGF-A_121_ and promote endothelial sprouting in vitro. **a**, Illustration of cellular-traction-force-mediated growth factor release via the TrAP platform. **b**, Fluorescence images of HMEC-1 endothelial sprouting assays for control, VEGF-scrTrAPs and VEGF-TrAPs groups at 24, 48 and 72 h. Nuclei are stained with DAPI (blue) and actin filaments with phalloidin (red). VEGF-scrTrAPs contain the same VEGF-A binding aptamer sequence as VEGF-TrAPs but incorporate a scrambled (scr) RDG peptide in place of the RGD cell-adhesive peptide ‘handle’. **c**, Quantification of the number of sprouts per bead at 24, 48 and 72 h. **d**, Quantification of the normalized total sprout length per bead at 24, 48 and 72 h. The total sprout length per bead was normalized to the VEGF-scrTrAPs group within each independent experiment. **e**, Cross-sectional view of HMEC-1 sprouts at 24 h reconstructed from confocal *z*–stacks. **f**, Quantification of normalized sprout thickness at 24 h. The sprout thickness was normalized to the mean sprout thickness of the VEGF-scrTrAPs group within each independent experiment set. For **c**, *n* = 3 independent experiments; each data point represents the mean number of sprouts per bead within an experiment. Data are presented as mean ± standard deviation. For **d**, *n* = 15 beads (5 beads analysed per experiment across 3 independent experiments). Statistical analysis for **c** and **d** was performed using two-way analysis of variance (ANOVA) followed by Tukey’s post hoc test. For **f**, *n* = 62 sprouts (VEGF-scrTrAPs) and *n* = 85 sprouts (VEGF-TrAPs), measured from 14 beads across 3 independent experiments. Statistical analysis was performed using a two-tailed unpaired *t*-test. Data are presented as violin plots; dashed lines indicate the median and dotted lines indicate the quartiles (**d** and **f**). Source data

Our approach realizes this principle through bioinspired aptamers that release growth factors in response to cellular traction forces (Fig. 1a)^27^. These traction-force-activated payloads (TrAPs) consist of inhibitory aptamers that bind and inhibit specific growth factors until they are activated and released by cells. With one end anchored to a material substrate and the other conjugated to a cell-adhesive peptide ‘handle’ (for example, RGD), cells can apply mechanical traction force to the ‘handle’, causing TrAPs to unfold, releasing and activating the growth factor. This traction-force-mediated release can also be tailored to target specific cell types^27^. Following our initial demonstration that cellular traction forces are a viable trigger for controlled release, cellular forces have been expanded for use with mesoporous silica nanoparticles^28^ and DNA nanostructures^29^. However, most experiments so far remain limited to in vitro proof-of-concept studies, with one example demonstrating force-mediated drug release from nanoparticles for priming T cells^28^. This leaves an open and important question on whether cellular traction forces can serve as a reliable delivery trigger in the complex, enzyme-rich and mechanically dynamic environment of a repairing tissue, one that is particularly critical given that wound environments present simultaneous challenges of nuclease activity, competing matrix proteins and evolving mechanical properties that can each compromise force-mediated release.

To address this question, here we demonstrate that the cellular traction forces generated during migration and adhesion can serve as an intrinsic mechanical trigger in vivo. Critically, we show that in complex, enzyme-rich in vivo environments, this approach allows growth factors to be held in an inhibited state until mechanically released, eliminating background signalling from passive diffusion. Additionally, we show that TrAP-enabled biomaterials can selectively harvest multiple endogenous growth factors and redeliver them in vitro (human primary cells), in vivo (rat femur and mice skin) and ex vivo (human skin) to modulate angiogenesis and tissue repair in both bone and skin, removing the need for manufactured recombinant proteins.

## Cellular-traction-force-activated vascular endothelial growth factor A modulates angiogenesis in vitro and in vivo

Vascularization is a critical aspect of tissue repair, yet is one of the most challenging to address when lacking^30–33^. Hence, our first objective was to explore whether TrAPs can locally release vascular endothelial growth factor A (VEGF-A), a known pro-angiogenic protein, via cellular traction forces to facilitate vascularization and angiogenic sprouting. To test this, we used preliminary vasculogenesis tube formation assays (Supplementary Figs. 1–3) and an optimized bead sprouting assay (Fig. 1b–f and Supplementary Figs. 4–6). By 24 h, VEGF-TrAPs preloaded with recombinant VEGF-A_121_ promoted significantly more human microvascular endothelial cells-1 (HMEC-1) sprouts (Fig. 1b,c) with increased sprout lengths and thicknesses compared with the preloaded VEGF-scrTrAPs (that is, TrAPs with non-cell-adhesive RDG peptide) and cysteine control groups (Fig. 1b–f). The total sprout length per bead in the preloaded VEGF-TrAPs group was, on average, 200% longer than the preloaded VEGF-scrTrAPs and cysteine control groups (Fig. 1d), and sprouts were approximately 100% thicker than those in the preloaded VEGF-scrTrAPs group (Fig. 1e,f).

VEGF-A stimulates endothelial cells to undergo an intricate, coordinated mechanism in which the selected tip cells adopt a migratory phenotype, extending filopodia-like protrusions towards VEGF-A gradients. By contrast, adjacent stalk cells follow and proliferate behind, supporting the formation of longer angiogenic sprouts^34^. Higher levels of VEGF-A also promote sprout diameter expansion, partially through endothelial cell proliferation^34–36^. Hence, the increased length and thickness of endothelial sprouts in the bead sprouting assay suggest the successful activation of TrAPs via cellular traction force.

**Fig. 2:**
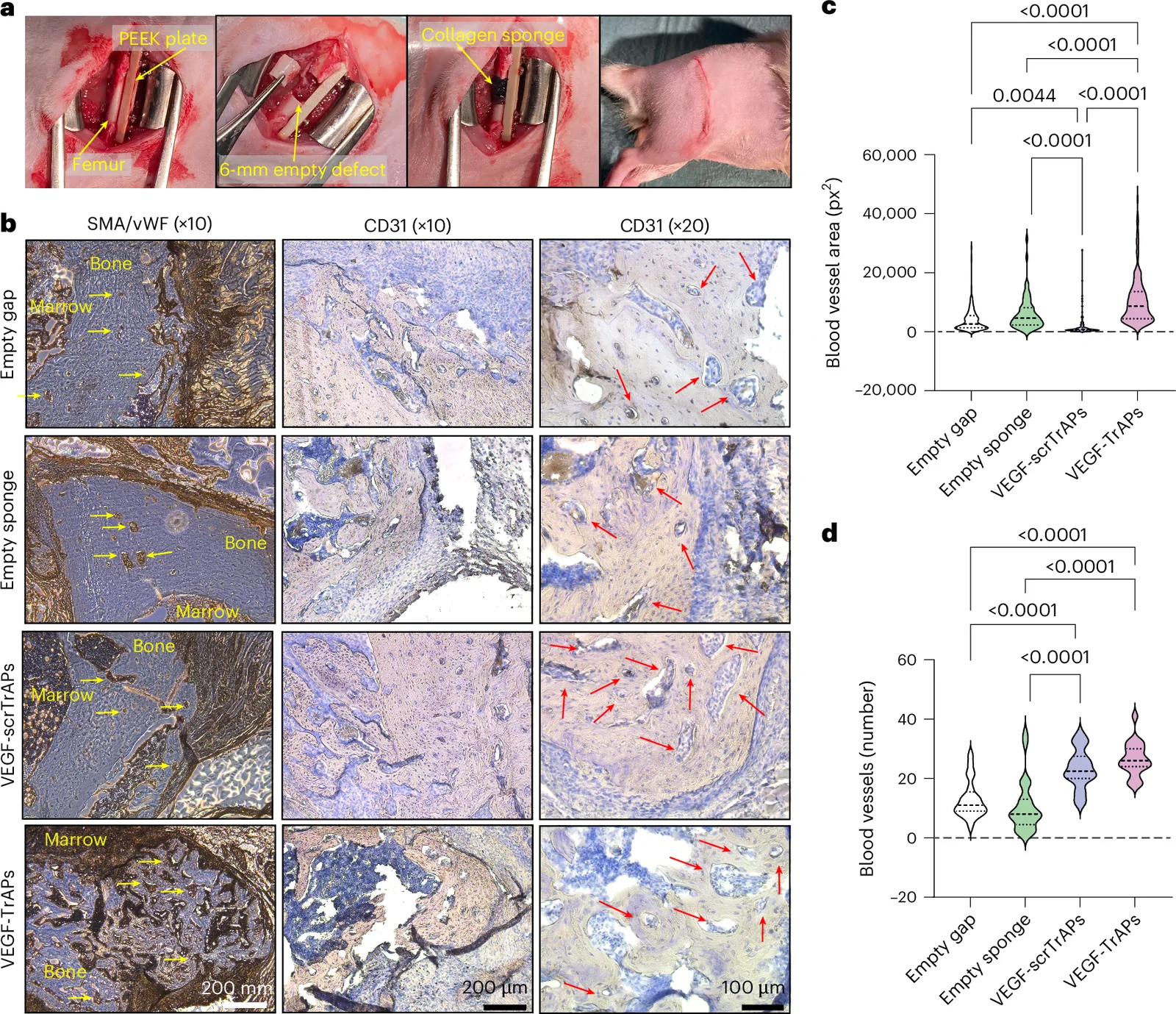
VEGF-TrAP-functionalized collagen sponges promote angiogenesis during bone repair. **a**, Rat femoral defect model. TrAP-functionalized collagen sponges were implanted into a 6 mm femoral defect, stabilized by an internal, screwed-on PEEK plate. VEGF-scrTrAPs and VEGF-TrAPs were preloaded with recombinant VEGF-A_165_. **b**, Cross-sections of the healing femur were stained for alpha smooth muscle actin (α-SMA) and von Willebrand factor (vWF) as an indication for the presence of smooth muscle cells and endothelial cells, respectively. The yellow arrows indicate examples of mature vessels. Cross-sections were also stained for CD31, highly expressed in endothelial cells (red arrow) and used for cross-reference. **c**, Quantification of vessel area determined from α-SMA and vWF staining. **d**, Quantification of CD31-positive vascular structures. For **c**, *n* = 157 vessels (empty gap), *n* = 67 vessels (empty sponge), *n* = 132 vessels (VEGF-scrTrAPs) and *n* = 186 vessels (VEGF-TrAPs). Vessels were randomly selected from histological sections obtained from five rat femurs per group. For **d**, *n* = 21 sections (empty gap), *n* = 24 sections (empty sponge) and *n* = 20 sections (VEGF-scrTrAPs and VEGF-TrAPs). CD31-positive vascular structures were quantified from histological sections obtained from five rat femurs per group, with four sections analysed per femur. Statistical analysis for **c** and **d** was performed using one-way ANOVA, followed by a Tukey–Kramer post hoc test. Data are presented as violin plots; dashed lines indicate the median and dotted lines indicate the quartiles. Source data

Having confirmed that VEGF-TrAPs can actively drive angiogenic sprouting in vitro through cell-activated VEGF-A release, we next evaluated whether TrAPs retain their functional integrity in an actively repairing tissue in vivo. In a 6 mm femoral defect in rats, preloaded VEGF-TrAP-functionalized collagen sponges promoted a greater number of vessels than the empty gap and unfunctionalized sponge groups, and significantly larger vessels than the empty gap, unfunctionalized sponge and preloaded scrTrAP-functionalized sponge groups after 3 weeks (Fig. 2). Interestingly, preloaded VEGF-scrTrAPs yielded vessels that were significantly smaller than those in the control sponges (Fig. 2c), despite being loaded with the same concentration of recombinant VEGF-A as the TrAPs group.

**Extended Data Fig. 1:**
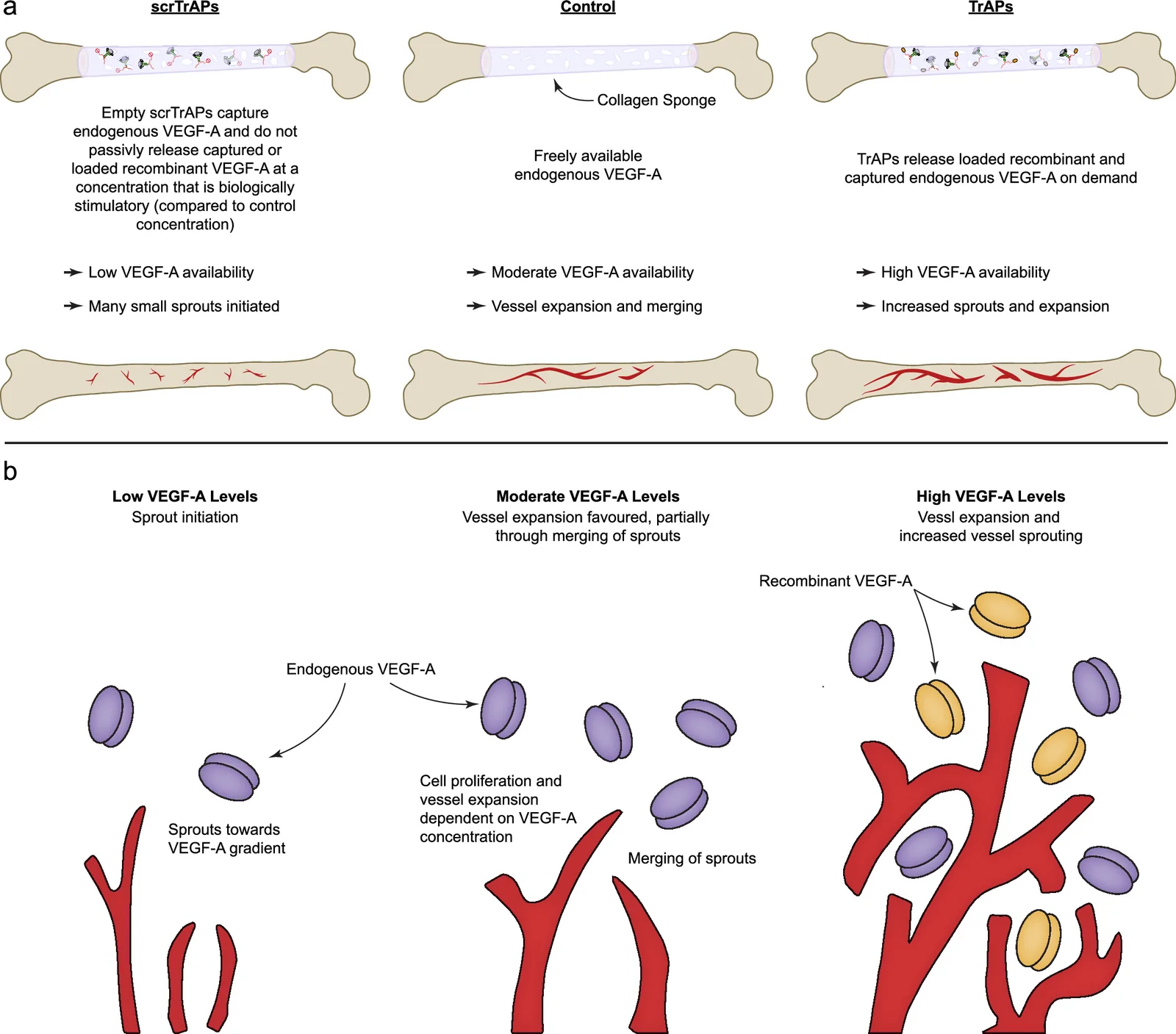
Working hypothesis for how TrAPs modulate VEGF-A concentration in vivo to influence angiogenesis during bone repair. **a**, More scrTrAPs were attached to the collagen scaffolds than recombinant growth factor added, therefore empty scrTrAPs may capture endogenous VEGF-A from the system, reducing the amount locally available to facilitate angiogenesis. With no scrTrAPs/TrAPs functionalized onto the control sponge to actively sequester endogenous VEGF, moderate amounts of VEGF in comparison to the scrTrAP’s group may have been available in the system to favor vessel expansion. Finally, TrAPs may release both captured endogenous and loaded exogenous VEGF-A, supporting increased vessel size and branching. **b**, Prior research indicates that increased VEGF-A promotes vessel expansion and sometimes, though not always, branching.

Previous research suggests that the preloaded VEGF-scrTrAPs group might have lower levels of available endogenous VEGF-A within the wound, compared with the control and preloaded VEGF-TrAPs group (Extended Data Fig. 1). Although increasing VEGF-A concentrations above a certain threshold often increases the vessel size^37–39^, it does not always increase the vessel quantity^35,36,38,40,41^. At low levels of VEGF-A, endothelial cells continuously compete to take on the tip cell phenotype, increasing the likelihood of branching^35,40^. At moderate levels of VEGF-A, the increase in vessel diameter is favoured either through the convergence of tip cells or through cell proliferation, where the former might result in fewer but larger vessels^34–36^ (Extended Data Fig. 1). Meanwhile, at high VEGF-A levels, both sprouting and vessel expansion are supported, increasing both vessel quantity and size^35,36,41,42^. Interestingly, this finding suggests that TrAPs can modulate the growth factor availability across a physiologically relevant range: scrTrAPs reduced available VEGF-A to low physiological levels (producing smaller vessels), whereas TrAPs increased it to a high physiological level (producing larger, more numerous vessels).

Since this study used healthy rats, there was probably sufficient endogenously produced VEGF-A at the injury site^43,44^, where hypoxic tissue is present, to promote vascularization. In particular, scrTrAPs and TrAPs were functionalized at a ratio of approximately 50:1 scrTrAPs/TrAPs:VEGF-A_165_, leaving many unloaded scrTrAPs to capture and inactivate endogenous VEGF-A. Therefore, we hypothesize that femur wounds treated with VEGF-scrTrAPs had less available VEGF-A than control wounds, reducing the ability to support vessel expansion (Extended Data Fig. 1). By contrast, VEGF-TrAPs probably also bound endogenous VEGF-A but, on application of cellular traction forces, could release both captured endogenous and loaded recombinant VEGF-A_165_ to support both expansion and branching^34^. Importantly, VEGF-scrTrAPs did not promote larger vessels, unlike the VEGF-TrAPs, further suggesting that the increased vascularization in the VEGF-TrAPs group was due to VEGF-A released from cellular engagement with the RGD ‘handle’, rather than VEGF-A leakage or aptamer degradation.

Expectedly, bone growth was not affected in any group over 12 weeks (Supplementary Fig. 7), as the treatments were pro-angiogenic (VEGF-A) rather than pro-osteogenic (for example, BMP-2). No negative impact on osteogenesis or adverse events were observed in the VEGF-TrAPs group compared with the empty sponges (Supplementary Fig. 8 and Supplementary Table 5). Additionally, no sponges were found at 3 weeks, suggesting that TrAPs induced lasting changes in vascularization that persisted after degradation of the carrier scaffold. This result provides several important mechanistic insights. First, it demonstrates that the scrTrAPs retained their growth factor-binding function throughout the study period, resisting degradation in the enzyme-rich in vivo wound environment long enough to capture and sequester endogenous VEGF-A from the surrounding tissue, thereby reducing the total available VEGF-A below the level present in untreated control wounds. Second, it confirms that scaffold degradation did not liberate bound growth factors at a biologically relevant level. If collagen sponge breakdown had released the bound VEGF-A, scrTrAPs and TrAPs would have produced similar vascular outcomes, since both groups contained the same loaded concentration. The divergent outcomes between these groups provides in vivo evidence that growth factor release is mediated by cellular engagement with the RGD handle of TrAPs, rather than by passive leakage, aptamer degradation or scaffold breakdown. This highlights a unique feature of the TrAP system: the use of high-affinity inhibitory aptamers (that is, picomolar to sub-picomolar affinity) means that growth factors remain bound to the aptamers after scaffold degradation and are inhibited rather than freely active.

## Harvesting cell-derived growth factors

**Fig. 3:**
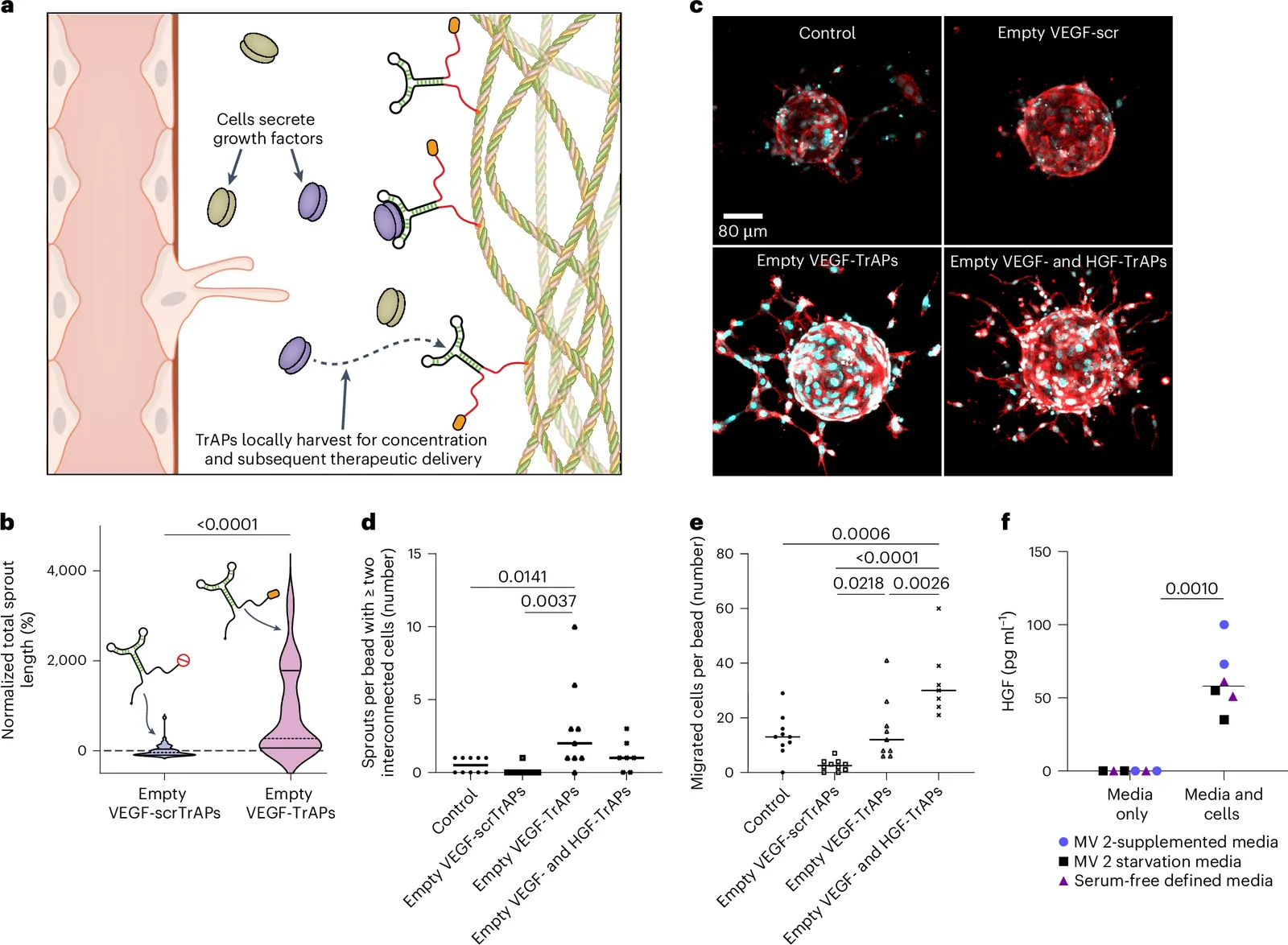
TrAPs capture and redeliver endogenous growth factors produced by cells. **a**, Schematic showing how empty TrAPs capture, concentrate and redeliver secreted endogenous growth factors to the cellular microenvironment to regulate cell behaviour. **b**, Quantification of normalized total endothelial sprout length per bead. The total sprout length per bead was normalized to the mean total sprout length of the empty VEGF-scrTrAPs group within each independent experiment. **c**, Fluorescence images of endothelial sprouting 24 h post-seeding. Nuclei are stained with DAPI (cyan) and actin filaments with phalloidin (red). **d**, Quantification of long endothelial sprouts comprising two or more interconnected cells. **e**, Quantification of HMEC-1 cell migration. **f**, Concentration of HGF in conditioned media at 72 h post-seeding, measured by ELISA. For **b**, *n* = 45 beads (empty VEGF-scrTrAPs) and *n* = 41 beads (empty VEGF-TrAPs), analysed across 4 independent experiments. Statistical analysis was performed using an unpaired two-tailed *t*-test. Data are presented as violin plots; horizontal dotted lines indicate the median and horizontal solid lines indicate the quartiles. For **d** and **e**, *n* = 10 beads (control and empty VEGF-scrTrAPs), *n* = 9 (empty VEGF-TrAPs) and *n* = 7 beads (empty VEGF + HGF-TrAPs). Statistical analysis was performed using one-way ANOVA, followed by a Tukey–Kramer post hoc test. For **f**, *n* = 6 measurements per group, comprising two wells from each of three media conditions. Statistical analysis was performed using Welch’s two-tailed *t*-test. Data are presented as individual data points; horizontal lines indicate the median (**d**–**f**). Source data

Inspired by the possibility of using TrAPs to harvest and deliver endogenous growth factors, we next investigated whether empty TrAPs, without preloading with exogenous growth factors, could regulate cellular processes by capturing endogenous growth factors produced within the cellular microenvironment (Fig. 3a). HMEC-1, known to secrete VEGF-A^45^, were cultured on collagen gels functionalized with empty VEGF-scrTrAPs or empty VEGF-TrAPs, in basal media with 0.2% fetal bovine serum (FBS) supplementation. Preliminary studies suggested that empty VEGF-TrAPs could support larger vascular networks and increased HMEC-1 sprout lengths, similar to preloaded VEGF-TrAPs (Supplementary Figs. 9 and 10). In repeated angiogenic assays, empty VEGF-TrAPs supported greater total sprout lengths of HMEC-1 per bead compared with empty VEGF-scrTrAPs (Fig. 3b,c), suggesting successful harvesting, concentration to a stimulatory level and subsequent redelivery of VEGF-A.

To further assess the robustness of harvesting and concentrating cell-derived growth factors, hepatocyte growth factor (HGF) TrAPs were synthesized, validated (Supplementary Figs. 11 and 12) and introduced into the angiogenic assay (Fig. 3c–e). HMEC-1 were also confirmed to secrete endogenous HGF in various media conditions (Fig. 3f). Although empty VEGF-TrAPs alone supported a greater number of long endothelial sprouts (defined as two or more connected cells), the combined empty VEGF-TrAPs and empty HGF-TrAPs group enhanced cell migration into the functionalized hydrogel within 24 h of incubation (Fig. 3d,e). Given that VEGF-A promotes vessel length and that HGF induces cell motility, these findings suggest that endogenous VEGF-A and HGF were successfully harvested and locally concentrated to a stimulatory threshold that could influence HMEC-1 cellular response when redelivered. These data support our hypothesis that similar harvesting and sequestration probably occurred during the in vivo femur study. It also highlights exciting opportunities for TrAPs as a value-added technology that selectively enriches and efficiently utilizes endogenously secreted proteins within the cellular microenvironment. This provides substantial advantages and cell-responsive control over the use of weakly binding aptamers and passive release systems.

## TrAPs harvest growth factors from biological fluids

**Fig. 4:**
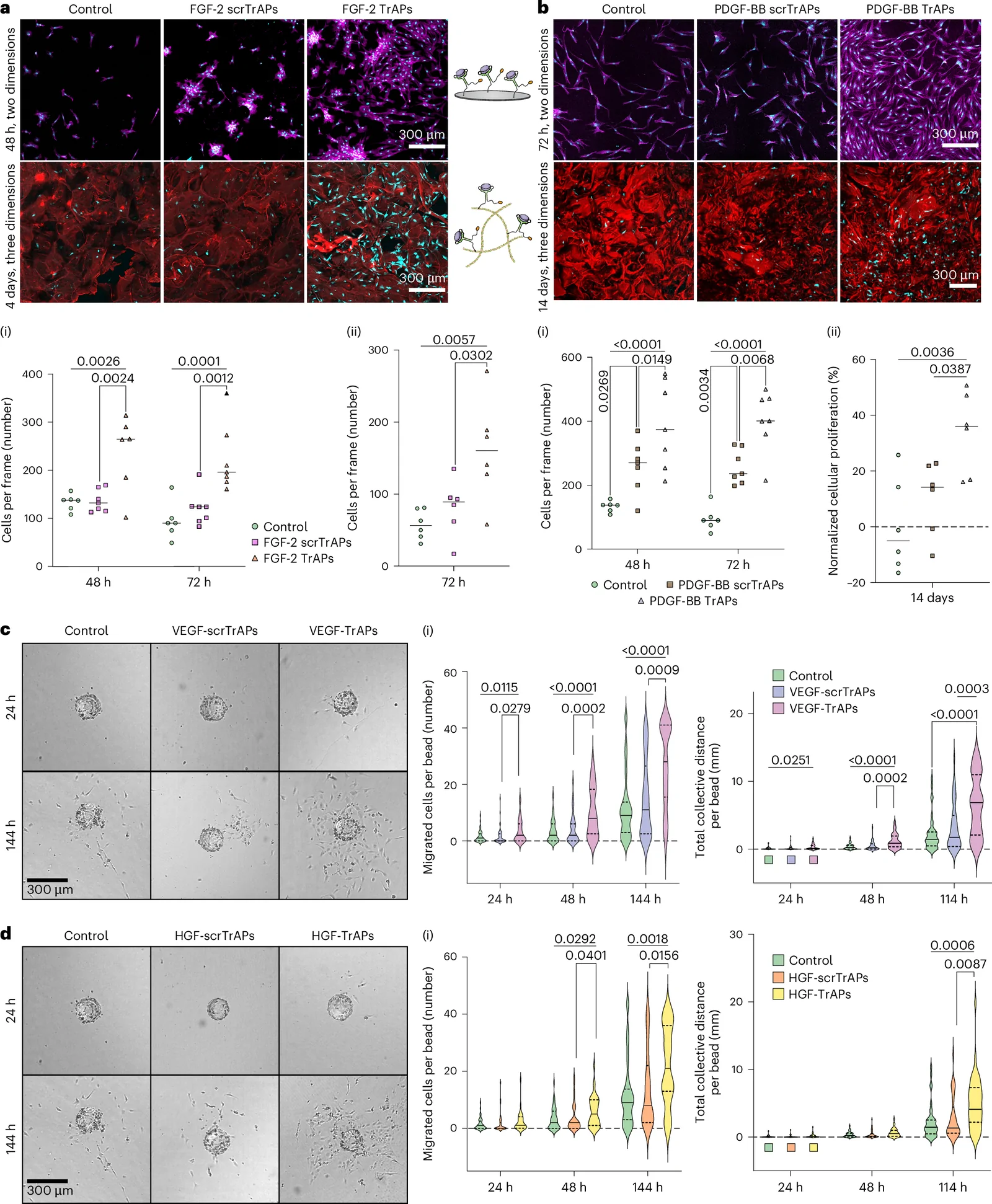
TrAP-functionalized substrates harvest multiple endogenous growth factors from hPL in vitro. **a**,**b**, Fluorescence images and quantification of HDFs cultured on control, scrTrAP-functionalized and TrAP-functionalized coverslips (cyan, DAPI; magenta, phalloidin) and three-dimensional collagen sponges (red, Texas Red; cyan, CellTracker Green CMFDA). **a**(i),**b**(i) Quantification of HDF numbers on coverslips. **a**(ii), Quantification of HDF number on collagen sponges. **b**(ii), Quantification of HDFs metabolic activity within collagen sponges, as a measure of proliferation, using PrestoBlue Cell Viability Reagent. **c**,**d**, Images and quantification of HMEC-1 migration at 24, 48 and 144 h. VEGF-A and HGF experiments conducted in parallel and evaluated against the same control group. **c**(i),**d**(i), Quantification of migrated cells per bead and the total collective distance per bead. For **a**(i), *n* = 7 coverslips (FGF-2-scrTrAPs at 48 and 72 h, and FGF-2-TrAPs at 72 h) and *n* = 6 coverslips (control at 48 and 72 h, and FGF-2-TrAPs at 48 h). For **a**(ii), *n* = 6 sponges. For **b**(i), *n* = 6 (control) and *n* = 7 coverslips (PDGF-BB-scrTrAPs and PDGF-BB-TrAPs). For **b**(ii), *n* = 6 sponges. Statistical analysis was performed using two-way ANOVA, followed by a Tukey–Kramer post hoc test (**a**(i) and **b**(i)) and one-way ANOVA and then by a Tukey’s post hoc test (**a**(ii) and **b**(ii)). Data are presented as individual data points; horizontal lines indicate median (**a**(i)–**b**(ii)). For **c**(i), the number of analysed beads: 24 h, *n* = 45 (control and VEGF-scrTrAPs), *n* = 33 (VEGF-TrAPs); 48 h, *n* = 41 (control and VEGF-scrTrAPs), *n* = 30 beads (VEGF-TrAPs); 144 h, *n* = 38 (control), *n* = 45 (VEGF-scrTrAPs), *n* = 33 (VEGF-TrAPs), from 4 coverslips. For **d**(i), the number of analysed beads: 24 h, *n* = 45 (control), *n* = 42 (HGF-scrTrAPs), *n* = 39 (HGF-TrAPs); 48 h, *n* = 41 (control and HGF-scrTrAPs), *n* = 43 beads (HGF-TrAPs); 144 h, *n* = 38 (control and HGF-scrTrAPs), *n* = 45 (HGF-TrAPs), from 4 coverslips. For **c**(i) and **d**(i), statistical analysis was performed at each time point using one-way ANOVA, followed by a Tukey–Kramer post hoc test. Data are presented as violin plots; horizontal solid lines indicate median and horizontal dashed lines indicate quartiles. Source data

To further evaluate the ability of TrAPs to selectively harvest endogenous growth factors, we explored whether TrAPs could harvest growth factors from biological fluids, expanding the repertoire of endogenous biologics beyond those locally expressed at a patient’s wound site. We began by investigating the harvesting and delivery of complementary growth factors for tissue repair, including platelet-derived growth factor-BB (PDGF-BB), fibroblast growth factor-2 (FGF-2), VEGF-A and HGF, on two-dimensional coverslips (Fig. 4). TrAPs were first confirmed to bind and inhibit their respective recombinant growth factors (Supplementary Figs. 12–14), with growth factor concentrations in human platelet lysate (hPL) quantified via enzyme-linked immunosorbent assay (ELISA; Supplementary Table 6).

We then confirmed that hPL-loaded FGF-2-TrAPs and hPL-loaded PDGF-BB-TrAPs both increased primary human dermal fibroblast (HDF) cell numbers at 48 and 72 h compared with their respective hPL-loaded scrTrAPs and cysteine-functionalized control groups (Fig. 4a(i),b(i)). Consistent with the two-dimensional results, hPL-loaded FGF-2-TrAPs and hPL-loaded PDGF-BB-TrAPs functionalized onto three-dimensional collagen sponges also supported increased HDF cell numbers compared with their respective hPL-loaded scrTrAPs and cysteine-functionalized control groups (Fig. 4a(ii),b(ii)). To account for potential donor-bias observations, these experiments were repeated with HDFs isolated from additional donors (Supplementary Figs. 15 and 16), which demonstrated a similar trend. Thus, these in vitro findings suggest the successful harvesting of growth factors from hPL by their respective TrAPs, and the subsequent successful activation of the growth factors by HDF, to promote proliferation and/or cell survival, a characteristic effect of both FGF-2 and PDGF-BB^46,47^.

To assess the efficacy of harvesting and delivering VEGF-A and HGF from hPL, we used a bead migration assay (Fig. 4c,d). Coverslips functionalized with hPL-loaded VEGF-TrAPs facilitated the increased migration of HMEC-1 cells away from the carrier bead after 24 to 144 h, compared with hPL-loaded scrTrAPs and cysteine-functionalized control coverslips at each time point (Fig. 4c(i)). This enhanced migration aligns with the anticipated response of endothelial cells to VEGF-A on two-dimensional surfaces^48^, indicative of the successful harvesting and activation of VEGF-A from hPL. Similarly, HGF, known for its potent motility-inducing properties^49^, promoted an increased scattering of cells away from the origin when delivered by hPL-loaded HGF-TrAPs, compared with hPL-loaded HGF-scrTrAPs and the cysteine control group (Fig. 4d(i)). This increase mirrors observations made with HGF-TrAPs loaded with recombinant HGF (Supplementary Fig. 12). As time progressed, the number of migrated cells increased more significantly than in the control groups, probably due to sustained HGF release via TrAP activation as cells migrated across the coverslip surface.

Interestingly, because the TrAP system operates through a fundamentally different mechanism from other controlled-release methods, these functional assays are an ideal means of evaluating the release behaviour of TrAPs. Growth factors bound to TrAPs are maintained in an inhibited state by the high-affinity aptamers (*K*_d_ values of 0.1–20 nM; Supplementary Table 1) and are released only on mechanical unfolding by cellular traction forces. Passive release is, therefore, near or below the detection limits of standard ELISA assays, making them less reliable than functional assays. Therefore, cellular and tissue-level responses such as sprouting, cell migration, wound closure and tissue integration represent the most sensitive and effective measures of whether a biologically relevant dose is released locally at the cell–material interface on traction-force-mediated unfolding of TrAPs, since this dose is inherently cell dependent and spatially constrained, or passively released, in the case of scrTrAPs.

## TrAP-harvested growth factors promote healing of ex vivo human skin wounds

**Fig. 5:**
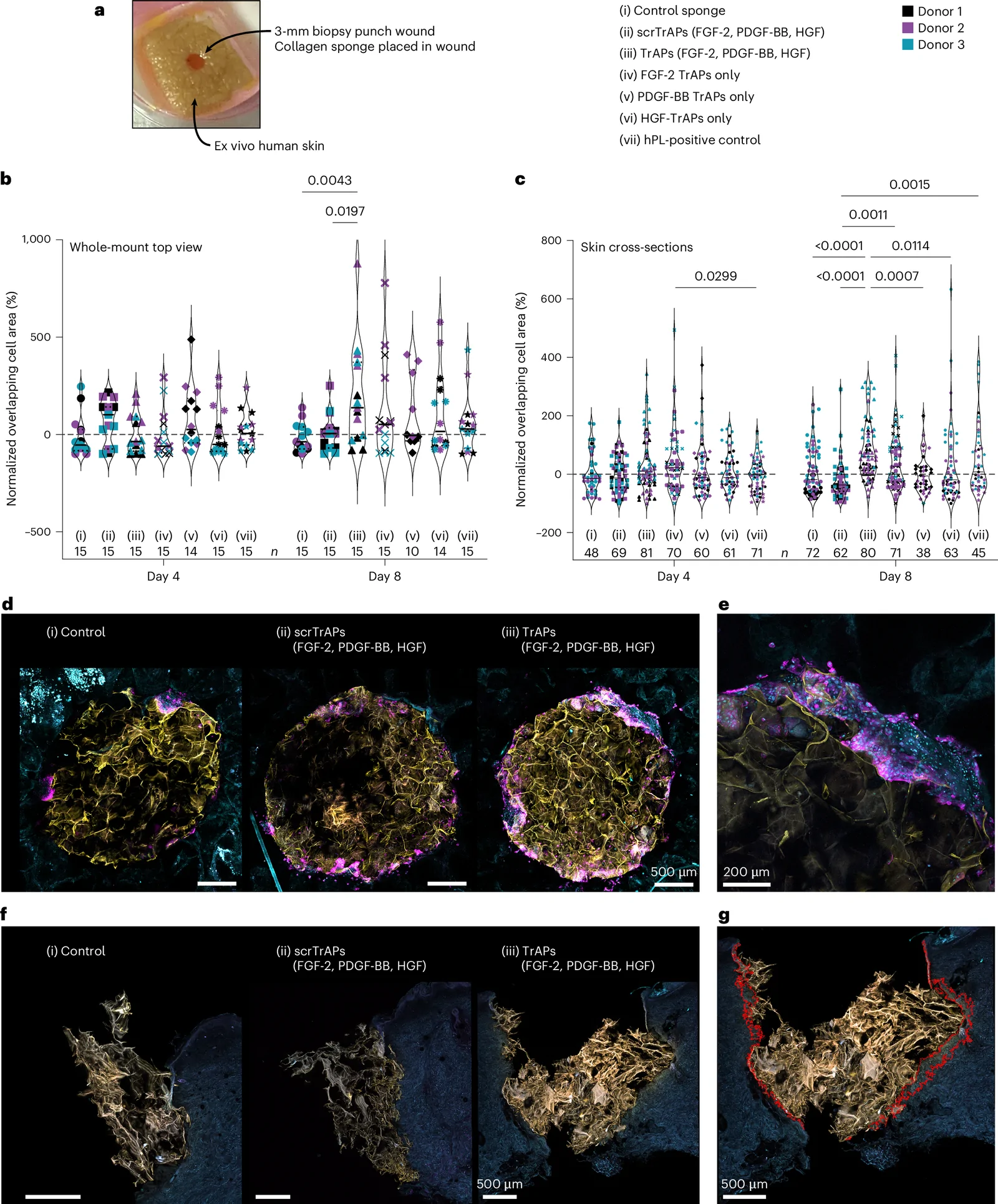
TrAP-functionalized sponges accelerate wound healing in an ex vivo human skin model. **a**, Image of full-thickness skin wound model. **b**, Quantification of the overlap between cells and sponge from fluorescence images of whole-mount skin samples, normalized to the control group on day 4 for each biological donor set. **c**, Quantification of integration between dermis and sponge, normalized to the mean integration area of the control group on day 4 for each biological donor set. **d**, Fluorescence *z*-stack overview images of the whole-mount skin wounds, day 8 post-injury (nucleus, cyan (DAPI); F-actin, magenta (phalloidin); sponge, yellow (Texas Red)). **e**, Magnified view of a cell–sponge overlap region from the combined TrAPs group shown as a maximum-intensity *z*-stack projection. **f**, Fluorescence images of the skin cross-section. **g**, Example of identification and quantification (data from **c**) of dermis–sponge integration region (red outline) using MATLAB. For **b**, the *n* values are indicated in the graphs. Samples were obtained from 4–5 wounds per biological donor, from 3 biologically independent donors, except for PDGF-BB-TrAPs (day 8), which comprised samples from 5 wounds per biological donor, from 2 biological donors. Statistical analysis was performed using restricted maximum likelihood mixed-effects model, followed by a Tukey–Kramer post hoc test. For **c**, the *n* values are indicated in the graphs. The samples were obtained from cross-sections of wounds across 3 biological donors, except for PDGF-BB-TrAPs (day 8) for which the samples were obtained across 2 biological donors. Statistical analysis was performed at each time point using one-way ANOVA, followed by a Tukey–Kramer post hoc test. Data are presented as violin plots; horizontal solid lines indicate median and horizontal dashed lines indicate quartiles. Source data

**Extended Data Fig. 2:**
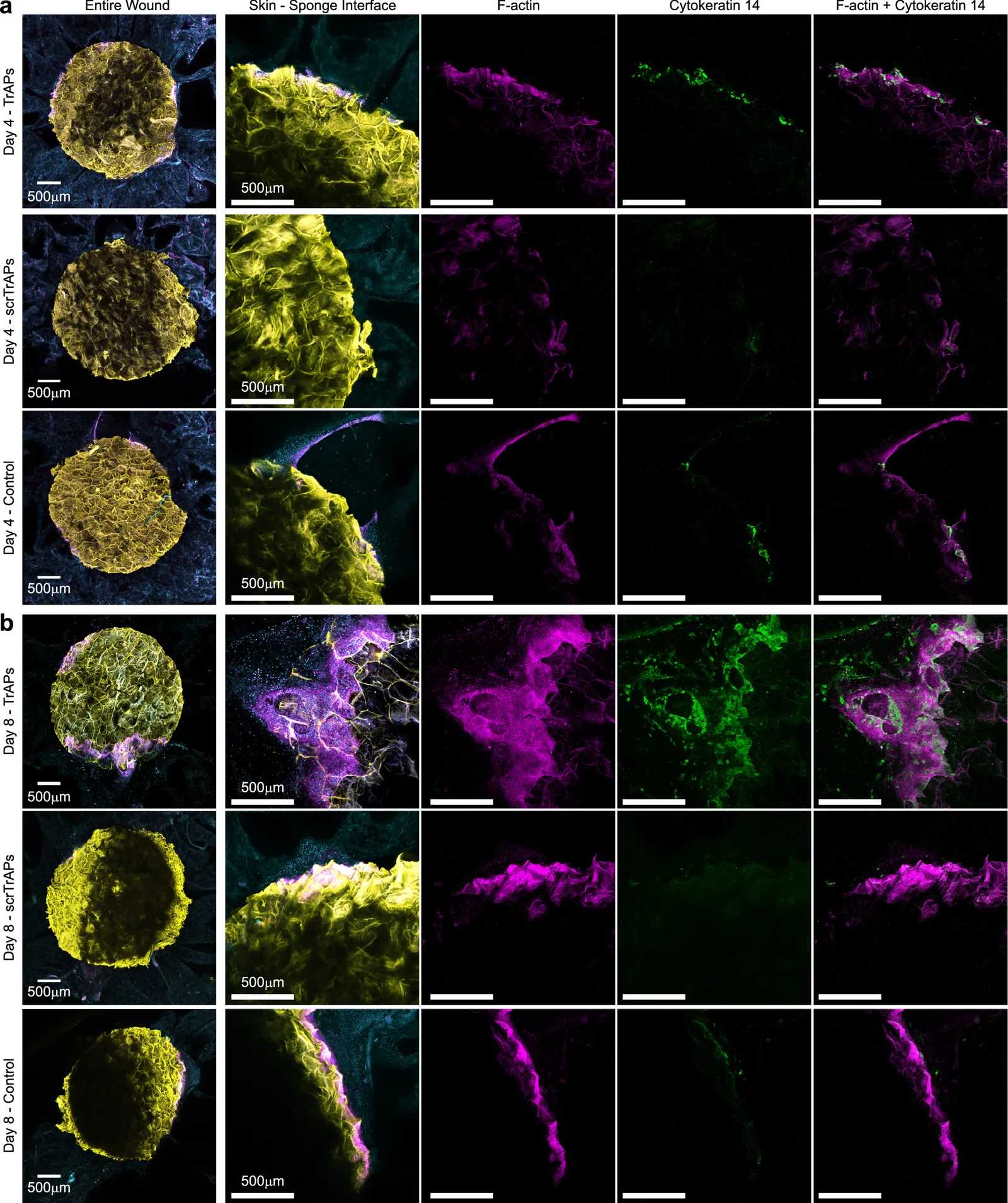
Overlapping skin-sponge interface includes migrating keratinocytes expressing cytokeratin 14. **a, b**, Fluorescence *z*-stack overview images of whole-mount ex vivo skin wounds at day 4 (**a**) and day 8 (**b**) post-injury. Regions of skin-sponge interface are shown at higher magnification in the adjacent panels (DAPI: cyan, F-actin: magenta, Texas Red-tagged sponge: yellow, Cytokeratin-14: green).

**Extended Data Fig. 3:**
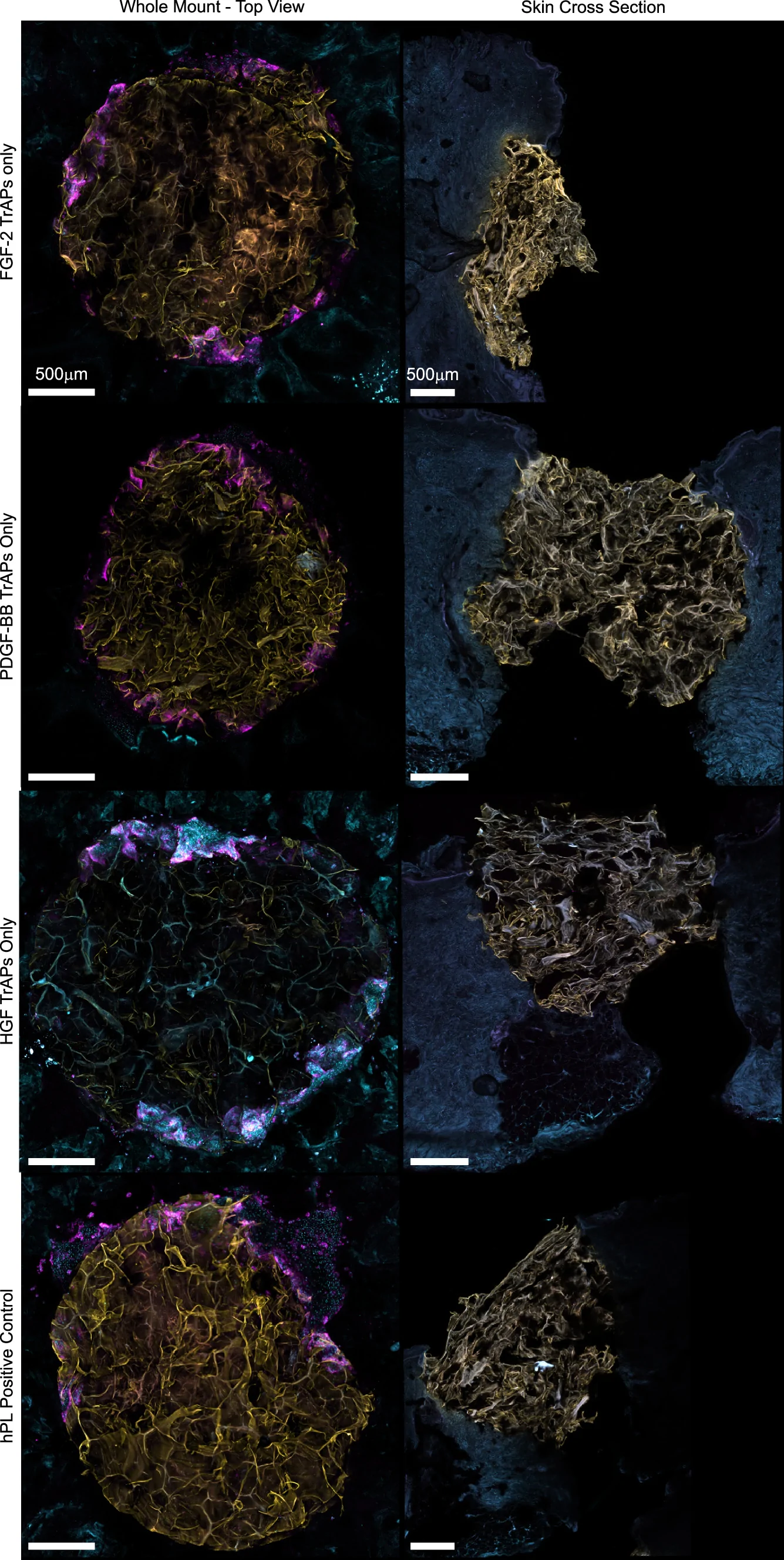
Fluorescence images of sponge-skin integration in ex vivo human skin wound model, day 8 post-injury. Additional images corresponding to experimental groups in Fig. 5d and Fig. 5f. Fluorescence *z*-stack images of skin wounds, whole-mount and cross-sectioned, day 8 post-injury (DAPI: cyan, F-actin: magenta, Texas Red-labeled sponge: yellow).

Next, we validated the use of endogenous growth factors in a translationally relevant ex vivo human skin model to build on the in vivo rat studies and demonstrate their functionality in human tissue. We first assessed different combinations of TrAPs delivering two or more endogenously harvested growth factors from hPL, both in vitro and in ex vivo human skin (Supplementary Figs. 17–19). Both assays demonstrated quantifiable benefits of using an hPL-loaded multi-TrAP system. Following that, a combination of FGF-2, PDGF-BB and HGF was harvested from hPL using functionalized collagen sponges and used to treat wounded skin from three different biological donors (Fig. 5). In the human skin model, the optical fluorescence imaging of whole-mount skin from the epidermal surface revealed an increased overlap of F-actin- and DAPI-stained cells with the fluorescently labelled sponge, 8 days after treatment with a combination of hPL-loaded FGF-2, PDGF-BB and HGF-TrAPs, compared with the combined hPL-loaded scrTrAPs and cysteine control groups (Fig. 5b,d,e). Immunostaining for cytokeratin 14 further confirmed the presence of migrating keratinocytes at the overlapping tissue–sponge interface (Extended Data Fig. 2). These observations suggest that the localized delivery of multiple endogenous growth factors can effectively enhance and direct cell migration and proliferation towards the functionalized sponge’s centre, corresponding to the centre of the wound bed. Although there was no statistical significance between the combined hPL-loaded TrAPs group and the individual hPL-loaded TrAPs group (Fig. 5b and Extended Data Fig. 3), a trend towards a higher normalized overlapping cell area was observed in the combined hPL-loaded TrAPs group by day 8 (Fig. 5b,d). This lack of significant difference may be due, in part, to the limited imaging depth of a few hundred micrometres in a multi-millimetre-thick whole skin sample, restricting the ability to fully elucidate the total contribution of each growth factor. Hence, complementary quantification methods were also performed on thinner, 200 µm-sectioned skin samples (Fig. 5c,f,g).

**Extended Data Fig. 4:**
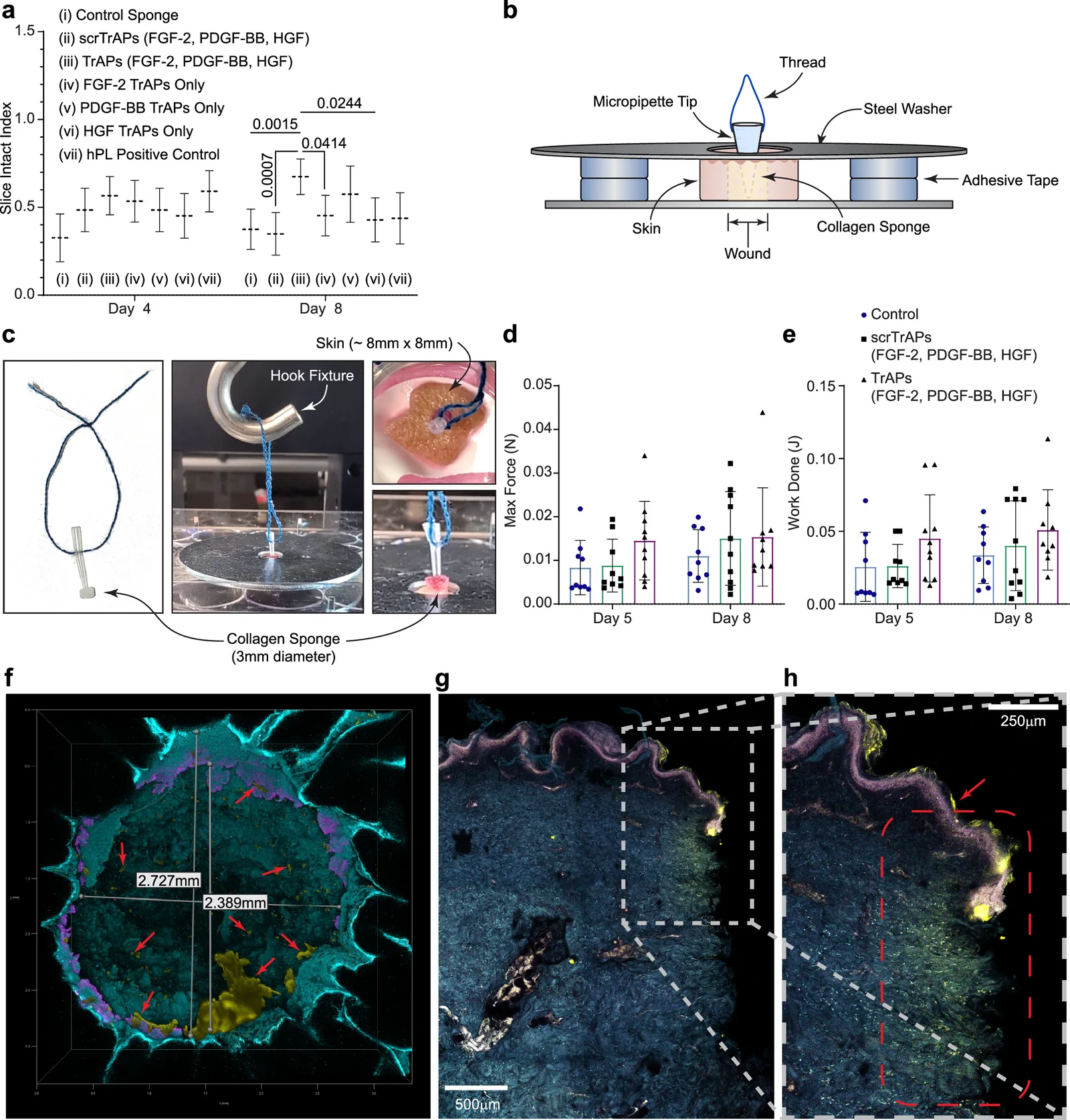
Mechanical assessment of skin-sponge integration in ex vivo human skin wounds. **a**, Probability of 200 µm skin cross-sections remaining intact during microtome sectioning. Skin sections where the sponge remained fully intact were given an index of 1, while a sponge that tore was given an index of 0. **b**, Illustration of tensile test set-up. A steel washer was used to secure the skin sample while tensile force was applied through a thread attached to a micropipette tip embedded within the implanted sponge. **c**, Images of tensile pull-out test set-up. **d, e**, Maximum pull-out force (**d**) and work done (**e**) during sponge pull-out at days 5 and 8 post-injury. **f**, Top view of three-dimensional reconstructed image of the whole mount skin wound after sponge pull-out, as captured with a confocal fluorescence microscope (DAPI: cyan, f-actin: magenta, Texas Red-labeled sponge: yellow). Red arrows indicate residual sponge fragments within the skin wound. **g**, Fluorescence *z*-stack overview image of skin cross-section (DAPI: cyan, F-actin: magenta, Texas Red-labeled sponge: yellow). **h**, Magnified view of skin cross-section. Red arrow indicates residual collagen sponge at wound edge. The red outline highlights a region of overlap between skin tissue and collagen sponge fragments. For **a**, Day 8: n = 72 (control), n = 63 (combined FGF-2 + PDGF-BB + HGF-scrTrAPs), n = 87 (combined FGF-2 + PDGF-BB + HGF-TrAPs), n = 75 (FGF-2 TrAPs), n = 40 (PDGF-BB TrAPs), n = 63 (HGF-TrAPs), n = 48 (hPL Positive Control) skin sections. Statistical analysis was performed using one-way ANOVA, followed by Šídák post hoc test. Data are presented as mean with 95% confidence intervals. For **d**, **e**, day 5: n = 9 (Control, combined scrTrAPs), n = 10 (combined TrAPs). Day 10: n = 9 (Control, combined TrAPs), n = 10 (combined scrTrAPs) skin wound samples. Statistical analysis was performed using two-way ANOVA followed by Tukey-Kramer post-hoc test. Data are presented as Mean ± SD. Source data

The examination of the 200 µm skin cross-sections revealed significantly enhanced integration of skin with the functionalized sponge in the combined hPL-loaded TrAPs group compared with all groups, except the hPL-loaded FGF-2-TrAPs and hPL-positive treatment group, on day 8 (Fig. 5c,f,g and Extended Data Fig. 3). Given the established roles of FGF-2 and PDGF-BB in promoting fibroblast proliferation and migration, respectively, and of HGF in inducing epithelial migration, it is unsurprising that the combined delivery of these growth factors facilitated greater skin integration, particularly in cross-sectional slices encompassing the dermal region. Interestingly, cross-sectional slices from the combined hPL-loaded TrAPs group were more likely to remain intact during sectioning compared with control groups in which tearing frequently occurred within the sponge or at the sponge–skin interface (Extended Data Fig. 4a). This suggests that the combined hPL-loaded TrAPs may facilitate greater integration between the sponge and the healing skin. Additional tests (Extended Data Fig. 4b,c) were also conducted to investigate the mechanical properties of the sponge–skin interface. However, the interplay between the degradation of collagen sponge in which new tissue ingress into and the fragile matrix of healing tissue on day 8 made it difficult to identify any correlation between the mechanical properties and the observed degree of skin integration in this particular experiment (Extended Data Fig. 4d–h). Nonetheless, the above-mentioned findings, when taken together, suggest that harvesting and delivering multiple endogenous growth factors via TrAPs can enhance skin integration by promoting cell migration and proliferation within a hPL-loaded TrAP-functionalized collagen matrix. This improved integration highlights the potential of TrAP technology for tissue repair applications.

## TrAP-harvested growth factors promote in vivo wound healing in mice

**Fig. 6:**
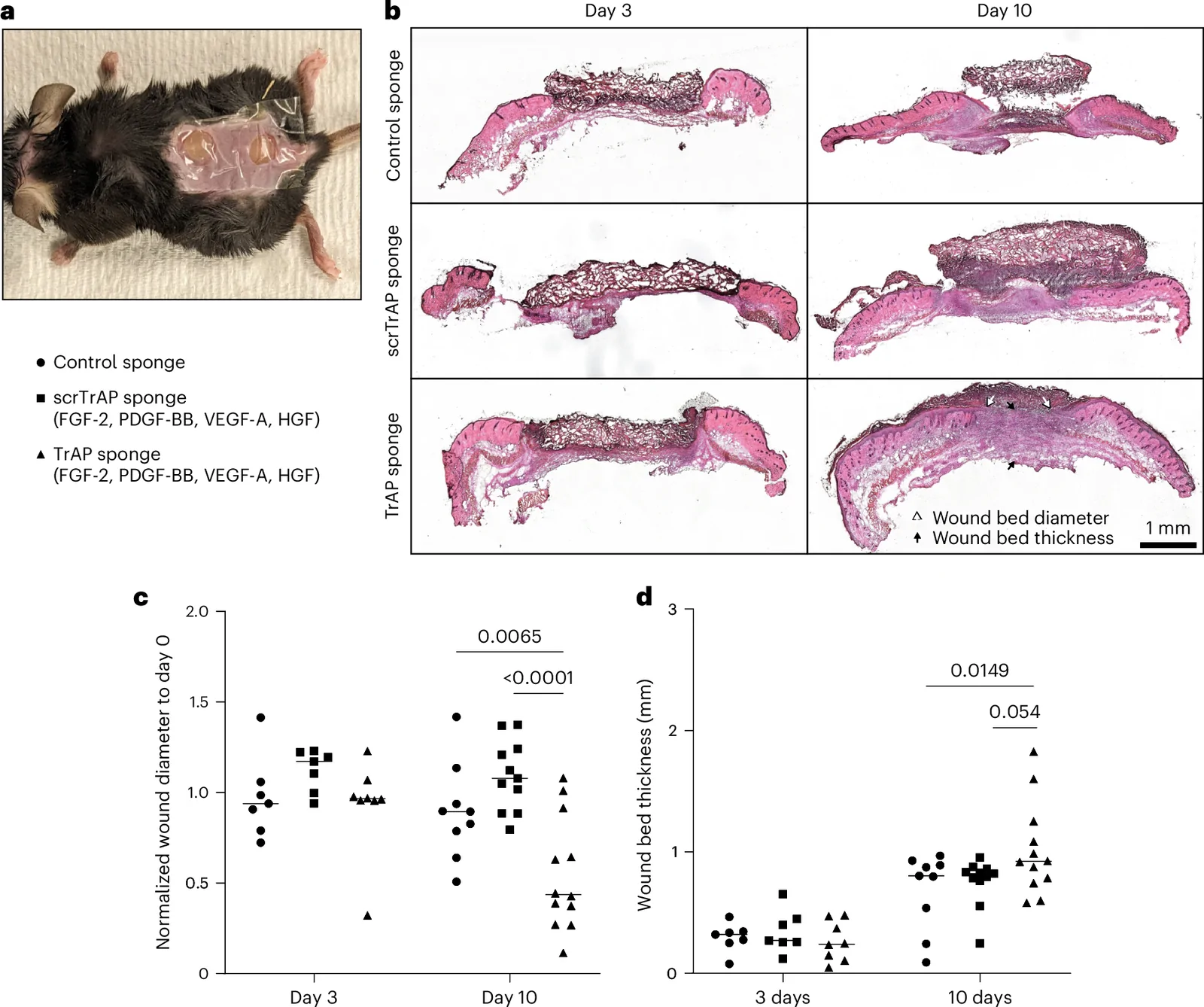
TrAP-functionalized collagen sponges accelerate in vivo skin wound healing in mice. **a**, Control, scrTrAP-functionalized or TrAP-functionalized collagen sponges were implanted within full-thickness mouse skin wounds. **b**, Haematoxylin and eosin-stained cross-sections obtained from the centre of the wound on days 3 and 10 post-treatment. **c**, Quantification of wound diameter measured from the wound cross-section and normalized to the mean wound diameter on day 0 for each wound. **d**, Quantification of wound bed thickness measured beneath the implanted sponge. **c**,**d**, For day 3, *n* = 7 wounds (control and scrTrAPs), and *n* = 8 wounds (TrAPs), obtained from 2 male mice and 2 female mice per group. For day 10, *n* = 9 wounds (control), *n* = 11 wounds (scrTrAPs) and *n* = 12 wounds (TrAPs), obtained from 3 male mice and 2 female mice (control) or 3 male mice and 3 female mice (scrTrAPs and TrAPs). Wounds in which the sponge had fallen off during the study were excluded from the analysis. Statistical analysis was performed using two-way ANOVA, followed by a Tukey–Kramer post hoc test. Data are presented as individual points; horizontal lines indicate the median. Source data

Building on insights from the ex vivo skin model, the combined use of TrAPs to harvest endogenous growth factors was further validated using an in vivo mouse skin model. In this study, TrAPs and scrTrAPs of VEGF-A, HGF, FGF-2 and PDGF-BB were functionalized on collagen sponges to capture growth factors from hPL, similar to the ex vivo skin experiments. Two 6 mm full-thickness excisional wounds were created on the dorsum of male and female mice in which each wound was treated with a 6 mm collagen sponge functionalized with either cysteine control, combined hPL-loaded scrTrAPs or combined hPL-loaded TrAPs (Fig. 6).

After 10 days, the wounds treated with the hPL-loaded TrAPs showed a significant reduction in normalized wound diameter compared with the hPL-loaded scrTrAPs and cysteine control group, whereas no significant difference was observed between the scrTrAPs and cysteine control group (Fig. 6b,c). In addition, a trend towards increased wound bed thickness (Fig. 6d), suggesting the promotion of tissue growth, was also observed. On day 3, label-free multiphoton imaging at the centre of the hPL-loaded TrAP-functionalized sponge showed a trend towards increased autofluorescence in the 525 ± 20 nm channel (Supplementary Fig. 20) in response to 855 nm excitation, compared with the control groups. Taken together with observations from the in vivo mouse study and ex vivo human skin model, this autofluorescence may suggest, although unproven, potential cell infiltration^50^. This increase in cell integration at the early stages of wound healing could perhaps have contributed to the higher probability of sectioned sponges remaining intact in the combined hPL-loaded TrAPs group, as observed in the ex vivo skin study (Extended Data Fig. 4a), through a more integrated sponge interface. Overall, the in vivo mouse study corroborates and extends the findings from the ex vivo skin model, demonstrating that TrAPs can successfully harvest and deliver a combination of endogenous growth factors from hPL to promote tissue repair. These findings suggest that TrAP-facilitated growth factor delivery can enhance tissue regeneration in complex tissues, such as skin, across species.

## Discussion

This study establishes cellular traction forces as a viable intrinsic trigger for controlled therapeutic delivery in wounded tissues, both in vivo and ex vivo, using a materials-based strategy. TrAPs (preloaded and empty) resisted degradation and retained functional integrity in enzyme-rich wound environments long enough to promote vascularization in rat femurs and reduce wound diameter in mouse skin models, even without nuclease-resistant modifications beyond the inherent 3′ and 5′ modifications needed to create TrAPs. This is a particularly interesting result since unmodified DNA oligonucleotides typically have in vivo half-lives of minutes to hours in biological fluids. For instance, DNase I-mediated degradation in serum follows first-order kinetics with a reported half-life of approximately 30 min^51^, and unmodified aptamers generally have very short biological half-lives in vivo^52^. By contrast, in this work, TrAPs retained functional integrity across multiple species and tissue types, long enough to exert a lasting biological impact. We propose that the matrix-bound configuration of TrAPs, anchored to the collagen scaffold at one end and to the cell-adhesive peptide at the other, confers protection analogous to that of matrix-bound proteins, which are substantially more resistant to proteolytic degradation than their soluble counterparts^53^. This finding has important implications beyond the TrAP system, demonstrating that in some contexts, DNA-based therapeutics in wound and tissue repair may not require expensive nuclease-resistant chemical modifications.

The data showing that scrTrAPs result in significantly smaller vessels (Fig. 2c) and have no significant effect on wound diameter compared with controls (Fig. 6c) further support the idea of enzyme protection by demonstrating that scrTrAPs resist degradation and continue to inhibit growth factor signalling in vivo. In particular, because cellular traction force is required to activate bound growth factors, the liberation of TrAPs from the local matrix site due to substrate degradation eliminates the ability to subsequently trigger force-mediated release. This feature positions the delivery strategy to offer a notable advantage over current clinical approaches (for example, Regranex, INFUSE and Fiblast) that inherently introduce supraphysiological concentrations of active proteins into the body.

Even under the conservative assumption of 100% growth factor retention from hPL, which represents a theoretical upper bound due to competitive matrix binding, steric hindrance, growth factor degradation and wash steps reducing actual loading, TrAP-enabled growth factor doses are orders of magnitude lower than current clinical and experimental standards and delivered as a single treatment (see the ‘Implications for maximum TrAP loading’ section in the Supplementary Information and Supplementary Table 10). For comparison, the INFUSE bone graft (BMP-2) delivers approximately 1.4 mg cm^−3^, FGF-2 therapy for leg ulcers requires approximately 540,000 ng cm^−3^ over 3 months, and other engineered systems for growth factor delivery^54–56^ are tens to hundreds of times higher. In practice, effective doses are lower still, as growth factors are released locally and transiently at the point of cellular engagement rather than passively diffusing throughout the wound environment (see the ‘Implications for maximum TrAP loading’ section in the Supplementary Information).

These findings have implications for clinical translation and quality control. For conventional growth factor delivery systems, quality control involves measuring the loaded dose, as the drug is active on release. By contrast, TrAPs are a controlled delivery system that captures patient-derived proteins, therapeutically activating them by subsequent cell-mediated interactions at the point of contact, a process that is inherently dynamic and patient-specific. This is not a unique challenge; patient-derived approaches such as platelet-rich plasma lack a defined delivered dose, with growth factor concentrations varying between donors and preparations, and release remaining passive and uncontrolled. However, TrAPs offer a more defined framework, since aptamer concentrations are controlled at manufacture and the upper-bound loading is calculable. Functional tissue response, used throughout this study, provides a potency-based quality read-out consistent with Food and Drug Administration and European Medicines Agency guidance for advanced cell and gene therapy products^57–60^, which establishes functional biological activity, rather than physicochemical characterization alone, as the standard for demonstrating that a product is performing as intended.

Critically, no major adverse events led to early study termination in any of the tested rats or mice. Although toxicity and immunogenicity were not the primary objectives of this study, no indications of either were observed at the injury site. Future studies must further explore these aspects to ensure that TrAPs and other DNA nanotechnologies do not trigger unintended immune priming or responses and conduct dose studies to ensure optimal amounts of growth factors are delivered. Additionally, future longitudinal studies should quantify more mechanistic and functional metrics (for example, collagen alignment, remodelled skin morphology, appendages and elasticity) in fully healed skin several weeks post-injury to better understand the functional improvements TrAPs might provide.

This study also did not examine how cell-secreted extracellular matrix^61,62^ or matrix remodelling dynamics influence TrAP activation. However, results demonstrate that cells can access the loaded growth factors to modulate tissue repair, probably by activating TrAPs on interaction as they migrate through the scaffold before extensive extracellular matrix deposition or remodelling potentially hinders access. Matrix remodelling processes, such as extracellular matrix deposition and protease-mediated degradation, may nonetheless dynamically alter the mechanical and structural environments around TrAPs over time, potentially affecting the efficiency and spatiotemporal control of growth factor delivery in ways that are cell type dependent and context dependent. Future studies should, therefore, investigate how remodelling processes influence TrAP activation and how scaffold design parameters such as crosslinking dynamics, degradation kinetics and stiffness can be optimized accordingly.

Using high-specificity, high-affinity aptamers, TrAPs have also demonstrated the ability to transform biomaterials into affinity columns, capable of selectively harvesting proteins from biological fluids. This feature was validated by selectively extracting growth factors from commercially sourced hPL, a proof of concept for the potential of using autologous blood sources on site. By enabling the use of patient-derived blood as a source of growth factors, TrAPs may simplify the regulatory processes and cold-chain logistics associated with manufactured, recombinant proteins. With DNA aptamers’ long shelf-life at room temperature^63^ and the potential to load autologous growth factors at the treatment site, TrAPs may expand therapeutic accessibility in resource-limited settings and frontline environments (for example, remote/battlefield).

Overall, this study establishes cellular traction forces as a viable trigger for controlled therapeutic delivery in vitro, in vivo, ex vivo and across species, providing the initial demonstration of this mechanism in complex, repairing tissues. The multidimensional results of this work bring together the convergence of five features that, to our knowledge, have not been reported in combination: (1) a force-responsive biomaterial that captures endogenous growth factors from the injury milieu and human blood, eliminating the need for manufactured recombinant proteins; (2) high-affinity aptamer binding that holds growth factors in an inhibited state in vivo until mechanically activated; (3) cell-activated release at the point of cellular contact, creating spatially localized delivery at physiologically relevant doses, which are orders of magnitude lower than current clinical standards; (4) functional integrity of unmodified oligonucleotide constructs in enzyme-rich wound environments across multiple species; and (5) compatibility with virtually any biomaterial scaffold and any growth factor for which a high-affinity aptamer exists, enabling a modular, combinatorial approach to biologic therapy. By using aptamers as the basis for TrAPs, over 30 years of aptamer research can be leveraged to enable thousands of customizable delivery systems through simple chemical synthesis^64^.

This approach bridges the biotic–abiotic interface, enabling cell-specific, autonomous therapeutic activation^27^ across existing clinical biomaterials and devices. More broadly, the substantial global aptamer portfolio and knowledge base provides a foundation for the rapid development of cell-responsive biomaterials that can efficiently deliver defined biologic therapies across diverse clinical indications.

## Methods

### Ethics statement

Human skin tissue was collected from individuals who provided informed consent using consent forms approved by the Imperial College Research Ethics Committee (REC#21IC6723) and managed through the Imperial College Healthcare Tissue Bank (Human Tissue Authority licence 12275).

ARRIVE Guidelines 2.0 for animal research were followed for study planning, conduct and writing. The rat femur study was performed in accordance with the Animals (Scientific Procedures) Act, 1986 (UK), with approval from the Animals in Science Regulation Unit of the UK Home Office under project licence PD51CDDA3 and local approval from Imperial College London’s Animal Welfare and Ethical Review Body. The mouse skin wound study was performed using protocols approved by the University of Arkansas Institutional Animal Care and Use Committee (IACUC; 23034).

Both mice and rats were housed with littermates until the surgical procedure with free access to food and water in a room with a 12 h light/dark cycle, ambient temperature of 19–26 °C, 30%–70% humidity and standard contact bedding. Following surgery, mice and rats were housed individually to prevent interference of other mice/rats with the bandage and healing process.

### DNA aptamer synthesis

Aptamers were synthesized with an in-house MerMade 6 Oligonucleotide synthesizer, using solid-phase phosphoramidite chemistry^27^. The synthesis scale was 1 µmol, and all unmodified DNA phosphoramidites compatible with UltraFAST deprotection (Bz-dA-CE, Ac-dC-CE, dmf dG-CE and dT-CE) were purchased from LGC Genomics. The 3′ end of the modified sequence was conjugated to a thiol using a 3′-thiol-modifier C3 S-S CPG solid support, and the 5′ end was modified with a 5′-bromohexyl phosphoramidite^27^. Following synthesis, the 5′-bromohexyl was converted to 5′-azidohexyl using a mixture of 13 mg of sodium azide and 30 mg of sodium iodide in 2 ml of *N*,*N*-dimethylformamide. The mixture was left to react for 1 h at 65 °C. Once cooled, the oligos were rinsed twice with DMF and acetonitrile. Subsequently, oligos were cleaved from the solid support and the protecting groups removed using aqueous ammonium hydroxide and methylamine (1:1) at 65 °C for 10 min. The oligos were purified using ethanol precipitation, where 50 µl of 3 M sodium acetate and 700 µl of 100% ethanol is added to the oligo supernatant and incubated overnight at –20 °C. The DNA precipitate is then collected through centrifugation at >12,000*g* for 15 min, rinsed with 70% ethanol and resuspended in sterile water. The concentration of oligos was measured using a NanoDrop. scrTrAPs and TrAPs were produced by conjugating DBCO-modified G**RGD**SPC peptides and G**RDG**SPC peptides, respectively, to the 5′-azidohexyl ends of the aptamer. Aptamers were synthesized for VEGF-A (TTA GGG CCA CGT CTA TTT AGA CTA GAG TGC AGT GGT TC TTTTT), PDGF-BB (TTT TTC AGG CTA CGG CAC GTA GAG CAT CAC CAT GAT CCT G TTTTT), FGF-2 (GGT GCT AAC CAG GAC ACA CC), HGF (TTG CGC CAG CTT TGC TGA TGG GTG GCC ACC CTT GCC CTG GGT TTG AAT TTC GAT CCT ATC G TTT) and a random non-binding single-stranded DNA (GAT CCA AGT AGC GTT TGA CGT ATG GCT ATA GCA TGG CTC G TTT) (Supplementary Table 1).

### Culturing HMEC-1 cells on microcarrier beads

Cytodex 3 microcarriers (Cytiva) were rehydrated according to the manufacturer’s instructions in phosphate-buffered saline (PBS), autoclaved and rinsed in endothelial cell growth medium MV 2 (ECGM MV2, PromoCell). Passages 3–5 HMEC-1 (CRL-3243, ATCC) were cultured on Cytodex 3 microcarrier beads at 50 cells:1 bead ratio in a Falcon 5 ml round-bottom polystyrene test tube. Gentle pipetting was performed hourly on the microcarrier-cell solution, for the first 5 h, to allow for the uniform adhesion of cells on the surface of the beads. The microcarrier-bead solution was subsequently transferred to a T25 flask and incubated overnight on a rocker shaker at 37 °C and 5% CO_2_, in fully supplemented ECGM MV 2.

### Angiogenic spouting assay with TrAP-functionalized collagen gels

The first layer of 30 µl, 3.85 mg ml^−1^ collagen type I hydrogel (from rat tail, Roche) was prepared according to the manufacturer’s protocol and pipetted onto a 20 mm × 2.5 mm U-shaped capillary tube (Leica). The hydrogel was allowed to polymerize in humidified conditions at 37 °C and 5% CO_2_ for at least an hour. Once the first layer of hydrogel was polymerized, the microcarrier-cell solution was transferred to a Falcon 5 ml round-bottom polystyrene test tube and rinsed once with Dulbecco’s phosphate-buffered saline (DPBS), once with serum-reduced ECGM MV2 media (Basal Medium MV2 with 0.2% FBS, 1 µg ml^−1^ of ascorbic acid, 0.2 µg ml^−1^ of hydrocortisone, 100 U ml^−1^ of penicillin–streptomycin, 2 mM of GlutaMax) and resuspended in 1 ml of serum-reduced ECGM MV2 media. Then, 18 µl of the cell-bead solution was pipetted onto the first layer of the hydrogel. The assay was then incubated for 1.5 h at 37 °C and 5% CO_2_, followed by 30 min of incubation under a biosafety fume hood, to allow the gentle evaporation of excess media. TCEP-reduced TrAPs, scrTrAPs or cysteine were mixed with collagen hydrogel to form 3 mg ml^−1^ of 1 µM scrTrAP-, TrAP- or cysteine-functionalized collagen gel (Supplementary Table 4). Then, 30 µl of the gel solution was gently pipetted onto the capillary tubes and incubated for 1 h in the humidified condition at 37 °C and 5% CO_2_ (Supplementary Fig. 4). Furthermore, 2 ml of 100 ng ml^−1^ of VEGF-A_121_ in serum-reduced ECGM MV2 media was pipetted into 12-well plates (except for the empty scrTrAPs and empty TrAPs group). Capillary tubes were transferred to the 12-well plates and incubated for 30 min. Finally, the capillary tubes were gently washed five times, by transferring the tubes into filled wells of serum-reduced ECGM MV2 media, with each wash lasting at least 3 min. The bead sprouting assay was incubated in serum-reduced media and imaged at 24 h, 48 h and 72 h (Supplementary Fig. 4).

### Imaging and quantification of endothelial cell sprouts

For live imaging, images of the beads were captured using an inverted Leica DM IL LED microscope bright-field microscope. For end-point imaging, cells were fixed overnight in 4% paraformaldehyde. The capillary tubes were washed three times with DPBS the following day and incubated with 1.5 ml of 1:500 dilution of TRITC-conjugated phalloidin (Sigma, FAK100) in 12-well plates for 1.5 h. The capillary tubes were then washed twice in DPBS and incubated for 30 min with 0.1 μg ml^−1^ of DAPI. The capillary tubes were washed once and subsequently set up for imaging with a Leica STELLARIS 5 Light Sheet Microscope (Supplementary Fig. 5). Bright-field images were analysed using MATLAB (v.R2023b) and the fluorescence *z*–stacks were maximum-intensity projected in FIJI (v.1.54) before analysis. Sprout lengths (Supplementary Fig. 6) and thickness were manually measured using the MathWorks Image Processing Toolbox ‘Measure Distances in an Image’ example (MathWorks; https://uk.mathworks.com/help/images/measure-distances-in-images.html), with a pixel-to-micrometre calibration factor applied for each image. Measurements were normalized to the scrTrAPs group within each experiment.

### Functionalization of collagen sponges

Sterile collagen sponges (Avitene; 3, 4, 5 or 6 mm in diameter depending on the study) were functionalized with 1 µM of TrAPs, scrTrAPs or cysteine using *N*-ethyl-*N*′-(3-dimethylaminopropyl)carbodiimide hydrochloride/*N*-hydroxysulfosuccinimide sodium salt-mediated crosslinking and maleimide–thiol coupling^27^. For in vitro, in vivo rat and ex vivo human skin studies, sponges were first tagged with 18.4 µM of Texas Red-X succinimidyl ester in 200 mM of PBS for 2 h at room temperature; this step was omitted for the in vivo mouse study. Sponges were then crosslinked using 11.5 mg ml^−1^ of *N*-ethyl-*N*′-(3-dimethylaminopropyl)carbodiimide hydrochloride, 5.19 mg ml^−1^ of *N*-hydroxysulfosuccinimide sodium salt and 3.18 mg ml^−1^ of 1-(2-aminoethyl)maleimide hydrochloride in PB:PBS (1:15 parts) buffer for at least 12 h on a shaker, at room temperature. After thorough washing, reduced 1 µM of TrAPs or scrTrAPs containing a terminal thiol were added to the sponges and allowed to conjugate to the maleimide groups for at least 3 h on a shaker, at room temperature. Multi-TrAP and multi-scrTrAP systems were functionalized at 1 µM of each type of scrTrAPs/TrAPs. The scaffolds were then washed seven times with DPBS.

Functionalized collagen sponges were blocked with 1% bovine serum albumin for at least 30 min in 96-well plates to prevent the non-specific binding of proteins. For growth factor-loaded scaffolds, sponges were incubated overnight at 4 °C with recombinant growth factor at 200 ng ml^−1^. For hPL-loaded scaffolds, sponges were incubated overnight at 4 °C with hPL diluted in PBS (in vitro and ex vivo skin study, 33.3%; in vivo skin wound, 50%). All groups, including controls, within an experiment were subjected to the same growth factor or hPL-loading conditions. Sponges were washed at least three times in DPBS. For sponges loaded with hPL, sponges were washed for at least 15 min per wash, at 37 °C. All solutions for in vivo studies were sterile filtered through a 0.22 µm filter before use.

### In vivo rat femur model

Female Wistar rats were purchased at ages of 5–9 weeks from Charles River Laboratories and allowed to acclimatize for 1 week before surgery. The study was conducted under UK Home Office approval (see the ‘Ethics statement’ section), and was classified as moderate severity. A power calculation was performed at the licence application stage to estimate the minimum group sizes, assuming a difference of means of 40% based on the published literature, a common standard deviation of 27%, a statistical power of 0.90 and a type 1 error rate of 0.05. As the study remained exploratory in nature and precise effect sizes for the TrAP system were not known a priori, the sample sizes were additionally guided by the 3Rs principles to minimize animal use; exact group sizes are reported in the relevant figures. Animals were randomly allocated to experimental groups before surgery, and outcome assessment and data analysis were performed blinded to group allocation, in accordance with refinement provisions.

On the day of surgery, rats were anaesthetized with 1.5% isoflurane at a flow rate of 1 l min^−1^. Hair from the right hindquarter was clipped and the area was cleaned with 4% chlorhexidine surgical scrub for 5 min. Before surgery, rats were dosed subcutaneously with Ceporex (MSD Animal Health, 15 mg kg^−1^), Vetergesic (Ceva Animal Health, 0.05 mg kg^−1^) and Metacam (Boehringer Ingelheim, 1 mg kg^−1^). Marcaine 5% (Aspen Pharma) was also administered locally on the hindlimb. All drugs were purchased from MWI Animal Health UK. An incision was made through the skin of the hindlimb along the line of the femur, followed by a blunt dissection of the muscle to expose the bone. A polyetheretherketone (PEEK) plate was placed adjacent to the femur and loosely fixed with 6 × 1 mm^2^ self-tapping cortical bone screws, before cutting a 6 mm section of the bone with an oscillating saw. The screws holding the PEEK plate were then tightened and sponges were placed in the gap (or left empty). The muscle and skin were closed with Vicryl Rapide sutures. Rats were given Vetergesic (0.05 mg kg^−1^) every 12 h orally for 3 days post-surgery. Experimental groups included empty gap (no sponges), empty sponges (no TrAPs), VEGF-TrAPs and VEGF-scrTrAPs.

Animal well-being was monitored by keeping a log of the animals’ weight and observing any changes in behaviour and overall look (that is, the state of fur, movement of whiskers and so on). No changes were observed in any of the groups that would indicate distress or pain in the animals. The average weight of the rats in the control group was significantly lower than that of the VEGF-TrAP group from time 0 (Supplementary Fig. 8a), but the fold increase over time is statistically non-significant for all groups (Supplementary Fig. 8b), indicating that none of the treatments elicit toxicity in the rats. All adverse effects and complications are described in Supplementary Table 5. Overall, the procedure was safe and had very few associated complications, as well as no mortality.

In the first experiment, rats were culled at 3 weeks and femurs were excised and fixed in 4% PFA for immunohistochemistry to assess angiogenesis. In the second experiment, CT scanning was performed at 3, 6, 9 and 12 weeks post-surgery to assess the bone volume fraction (BV/TV) as a measure of mineralized bone. For CT imaging, rats were anaesthetized with 1.5% isoflurane at a flow rate of 1 l min^−1^ and positioned in the CT scanner in the lateral position with the operated femur facing up. To minimize image distortion induced by the screws, the hindlimb was positioned perpendicular to the movement of the fan beam and affixed with tape.

### Bone histology

Fixed femurs were decalcified in 10% formic acid for 3 days, followed by 30% sucrose overnight, before flash freezing them in Tissue-Tek optical cutting temperature compound. For immunohistochemistry studies, a cryotome was used to obtain 10 µm cross-sections of the gap. Samples were then permeabilized in acetone at –20 °C for 10 min and rehydrated in Tris-buffered saline buffer. Antigen retrieval was performed with proteinase K in Tris–EDTA buffer buffer for 20 min at 37 °C, followed by blocking with hydrogen peroxide for 15 min and then with 10% goat serum and 1% bovine serum albumin for 1 h. Samples were incubated with primary antibodies at 4 °C overnight, washed and incubated with secondary antibodies for 1 h. Specifically, samples incubated with primary anti-CD31 (Abcam, ab182981, 1:1,000), were then incubated with goat anti-rabbit (Abcam, ab97049, 1:500). Samples incubated with anti-vWF (Invitrogen, MA5-14029, 1:25) and anti-α-SMA (Abcam, ab7817, 1:2,000) were subsequently incubated with goat anti-mouse (Abcam, ab6788, 1:2,000). Finally, samples were incubated with streptavidin–horseradish peroxidase solution (Abcam, ab64269), developed with 3,3′-diaminobenzidine (Abcam, ab64238), counterstained with haematoxylin solution and finally mounted with aqueous media. Sections were imaged with an inverted microscope, and ImageJ (v.1.54) was used to analyse the number and area of blood vessels.

### Coverslip functionalization

Here, 5 mm or 10 mm circular coverslips (Agar Scientific) were cleaned using Piranha etch, functionalized with 3-aminopropyl(diethoxy)methylsilane, rinsed with isopropanol and ultrapure water, and baked for 1 h at 80 °C. Maleimide groups were introduced to the coverslips through subsequent incubation of 40 mM of maleimide-PEG2-succinimidyl ester, in borate buffer (pH 8.5). The thiols of reduced cysteine, scrTrAPs, TrAPs, G**RGD**SPC and G**RDG**SPC peptides were then conjugated to the maleimide groups on the coverslips, at a ratio that ensures 25 µM of available **RGD** sites across all treatment groups (Supplementary Table 7). Coverslips across all groups were washed and incubated with either 33.3% of diluted hPL (Stemcell, 200-0361) or 200 ng ml^−1^ of recombinant growth factors, at 4 °C overnight, and thoroughly washed the next day. Coverslips were placed in well plates and incubated with serum-reduced ECGM MV2 media before seeding of cells.

### HMEC-1 bead migration on coverslips

Cell-coated microcarrier beads were seeded onto 10 mm control, scrTrAP-functionalized or TrAP-functionalized coverslips, at 0.8 mg ml^−1^, in 48-well plates and cultured in serum-reduced ECGM MV2 media. Bright-field images were acquired at 24, 48 and 144 h. Cell migration was quantified in MATLAB by manually measuring the distance between the migrated cells and their nearest bead of origin, using MathWorks Image Processing Toolbox ‘Measure Distances in an Image’.

### HDF coverslip and sponge assays

Primary HDFs used in in vitro assays were provided by C. Higgins (Department of Bioengineering, Imperial College London) and were sourced under the ethical approval detailed above for human skin. HDFs were cultured in low-glucose Dulbecco’s modified Eagle’s medium (DMEM) supplemented with 10% FBS, 100 U ml^−1^ of penicillin–streptomycin and 2 mM of GlutaMax. Before experiments, passage 3–6 HDFs were serum-starved overnight in serum-reduced DMEM media containing 0.1% FBS, 100 U ml^−1^ of penicillin–streptomycin and 2 mM of GlutaMax. Cells were trypsinized and resuspended in serum-reduced DMEM.

For proliferation assays on coverslips, HDFs were seeded at 10,000 cells cm^−2^. Images of cells adhered to the coverslips were captured at 48 h and 72 h using a Leica DM IL LED bright-field microscope. For end-point imaging, cells were fixed in 4% paraformaldehyde, stained for F-actin (ActinRed 555 ReadyProbes, 1 drop per millilitre) and nuclei (DAPI, 0.05 µg ml^−1^), washed and imaged using a Leica SP8 confocal microscope.

For cell proliferation on sponges, the sponges were rinsed twice in serum-reduced DMEM media, gently blotted dry on KimWipes and placed in 96-well plates. Then, 20,000 HDF cells were seeded at 1 million ml^−1^ into the centre of 4 or 5 mm collagen sponges (all sponges in the same experiment were of the same diameter). The cells were allowed to adhere onto the sponge for at least 3 h before serum-reduced DMEM media were added to the well plates. The media was changed every 3 days.

For the quantification of cell viability with PrestoBlue (Thermo Fisher, A13261), sponges were incubated for 2 h with 200 µl of 1:9 volumetric ratio of PrestoBlue to serum-reduced DMEM. After incubation, 150 µl of PrestoBlue solution was transferred to black cell culture microplates with µCLEAR bottom to minimize background fluorescence and cross-talk and measured with a Varioskan Flash microplate reader (Thermo Scientific) at 545 nm excitation/595 nm emission. Experimental repeats were conducted with different biological donors.

For live imaging, the cells were incubated with 5 µM CellTracker Green CMFDA dye for 45 min, washed once in DPBS and imaged using a Leica SP8 confocal microscope. Confocal *z*–stacks were converted to the maximum-intensity projections and cell numbers were manually quantified in FIJI.

### Quantifying HMEC-1 secretion of HGF

Passage 6 HMEC-1 cells were cultured at a density of 40,000 cells per well, in a 48-well plate, in fully supplemented ECGM MV 2 for 48 h. The growth medium was then removed and replaced with either fresh fully supplemented ECGM MV2 media with 5% FBS, serum-reduced ECGM MV 2 media (Basal Medium MV2, with 0.2% FBS, 1 µg ml^−1^ of ascorbic acid and 0.2 µg ml^−1^ of hydrocortisone), or endothelial-cell-free defined media (Cell Applications). To minimize any variability between groups, the media used for the control groups were also incubated in the wells without cells, and placed in cell culture conditions of 37 °C and 5% CO_2_. After 72 h, the incubation media were removed from each group and HGF ELISA (Cambridge Bioscience) was conducted on undiluted samples, according to the manufacturer’s instructions. Results were read using a microplate reader (Synergy HT) and the presence of HGF in the media was quantified against the standard curve, with values below the minimum detection limit of the kit (<3 pg ml^−1^) denoted as 0 pg ml^−1^.

### Ex vivo human skin wound model

Human abdominal skin was obtained from female donors aged 25–50 years undergoing abdominoplasty procedures at a private clinic, under informed written consent (see the ‘Ethics statement’ section). Skin samples were excluded from analysis if contamination was detected during culture, as assessed by visual inspection. The primary ex vivo wound-healing study (Fig. 5) was conducted using skin from three independent biological donors, with four to five wounds assessed per donor per treatment group; the exact sample sizes are indicated in the relevant figures and additional donors contributed to supplementary experiments used to optimize the experimental protocols before the main study (Supplementary Fig. 19).

Adapting methods from a previous protocol^65^, skin samples were first soaked in Antibiotic Antimycotic Solution (Thermo Fisher) at 2× concentration, followed by a 1× concentration, for at least 10 min each, before the adipose tissue was removed. An 8 mm biopsy punch (KAI Medical) was used to mark the outer circumference of the sample and a 3 mm biopsy punch was used to create a full-skin layer wound in the centre. An 8 mm × 8 mm section of the sample was then cut with a pair of surgical scissors. Furthermore, 12-well plates were prepared for the skin samples by placing 4 ply of non-woven swabs (Premier) into each well, followed by a layer of membrane filter paper (Millipore) that were cut into halves to fit the wells. Then, 1 ml of William’s E media (Thermo Fisher), supplemented with 10 µg ml^−1^ of insulin, 10 ng ml^−1^ of hydrocortisone, 100 U ml^−1^ of penicillin–streptomycin and 2 mM of GlutaMax, was added to each well so that the gauze and membrane paper were moist. The skin samples were then placed on top of the Nylon paper. Here 3 mm of hPL-loaded control and scrTrAP- and TrAP-functionalized sponges were equilibrated in William’s E media, gently blotted dry and placed into the 3 mm skin wound, such that the top of the sponge aligns with the top of the epidermal layer. The skin samples were incubated at 37 °C, 5% CO_2_ for 4 or 8 days. The media were replaced daily.

After 4 or 8 days, whole-mount skin sections were fixed in 4 ml of 4% PFA for 22 h and washed three times in PBS for at least 30 min each. The samples were then transferred to 48-well plates and stained with 300 µl of ActinRed 555 ready probes (Thermo Fisher, 1 drop per 3 ml) and DAPI (Life Technologies, 3.3 µg ml^−1^), overnight at 4 °C. Samples were washed with PBS the next day and imaged using a Leica SP8 inverted confocal microscope (Supplementary Table 8). Confocal *z*–stacks were converted into maximum-intensity projections in FIJI. The sponge channel was segmented using edge-based thresholding and the cell channels using intensity thresholding in MATLAB. The co-localized area between the two masks was quantified, as an indication of wound–sponge integration.

### Antibody staining of whole-mount skin

Selected whole-mount skin samples were immunostained for cytokeratin 14 (K14). Samples were blocked with 5% goat serum and 0.3% Tween-20 in PBS, incubated overnight with anti-K14 primary antibody (BioLegend, SIG-3476-100, 1:1,000), followed by Alexa Fluor 488 (Thermo Fisher, A-11039, 1:1,000). Samples were washed in PBS between staining steps and imaged using a Leica Stellaris 8 FALCON FILM microscope (Extended Data Fig. 2).

### Human skin histology

Skin samples were paraffin-embedded following dehydration through graded ethanol solutions and Histo-Clear, with paraffin infiltration performed overnight at 60 °C. Samples were embedded in paraffin and sectioned at 200 µm across the wound using a microtome. Sections were deparaffinized in an embedding cassette (Sigma-Aldrich), mounted in glycerol-based mounting medium (90% glycerol in PBS, 0.5% *N*-propyl gallate) and sealed with coverslips before imaging. The sections with the widest sponge cross-sectional area within the skin cross-sections were considered to be the closest to the wound centre and were selected for imaging and analysis. Tile-scan *z*-stack images were acquired using a Leica Stellaris 8 FALCON FILM microscope (Supplementary Table 9) and processed as maximum-intensity projections in FIJI. Individual fluorescence channels were exported and analysed in MATLAB. The sponge and dermis components were segmented from their respective fluorescence channels in MATLAB using edge-based and intensity thresholding, respectively. The co-localized area between the two masks was quantified as an indication of tissue–sponge integration.

### Ex vivo human skin pull-out test

To assess the mechanical robustness of the collagen sponge–skin interface during the wound-healing process, a pull-out test was developed (Extended Data Fig. 4). Here, 10 μl micropipette tips were threaded with a 0.4 mm-diameter steel needle and 0.2 mm-diameter cotton string, cut to fit vertically inside a 12-well plate and sterilized by soaking in 70% ethanol. Next, 1 mm-diameter through holes were created in 3 mm-diameter collagen sponges, with biopsy punches. The threaded 10 μl micropipette tips were coated with a thin layer of epoxy (Gorilla Epoxy), placed cleanly inside the 1 mm through hole of the scaffold (Extended Data Fig. 4) and allowed to cure for 24 h. The sponges were then functionalized with cysteine, scrTrAPs or TrAPs and inserted into 3 mm skin wounds.

After 5 and 8 days of cultivation, the skin wounds were subjected to uniaxial tensile pull-out testing using a Biomomentum Mach-1 mechanical testing system (model V500css), equipped with a 10 N load cell. Skin samples were placed on a flat surface and were held down by a 10 cm M6 steel washer, which rested on two layers of 1.5 mm adhesive tape, creating about the same total height as each skin depth (Extended Data Fig. 4). The thread was hooked onto a hook fixture, connected to the Biomomentum Mach-1 mechanical testing system and the sponges were pulled from the wound at a constant upward displacement rate of 0.2 mm s^−1^. The force–displacement data were recorded using Mach-1 Motion Software. The force–displacement data were smoothed and analysed in MATLAB. The maximum pull-out force and work done were extracted from each curve, with work done calculated as the area under the force–displacement curve using the trapezoidal method. Following mechanical testing, the samples were fixed in 4% paraformaldehyde overnight, stained and imaged.

### In vivo mouse wound-healing model

The study was conducted under University of Arkansas IACUC protocol 23034. As this was a cross-species confirmatory pilot study building on statistically significant findings from both the in vivo rat femoral defect model and the ex vivo human skin model described above, the sample sizes were kept at the IACUC-approved minimum levels for a pilot study in accordance with the 3Rs principles. Full-thickness 6 mm excisional wounds were created on the upper and lower dorsa of 12 week-old C57BL/6J mice (50/50 sex split), sourced from The Jackson Laboratory (Bar Harbor). Wound-level group sizes, with each mouse receiving two wounds, are reported in Fig. 6. Animals were randomly allocated to experimental groups before surgery.

For surgery, mice were anaesthetized with 2%–5% isoflurane for induction and 1%–3% during maintenance. Anaesthetic depth was confirmed by slowed rhythmic breathing, loss of palpebral reflex and loss of toe pinch reflex, and monitored throughout by respiratory rate and response to toe pinch. Mice received carprofen (5 mg kg^−1^, subcutaneously) for pain relief before wound excision and as needed post-operatively. Following the application of povidone–iodine antiseptic, control cysteine-, scrTrAP- or TrAP-functionalized collagen sponges were implanted immediately after wounding. TrAP and scrTrAP sponges were functionalized to capture FGF-2, PDGF-BB, VEGF-A and HGF from hPL. Wounds were covered using a modified 3M Tegaderm dressing, with a non-adherent Tegaderm interface layer positioned between the implanted sponge and the adherent outer Tegaderm layer to prevent dressing–sponge adhesion during healing. The Tegaderm-covered region was then secured with a secondary layer of 3M Transpore surgical tape.

The wound perimeter was traced onto acetate paper immediately post-wounding. Wound tracings were scanned and analysed in MATLAB, where images were binarized by intensity thresholding to isolate the wound boundaries and wound area quantified by pixel counting and converted to square millimetres. The wound diameter was estimated from the area on day 0 assuming a circular wound geometry and used to normalize the wound diameter measurements from histological cross-sections on days 3 and 10. On days 3 and 10 post-implantation, wounds were imaged in vivo using multiphoton microscopy under isoflurane anaesthesia, with the mouse positioned on a sterile heated stage maintained at 37 °C. Mice were euthanized in accordance with the approved IACUC protocol and the recommendations of the Panel on Euthanasia of the American Veterinary Medical Association. Full-thickness wounds and their surrounding skin were excised, flash frozen in optical cutting temperature compound at –80 °C, cryosectioned (20 μm) through the centre of the wound, stained with haematoxylin and eosin, and analysed for wound diameter and wound bed thickness. The unit of analysis was individual wounds; wounds in which the sponge had fallen off during the study were excluded from the analysis.

Animal well-being was monitored in accordance with the approved IACUC protocol. Animals were monitored continuously for the first 2 h following recovery from anaesthesia, then twice daily for the first three post-operative days and at least three times per week thereafter. Humane end-points included 20% body weight loss from baseline, or the presence of any of the following clinical signs: lethargy, ruffled haircoats, decreased motor activity, anorexia for greater than 24 h, substantial swelling at the surgical site, burrowing, hiding, head pressing or sensitivity to touch, at which point the animal would be immediately euthanized. In the event of wound infection, the wound would be cleansed under anaesthesia or the animal was euthanized based on the judgement of the principal investigator or attending veterinarian. No adverse events or animal losses were observed during the study.

### Statistics and reproducibility

The sample sizes were not generally predetermined by statistical methods, unless otherwise stated in Methods. For the rat femur study, the sample sizes were estimated by power calculation at the licence application stage; mouse skin sample sizes were based on IACUC-approved minimum levels for a pilot study. No data were excluded, except for the ex vivo skin samples due to contamination and in vivo mouse wounds due to collagen sponges falling off during the study. Statistical analyses were performed using GraphPad Prism software (v.9/11). The names of the statistical tests used and the number of data points and independent replicates are detailed in the figure captions. Significance is indicated in figures when *P* < 0.05.

### Reporting summary

Further information on research design is available in the Nature Portfolio Reporting Summary linked to this article.

## Online content

Any methods, additional references, Nature Portfolio reporting summaries, source data, extended data, supplementary information, acknowledgements, peer review information; details of author contributions and competing interests; and statements of data and code availability are available at 10.1038/s41563-026-02682-8.

## Supplementary information

Supplementary Information Supplementary Figs. 1–21, Tables 1–10, materials, methods and discussion.

Reporting Summary

## Source data

Source Data Fig. 1 Statistical source data.

Source Data Fig. 2 Statistical source data.

Source Data Fig. 3 Statistical source data.

Source Data Fig. 4 Statistical source data.

Source Data Fig. 5 Statistical source data.

Source Data Fig. 6 Statistical source data.

Source Data Extended Data Fig. 4 Statistical source data.

## Data Availability

The data supporting the findings of this study are available within the Article and its Supplementary Information. Source data are provided with this paper.

## Code availability

Custom MATLAB codes that were used in this Article to enable image registration for in vivo multiphoton imaging and for establishing the collagen sponge boundary within the in vivo wounds are available via Zenodo at 10.5281/zenodo.20707807^66^ and 10.5281/zenodo.20708392^67^, respectively.

## References

[1] Stejskalová, A. & Almquist, B. D. Using biomaterials to rewire the process of wound repair. *Biomater. Sci.* **5**, 1421–1434 (2017). 28692083 10.1039/c7bm00295e PMC5576529

[2] Cañedo-Dorantes, L. & Cañedo-Ayala, M. Skin acute wound healing: a comprehensive review. *Int. J. Inflam.* **2019**, 3706315 (2019). 31275545 10.1155/2019/3706315 PMC6582859

[3] Iversen, M. M. et al. History of foot ulcer increases mortality among individuals with diabetes: ten-year follow-up of the Nord-Trøndelag Health Study, Norway. *Diabetes Care* **32**, 2193–2199 (2009). 19729524 10.2337/dc09-0651 PMC2782976

[4] Brinker, M. R., Trivedi, A. & O’Connor, D. P. Debilitating effects of femoral nonunion on health-related quality of life. *J. Orthop. Trauma* **31**, e37–e42 (2017). 27755332 10.1097/BOT.0000000000000736

[5] Schade, A. T. et al. The economic burden of open tibia fractures: a systematic review. *Injury* **52**, 1251–1259 (2021). 33691946 10.1016/j.injury.2021.02.022

[6] Stewart, S. K., Tenenbaum, O., Higgins, C., Masouros, S. & Ramasamy, A. Fracture union rates across a century of war: a systematic review of the literature. *BMJ Mil. Health* **166**, 271–276 (2020). 32217686 10.1136/bmjmilitary-2019-001375

[7] Amirsadeghi, A. et al. Vascularization strategies for skin tissue engineering. *Biomater. Sci.* **8**, 4073–4094 (2020). 32539055 10.1039/d0bm00266f

[8] Phillips, C. J. et al. Estimating the costs associated with the management of patients with chronic wounds using linked routine data. *Int. Wound J.* **13**, 1193–1197 (2016). 25818405 10.1111/iwj.12443 PMC7949824

[9] Kerr, M. et al. The cost of diabetic foot ulcers and amputations to the National Health Service in England. *Diabet. Med.* **36**, 995–1002 (2019). 31004370 10.1111/dme.13973

[10] Park, J. W., Hwang, S. R. & Yoon, I. S. Advanced growth factor delivery systems in wound management and skin regeneration. *Molecules* **22**, 1259 (2017). 28749427 10.3390/molecules22081259 PMC6152378

[11] Edelman, E. R., Nugent, M. A. & Karnovsky, M. J. Perivascular and intravenous administration of basic fibroblast growth factor: vascular and solid organ deposition. *Proc. Natl Acad. Sci. USA* **90**, 1513–1517 (1993). 8434012 10.1073/pnas.90.4.1513 PMC45904

[12] Bowen-Pope, D., Malpass, T., Foster, D. & Ross, R. Platelet-derived growth factor in vivo: levels, activity, and rate of clearance. *Blood* **64**, 458–469 (1984). 6331547

[13] Yamakawa, S. & Hayashida, K. Advances in surgical applications of growth factors for wound healing. *Burns Trauma* **7**, s41038–019–0148–1 (2019). 10.1186/s41038-019-0148-1 PMC6450003 30993143

[14] James, A. W. et al. A review of the clinical side effects of bone morphogenetic protein-2. *Tissue Eng. Part B Rev.* **22**, 284–297 (2016). 26857241 10.1089/ten.teb.2015.0357 PMC4964756

[15] Epstein, N. Pros, cons, and costs of INFUSE in spinal surgery. *Surg. Neurol. Int.* **2**, 10 (2011). 21297932 10.4103/2152-7806.76147 PMC3031052

[16] Baldo, B. A. Side effects of cytokines approved for therapy. *Drug Saf.* **37**, 921–943 (2014). 25270293 10.1007/s40264-014-0226-z PMC7101846

[17] Papanas, N. & Maltezos, E. Benefit-risk assessment of becaplermin in the treatment of diabetic foot ulcers. *Drug Saf.* **33**, 455–461 (2010). 20486728 10.2165/11534570-000000000-00000

[18] Oliva, N. & Almquist, B. D. Spatiotemporal delivery of bioactive molecules for wound healing using stimuli-responsive biomaterials. *Adv. Drug Deliv. Rev.* **161–162**, 22–41 (2020). 32745497 10.1016/j.addr.2020.07.021

[19] Ooi, H. W., Hafeez, S., van Blitterswijk, C. A., Moroni, L. & Baker, M. B. Hydrogels that listen to cells: a review of cell-responsive strategies in biomaterial design for tissue regeneration. *Mater. Horiz.* **4**, 1020–1040 (2017).

[20] Kitano, H. Biological robustness. *Nat. Rev. Genet.* **5**, 826–837 (2004). 15520792 10.1038/nrg1471

[21] Csete, M. E. & Doyle, J. C. Reverse engineering of biological complexity. *Science* **295**, 1664–1669 (2002). 11872830 10.1126/science.1069981

[22] Cardelli, L., Csikász-Nagy, A., Dalchau, N., Tribastone, M. & Tschaikowski, M. Noise reduction in complex biological switches. *Sci. Rep.* **6**, 20214 (2016). 26853830 10.1038/srep20214 PMC4745012

[23] Chen, F. M., Zhang, M. & Wu, Z. F. Toward delivery of multiple growth factors in tissue engineering. *Biomaterials* **31**, 6279–6308 (2010). 20493521 10.1016/j.biomaterials.2010.04.053

[24] Chicharro-Alcántara, D. et al. Platelet rich plasma: new insights for cutaneous wound healing management. *J. Funct. Biomater.* **9**, 10 (2018). 29346333 10.3390/jfb9010010 PMC5872096

[25] dos Santos, R. G. et al. The regenerative mechanisms of platelet-rich plasma: a review. *Cytokine* **144**, 155560 (2021). 34004552 10.1016/j.cyto.2021.155560

[26] Qian, Z. et al. Improving chronic diabetic wound healing through an injectable and self-healing hydrogel with platelet-rich plasma release. *ACS Appl. Mater. Interfaces* **12**, 55659–55674 (2020). 33327053 10.1021/acsami.0c17142

[27] Stejskalová, A., Oliva, N., England, F. J. & Almquist, B. D. Biologically inspired, cell-selective release of aptamer-trapped growth factors by traction forces. *Adv. Mater.* **31**, 1806380 (2019). 10.1002/adma.201806380 PMC6375388 30614086

[28] Lei, K. & Tang, L. T cell force-responsive delivery of anticancer drugs using mesoporous silica microparticles. *Mater. Horiz.* **7**, 3196–3200 (2020).

[29] Velusamy, A., Sharma, R., Rashid, S. A., Ogasawara, H. & Salaita, K. DNA mechanocapsules for programmable piconewton responsive drug delivery. *Nat. Commun.* **15**, 704 (2024). 38267454 10.1038/s41467-023-44061-w PMC10808132

[30] Cassell, O. C. S., Hofer, S. O. P., Morrison, W. A. & Knight, K. R. Vascularisation of tissue-engineered grafts: the regulation of angiogenesis in reconstructive surgery and in disease states. *Br. J. Plast. Surg.* **55**, 603–610 (2002). 12550111 10.1054/bjps.2002.3950

[31] Kannan, R. Y., Salacinski, H. J., Sales, K., Butler, P. & Seifalian, A. M. The roles of tissue engineering and vascularisation in the development of micro-vascular networks: a review. *Biomaterials* **26**, 1857–1875 (2005). 15576160 10.1016/j.biomaterials.2004.07.006

[32] Shahin, H., Elmasry, M., Steinvall, I., Söberg, F. & El-Serafi, A. Vascularization is the next challenge for skin tissue engineering as a solution for burn management. *Burns Trauma* **8**, tkaa022 (2020). 32766342 10.1093/burnst/tkaa022 PMC7396265

[33] Rouwkema, J. & Khademhosseini, A. Vascularization and angiogenesis in tissue engineering: beyond creating static networks. *Trends Biotechnol.* **34**, 733–745 (2016). 27032730 10.1016/j.tibtech.2016.03.002

[34] Gerhardt, H. et al. VEGF guides angiogenic sprouting utilizing endothelial tip cell filopodia. *J. Cell Biol.* **161**, 1163–1177 (2003). 12810700 10.1083/jcb.200302047 PMC2172999

[35] Ubezio, B. et al. Synchronization of endothelial Dll4-notch dynamics switch blood vessels from branching to expansion. *eLife* **5**, e12167 (2016). 27074663 10.7554/eLife.12167 PMC4894757

[36] Bridges, E. M. & Harris, A. L. To branch or to expand?. *eLife* **5**, e17079 (2016). 27265819 10.7554/eLife.17079 PMC4894754

[37] Ozawa, C. R. et al. Microenvironmental VEGF concentration, not total dose, determines a threshold between normal and aberrant angiogenesis. *J. Clin. Invest.* **113**, 516–527 (2004). 14966561 10.1172/JCI18420 PMC338257

[38] Nakatsu, M. N. et al. VEGF121 and VEGF165 regulate blood vessel diameter through vascular endothelial growth factor receptor 2 in an in vitro angiogenesis model. *Lab. Invest.* **83**, 1873–1885 (2003). 14691306 10.1097/01.lab.0000107160.81875.33

[39] von Degenfeld, G. et al. Microenvironmental VEGF distribution is critical for stable and functional vessel growth in ischemia. *FASEB J.* **20**, 2657–2659 (2006). 17095533 10.1096/fj.06-6568fje

[40] Blanco, R. & Gerhardt, H. VEGF and notch in tip and stalk cell selection. *Cold Spring Harb. Perspect. Med.* **3**, a006569 (2013). 23085847 10.1101/cshperspect.a006569 PMC3530037

[41] Parsons-Wingerter, P. et al. A VEGF165-induced phenotypic switch from increased vessel density to increased vessel diameter and increased endothelial NOS activity. *Microvasc. Res.* **72**, 91–100 (2006). 16872639 10.1016/j.mvr.2006.05.008

[42] LeBrasseur, N. Two ways VEGF-A builds a vessel. *J. Cell Biol.* **161**, 1006 (2003).

[43] Pufe, T. et al. Quantitative measurement of the splice variants 120 and 164 of the angiogenic peptide vascular endothelial growth factor in the time flow of fracture healing: a study in the rat. *Cell Tissue Res.* **309**, 387–392 (2002). 12195295 10.1007/s00441-002-0605-0

[44] Sadeghifar, A. et al. The effect of waterpipe tobacco smoking on bone healing following femoral fractures in male rats. *Front. Surg.* **8**, 722446 (2021). 34671637 10.3389/fsurg.2021.722446 PMC8520932

[45] Nanobashvili, J. et al. Comparison of angiogenic potential of human microvascular endothelial cells and human umbilical vein endothelial cells. *Eur. Surg.* **35**, 214–219 (2003).

[46] Makino, T. et al. Basic fibroblast growth factor stimulates the proliferation of human dermal fibroblasts via the ERK1/2 and JNK pathways. *Br. J. Dermatol.* **162**, 717–723 (2010). 19995368 10.1111/j.1365-2133.2009.09581.x

[47] Barrientos, S., Brem, H., Stojadinovic, O. & Tomic-Canic, M. Clinical application of growth factors and cytokines in wound healing. *Wound Repair Regen.* **22**, 569–578 (2014). 24942811 10.1111/wrr.12205 PMC4812574

[48] Wang, Y. et al. Regulation of VEGF-induced endothelial cell migration by mitochondrial reactive oxygen species. *Am. J. Physiol. Cell Physiol.* **301**, C695–C704 (2011). 21653897 10.1152/ajpcell.00322.2010 PMC3174570

[49] Birchmeier, C., Birchmeier, W., Gherardi, E. & Vande Woude, G. F. Met, metastasis, motility and more. *Nat. Rev. Mol. Cell Biol.* **4**, 915–925 (2003). 14685170 10.1038/nrm1261

[50] Georgakoudi, I. & Quinn, K. P. Label-free optical metabolic imaging in cells and tissues. *Annu. Rev. Biomed. Eng.* **25**, 413–443 (2023). 37104650 10.1146/annurev-bioeng-071516-044730 PMC10733979

[51] Zhang, H., Vandesompele, J., Braeckmans, K., De Smedt, S. C. & Remaut, K. Nucleic acid degradation as barrier to gene delivery: a guide to understand and overcome nuclease activity. *Chem. Soc. Rev.* **53**, 317–360 (2024). 38073448 10.1039/d3cs00194f

[52] Ni, S. et al. Chemical modifications of nucleic acid aptamers for therapeutic purposes. *Int. J. Mol. Sci.* **18**, 1683 (2017). 28767098 10.3390/ijms18081683 PMC5578073

[53] Schultz, G. S. & Wysocki, A. Interactions between extracellular matrix and growth factors in wound healing. *Wound Repair Regen.* **17**, 153–162 (2009). 19320882 10.1111/j.1524-475X.2009.00466.x

[54] Wu, D. et al. Bioinstructive scaffolds enhance stem cell engraftment for functional tissue regeneration. *Nat. Mater.* **24**, 1364–1374 (2025). 40247020 10.1038/s41563-025-02212-y PMC12777987

[55] Mochizuki, M. et al. Growth factors with enhanced syndecan binding generate tonic signalling and promote tissue healing. *Nat. Biomed. Eng.* **4**, 463–475 (2020). 31685999 10.1038/s41551-019-0469-1

[56] Martino, M. M. et al. Growth factors engineered for super-affinity to the extracellular matrix enhance tissue healing. *Science* **343**, 885–888 (2014). 24558160 10.1126/science.1247663

[57] US Food and Drug Administration. *Potency Assurance for Cellular and Gene Therapy Products: Draft Guidance for Industry* https://www.fda.gov/regulatory-information/search-fda-guidance-documents/potency-assurance-cellular-and-gene-therapy-products (US Food and Drug Administration, 2023).

[58] European Medicines Agency. *Guideline on Human Cell-Based Medicinal Products*. Report No. EMEA/CHMP/410869/2006 (European Medicines Agency, 2008).

[59] European Medicines Agency. *Guideline on Potency Testing of Cell Based Immunotherapy Medicinal Products for the Treatment of Cancer*. Report No. EMA/CHMP/BWP/271475/2006 Rev. 1 (European Medicines Agency, 2016).

[60] Salmikangas, P., Carlsson, B., Klumb, C., Reimer, T. & Thirstrup, S. Potency testing of cell and gene therapy products. *Front. Med.* **10**, 1190016 (2023). 10.3389/fmed.2023.1190016 PMC10196484 37215709

[61] Ferreira, S. A. et al. Bi-directional cell-pericellular matrix interactions direct stem cell fate. *Nat. Commun.* **9**, 4049 (2018). 30282987 10.1038/s41467-018-06183-4 PMC6170409

[62] Loebel, C., Mauck, R. L. & Burdick, J. A. Local nascent protein deposition and remodelling guide mesenchymal stromal cell mechanosensing and fate in three-dimensional hydrogels. *Nat. Mater.* **18**, 883–891 (2019). 30886401 10.1038/s41563-019-0307-6 PMC6650309

[63] Bruno, J. G. Long shelf life of a lyophilized DNA aptamer beacon assay. *J. Fluoresc.* **27**, 439–441 (2017). 28039562 10.1007/s10895-016-2014-x

[64] Kedzierski, S., Khoshnejad, M. & Caltagirone, G. T. Synthetic antibodies: the emerging field of aptamers. *Bioprocess. J.* **11**, 46–49 (2012).

[65] Topouzi, H., Boyle, C. J., Williams, G. & Higgins, C. A. Harnessing the secretome of hair follicle fibroblasts to accelerate ex vivo healing of human skin wounds. *J. Invest. Dermatol.* **140**, 1075–1084.e11 (2019). 31682842 10.1016/j.jid.2019.09.019

[66] Quantitative Optical Imaging Lab. kylepquinn/3D-MPM-registration: v1.0 - Initial Release - code related to Jones et al., Communications Biology, 2018. *Zenodo*10.5281/zenodo.20707807 (2026).

[67] Quantitative Optical Imaging Lab. kylepquinn/autofluorescence-infiltration-nature-materials: v1.0 - Initial Release. *Zenodo*10.5281/zenodo.20708392 (2026).

