# Supplementary material for: Force-responsive biomaterials drive tissue repair by harnessing endogenous growth factors
Source: Nat Mater. 2026 Jul 27;25(9):1646–57. doi: 10.1038/s41563-026-02682-8 (PMC13518237; doi:10.1038/s41563-026-02682-8)
Supplement: Supplementary file 1 [file 41563_2026_2682_MOESM1_ESM.pdf]

nature materials

Supplementary information

https://doi.org/10.1038/s41563-026-02682-8

Force-responsive biomaterials drive tissue repair by harnessing endogenous growth factors

In the format provided by the authors and unedited

Materials ... 2
Aptamer Details for TrAPs Used ... 3
In Vitro Tube Formation Assay with Endothelial Cells ... 3
Tube Formation Assay – Supplementary Methods ... 3
Tube Formation Assay – Preliminary Results ... 5
In Vitro Bead Sprouting Assay with Endothelial Cells ... 7
Sprouting Assay – Supplementary Methods ... 7
Sprouting Assay Characterisation and Analysis – Supplementary Methods ... 8
Sprouting Assay – Supplementary Notes ... 8
Modulation of Angiogenesis In Vivo During Rat Femur Defect Repair ... 10
Bone Growth – Results ... 10
Animal Well-Being – Results ... 10
Harvesting of Endogenous VEGF-A and HGF from Cells ... 11
Empty VEGF-TrAPs - Supporting Results ... 11
HMEC-1 Response to Recombinant HGF ... 12
HMEC-1 Response to HGF – Supporting Results ... 12
HMEC-1 Response to HGF-TrAPs – Supporting Results ... 13
Human Dermal Fibroblast Response to Recombinant FGF-2-TrAPs ... 15
HDF Dose-Response to FGF-2 – Supporting Results ... 15
HDF Response to FGF-2-TrAPs – Supporting Results ... 15
Quantification of Growth Factors in Blood Lysate ... 17
Implications for Maximum TrAP Loading – Supplementary Notes ... 17
2D Proliferation and Migration Assays ... 19
HDF response to HPL-loaded PDGF-BB-TrAPs – Biological Repeats ... 19
Combinatorial TrAPs – Supplementary Results ... 21
FGF-2 and PDGF-BB-TrAPs Collagen Sponge In Vitro Evaluation – Preliminary Study ... 21
FGF-2, PDGF-BB and HGF-TrAPs Collagen Sponges In Vitro Evaluation – Supporting study ... 23
FGF-2, PDGF-BB and HGF-TrAPs in Ex Vivo Human Skin – Preliminary study ... 24
Ex Vivo Human Skin Model – Method Notes ... 25
Tensile Pull-Out Test of Sponge-Skin Mechanical Interface ... 26
Supplementary Discussion ... 26
In Vivo Imaging of Mouse Skin Wound Healing ... 26
Calculations and Comparison of Growth Factors Used per Volume ... 28

Materials

Human microvascular endothelial cells (HMEC-1, ATCC, CRL-3243), Endothelial Cell Growth Medium MV (Promocell,C-22020), Endothelial Cell Growth Medium MV2 (Promocell, C-22121), Collagen I (Roche, 11179179001), Acetic Acid 2%(VWR,86139.310), Human Recombinant VEGF-A165 (Biolegend, 583706), Immobilized TCEP Disulfide Reducing Gel (Thermo Fisher, 77712), borate buffer (Thermo Fisher, BupH™ Borate Buffer Packs, 28384, pH 8.5), maleimide-PEG8 succinimidyl ester (Sigma, 746207), maleimide-PEG2 succinimidyl ester (Sigma, 746223), CellTracker™ Green CMFDA Dye (Thermo Fisher, C2925), GlutaMAX™ Supplement (Thermo Fisher, 35050061), Penicillin-Streptomycin (Pen/Strep, Sigma, P4333). Phosphoramidites compatible with UltraFAST deprotection (Bz-dA-CE, Ac-dC-CE, dmf dG-CE, and dT-CE, LINK), 3'-Thiol-Modifier C3 S-S CPG (LINK, 2361), 5'-Bromohexyl Phosphoramidite (Glen Research, 10-1946), spacer-CE Phosphoramidite 18 (HEG, LINK, 2129-F100X1), sodium azide (Sigma, S2002), N,N-Dimethylformamide (DMF, Sigma, D4551), ammonium hydroxide (Sigma, 16748-250ML-F), methylamine (Sigma, 426466), Dibenzocyclooctyne-maleimide (Sigma, 760668, MW= 427.45), GRGDSPC (Genscript, 98% purity, guaranteed TFA removal), GRDGSPC (Genscript, 98% purity, guaranteed TFA removal), Collagen sponges (Avitene Ultrafoam Collagen, 1050050, 8.5cm x 12.5 cm x 3mm), 1-(2 Aminoethyl)maleimide hydrochloride (Sigma, 56951, MW 254.16), N-Ethyl-N'-(3 dimethylaminopropyl)carbodiimide hydrochloride (EDC, Sigma, 03449), N Hydroxysulfosuccinimide sodium salt (NHS, Sigma, 56485), Dulbecco's Modified Eagle Medium (DMEM, low glucose, Sigma, D5546), PrestoBlue Cell Viability Reagent (Thermo Fisher, A13261), Cytodex 3 microcarriers (Cytiva, 17-0485-01), Texas Red-X, succinimidyl ester, mixed isomers (Thermo Fisher, T6134), µCLEAR® bottom were used in all experiments (Greiner, 655986), O.C.T. compound, mounting media (VWR, 361603E), Human Recombinant VEGF-A121(StemCell Technologies, 78127.1), Falcon® 5 mL Round Bottom Polystyrene Test Tube (Corning, 352235), 60mm x 15 mm Cell culture dish (Corning, 3295), 35mm x 10mm Cell Culture dish (Corning, 430165), Endothelial Cell Serum-Free Defined Medium without Phenol Red (Cell Applications, 113PR-500), 10mm circular coverslips (Agar Scientific, AGL46R10-1), PDGF-BB DuoSet ELISA (R&D Systems, DY220), DuoSet ELISA Ancillary Reagent Kit 2 (R&D Systems, DY008), Human FGF2 DuoSet ELISA (R&D Systems, DY233), Human VEGF DuoSet ELISA (R&D Systems, DY293B), Human HGF ELISA(For Lysates, Cambridge Bioscience, ELH-HGF-CL-1), XF human platelet lysate (Stemcell technologies, 200-0361), Recombinant human FGF-2 protein (aa155, Gibco, PHG0266), ActinRed™555ReadyProbes(Thermofisher,R37112), 5mm circular coverslips (Agar Scientific, AGL46R5-1), Recombinant Human PDGF-BB (BioLegend, 577302), Biopsy Punch KAI Disposable 4mm (Selles Medical, BP-40F), Biopsy Punch KAI Disposable 3mm(Selles Medical, BP-30F), Bovine Serum Albumin (Sigma Aldrich, A1470), Antimycotic Solution (ABAM, Thermo Fisher,15240062), 8mm biopsy punch (KAI Medical, BP-80F), Non-woven swabs (Premier,1860), membrane filter paper (Millipore, HNWP02500), William's E media (Thermo Fisher, 22551022), Insulin(Sigma Aldrich, I9278-5ML), Hydrocortisone (Sigma Aldrich,H4001-1G), embedding cassette (Sigma Aldrich, H0542-1CS), Paraformaldehyde (Sigma, 158127-100G), Propyl gallate, 98% (Thermofisher, 131581000), Epredia™ MX35 Premier™ Disposable Low-profile Microtome Blades (Fisher Scientific, 10687875), Goat anti-Chicken IgY (H+L) Secondary Antibody, Alexa Fluor™ 488 (Thermo Fisher, A-11039), Keratin 14 Polyclonal Chicken Antibody (BioLegend, SIG-3476-100), Anti-CD-31 (Abcam, ab182981), Anti-a-SMA (Abcam, ab7817), Goat anti-rabbit (Abcam, ab97049), goat anti-mouse (ab6788), Anti-vWF (Thermo Fisher, MAS-14029), streptavidin-HRP solution (Abcam, ab64269), Pierce™ 16% Formaldehyde (w/v) Methanol-free (Thermo Fisher, 28906), Ampule Breakers (Thermo Fisher, 28916), Hoechst 33342 (Thermo Fisher, 6224), Actin Cytoskeleton/Focal Adhesion Staining kit (Sigma, FAK100), DAPI (4', 6-diamidino-2 phenylindole, dihydrochloride, Life Technologies, 2116140), sulfuric acid (Fisher Scientific, S/9240/PB17), hydrogen peroxide 30% (VWR, 23615.261),

tweezer kit (Agar Scientific, AGT5530), Coupling buffer (CB, 50 mM sodium phosphate pH 7.2 at 4°C, 50 mM NaCl, 10mM EDTA), Dulbecco's Phosphate Buffered Saline (DPBS, Sigma Aldrich, D8537), PBS-Phosphate-Buffered Saline (10X) pH 7.4, (Thermo Fisher, AM9625)

Aptamer Details for TrAPs Used

Aptamers used for TrAPs	Modified Sequence (5'-3')	Original Reference	K_d of original aptamer from ref (nM)
VEGF-A Aptamer	TTA GGG CCA CGT CTA TTT AGA CTA GAG TGC AGT GGT TC TTTTT	[1]	0.2
PDGF-BB Aptamer	TTTTTCAGGCTACGGCACGTAGAGCATCACCATGATCC TGTTTTT	[2]	0.1
FGF-2 Aptamer	GGT GCT AAC CAG GAC ACA CC	[3]	3
HGF Aptamer	TTGCGCCAGCTTTGCTGATGGGTGGCCACCCTTGCCCT GGGTTTGAATTTCGATCCTATCGTTT	[4]	20
Random ssDNA	GATCCAAGTAGCGTTTGACGTATGGCTATAGCATGGC TCGTTT	-	-

Supplementary Table 1: Aptamers used for the synthesis of TrAPs. Aptamer sequences, their original reference, and the associated binding constant from the reference.

In Vitro Tube Formation Assay with Endothelial Cells

Tube Formation Assay - Supplementary Methods

To obtain 3.85 mg/ml of functionalised collagen, the dissolved collagen solution was mixed on ice with basal media, 1 M NaOH and 50 mM maleimide-PEG8-succinimidyl ester, as described in Supplementary Table 2. Coating the pipette tip with 0.2% acetic acid would help reduce gel adhesion to the pipette tip. The solution was gently mixed through pipetting and gentle tapping of the microcentrifuge tube, while avoiding the formation of bubbles. 15 µl (70 µl) of gel solution was added to each well of a 384 well-plate (96 well-plate), with tapping of the well-plate against a table surface to ensure even spreading of the gel. The gels were then incubated for 10mins (20mins) at room temperature before being incubated for 40 minutes at 37°C with 5% CO_2. After which, the gels were gently washed 5 times with coupling buffer (50 mM sodium phosphate pH 7.2, 50 mM NaCl, 10 mM EDTA), with each wash lasting at least 3 minutes to allow sufficient diffusion of solution through the gel. 20 µl (100 µl) of 1 µM reduced TrAPs/scrTrAPs/Cysteine was added to each well and incubated for at least 3 hours at room temperature. The gels were then washed 7 times with DPBS to remove any unbonded TrAPs/scrTrAPs/Cysteine, with each wash lasting at least 3 minutes. 20 µl (100 µl) of 200 ng/ml VEGF-A_{165} was then added to each well of gel and incubated for 3 hours at 4°C. The gels were washed twice with DPBS and thrice with ECGM Basal MV 2 Media. Trypsinised, serum starved HMEC-1 cells were seeded at a cell density between 140,000 – 176,000 cells/cm^2 in media compositions as shown in Supplementary Table 3. Media compositions and cell densities were varied between each

set of experiments but within experiment set, all sample groups - TrAPs, scrTrAPs and Cysteine - were prepared under the same conditions.

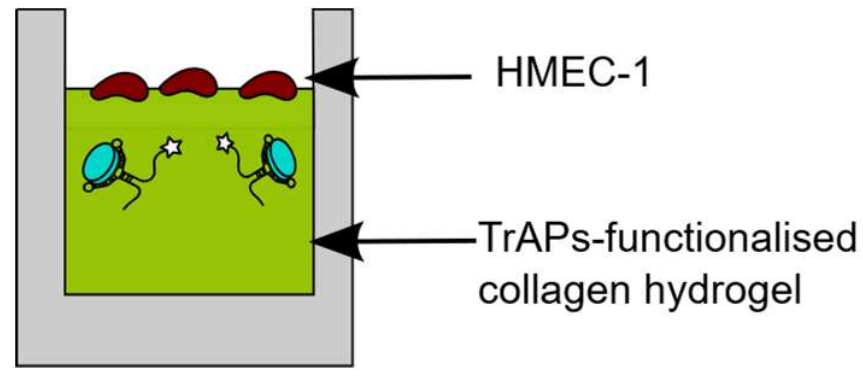

Supplementary Figure 1: Schematic of TrAP-functionalised collagen gel experiment in 96 or 384 well-plates. Collagen gels were functionalised with TrAPs. HMEC-1 cells were subsequently seeded on the surface to facilitate endothelial tube formation.

Reagents	(%)
7.69 mg/ml collagen (Roche, 11179179001)	50
Basal ECGM MV 2 Media (Promocell, C-22221)	47.8
1 M NaOH	1.6
50 mM maleimide-PEG8-succinimidyl (Sigma, 746207)	0.6

Supplementary Table 2: Percentage ratio of reagents to obtain functionalised 3.85 mg/ml of collagen solution.

Variations	Media Compositions
1	low glucose DMEM, 0.1% FBS, 10 mM L-Glutamine, 100 U/ml penicillin-streptomycin
2	1:250 dilution of ECGM MV Growth Medium (Promocell, C-22020) with Basal ECGM MV Media
3	Basal ECGM MV Media (Promocell, C-22220)

Supplementary Table 3: Media Compositions used for the tube formation media.

Cells were incubated with 2 µM of Celltracker Green CMFDA for 45 minutes, either prior to seeding onto collagen hydrogels, or 20 hours post-seeding. The Celltracker Green CMFDA solution was subsequently replaced with fresh media. The tube formation assays were imaged using a Leica SP8 Confocal Microscope. Confocal Z stack images were converted into maximum-intensity projections in FIJI and adjusted using manual thresholding prior to analysis in MATLAB. Images were segmented using adaptive local thresholding to obtain the vascular area of each discontinuous object (ie. Vascular networks), as shown in Supplementary Figure 2.

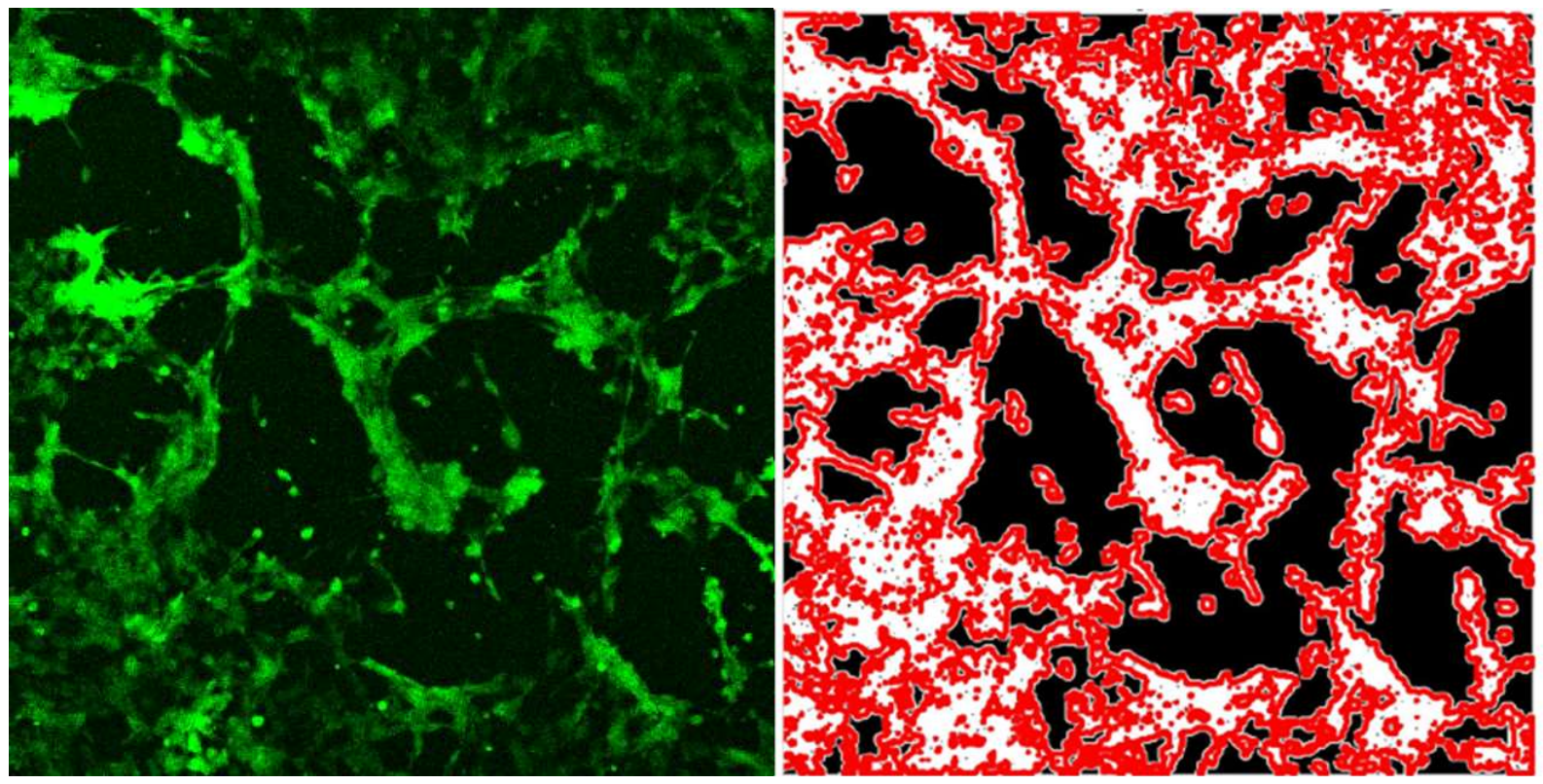

Supplementary Figure 2: Identification of endothelial network structures for image analysis. Fluorescence images of HMEC-1 cells (CellTracker Green CMFDA: green) cultured on a VEGF-TrAPs functionalised collagen gel, 24 hours post seeding (left). Corresponding thresholded and segmented image used for analysis (right), in which disconnected network structures were identified for object-based area measurements.

Tube Formation Assay - Preliminary Results

To test the robustness of the vascularisation effects observed with VEGF-TrAPs, the study was repeated in various environmental conditions, including varying cell density and media composition. In each set of experimental conditions, VEGF-TrAPs promoted larger vascular networks compared to the scrTrAPs and the cysteine control groups at 24 hours (Supplementary Figure 3a,c). Additionally, while the decrease in the number of discontinued networks in the VEGF-TrAPs group compared to the scrTrAPs and control groups was not statistically significant, it suggests that VEGF-TrAPs may facilitate larger vascular networks by increasing the interconnectivity of HMEC-1 cells (Supplementary Figure 3b,c). After 48 hours however, the difference in average vascular area between treatment groups decreased as vascular networks started to break down. This outcome was expected, given that the experiment was conducted in serum-reduced minimal media, which is insufficient to sustain stable vessel formation beyond 48 hours. Nonetheless, the increased vascularisation observed at 24 hours post-seeding, suggests promising potential of VEGF-TrAPs to promote vascularisation, providing reasons for further investigation of their pro-angiogenic capabilities.

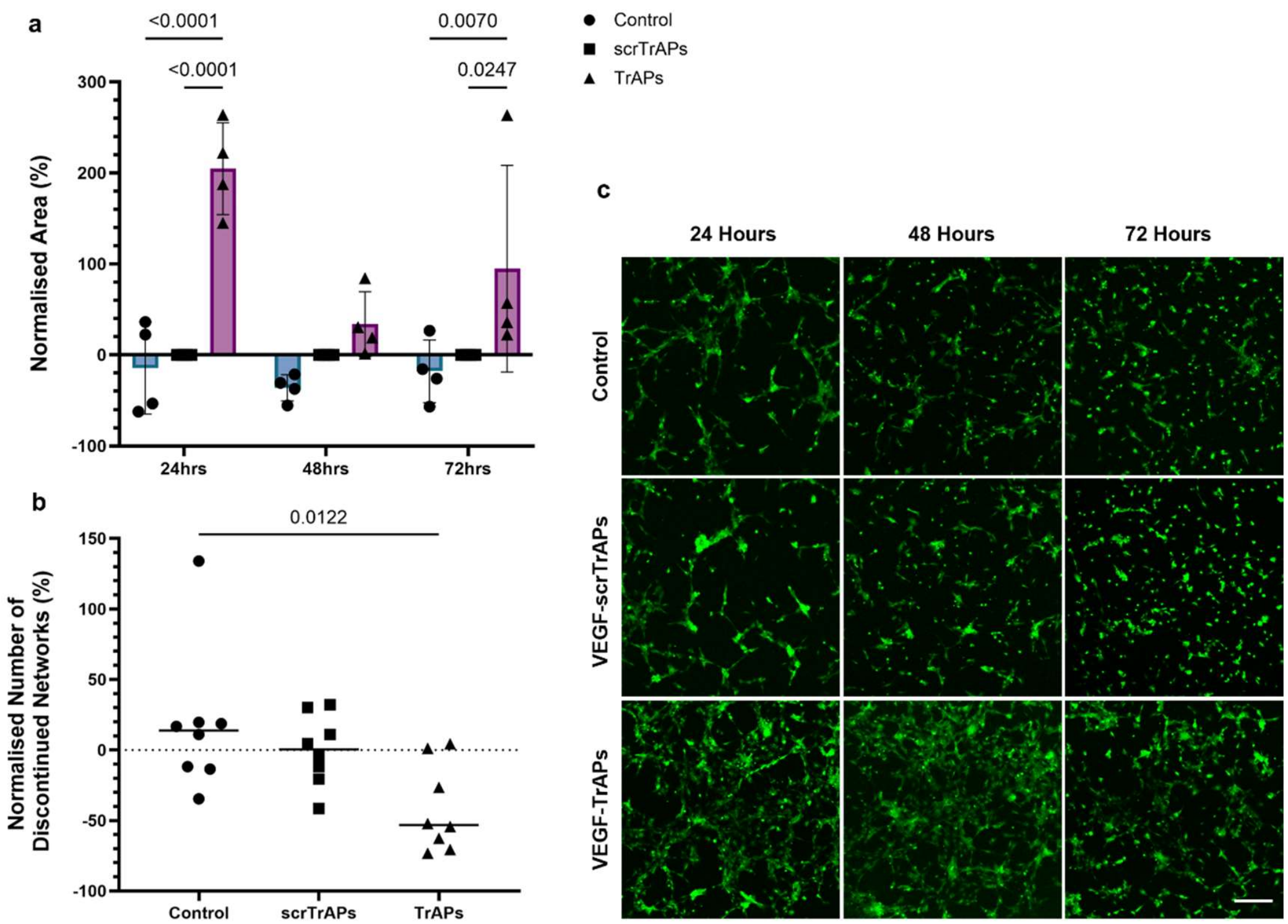

Supplementary Figure 3: Endothelial network formation on VEGF-TrAP-functionalised collagen hydrogels. a, Quantification of HMEC-1 vascular network area across different HMEC-1 cell density and media composition. The vascular area was normalised to the mean vascular area of the scrTrAPs group within each independent experiment. **b,** Quantification of disconnected network structures or objects at 24 hours post-seeding. The number of discontinued networks were normalised to the VEGF-scrTrAPs group within each independent experiment and time-point. **c,** Maximum-intensity projection confocal images of HMEC-1 networks in tube formation assays at 24, 48 and 72 hours (Celltracker green CMFDA: green, scale bar: 200 µm). For **a,** n = 4 independent experiments; each data point represents the mean vascular network area calculated from at least 4 hydrogel samples per group within an experiment. Statistical analysis was conducted using two-way ANOVA, followed by Tukey post-hoc test. Data presented as mean ± SD. For **b,** n = 8 hydrogel samples across 2 independent experiments. Statistical analysis was conducted using one-way ANOVA, followed by Tukey post hoc test. Data presented as individual data points; horizontal lines indicate median.

In Vitro Bead Sprouting Assay with Endothelial Cells

Sprouting Assay – Supplementary Methods

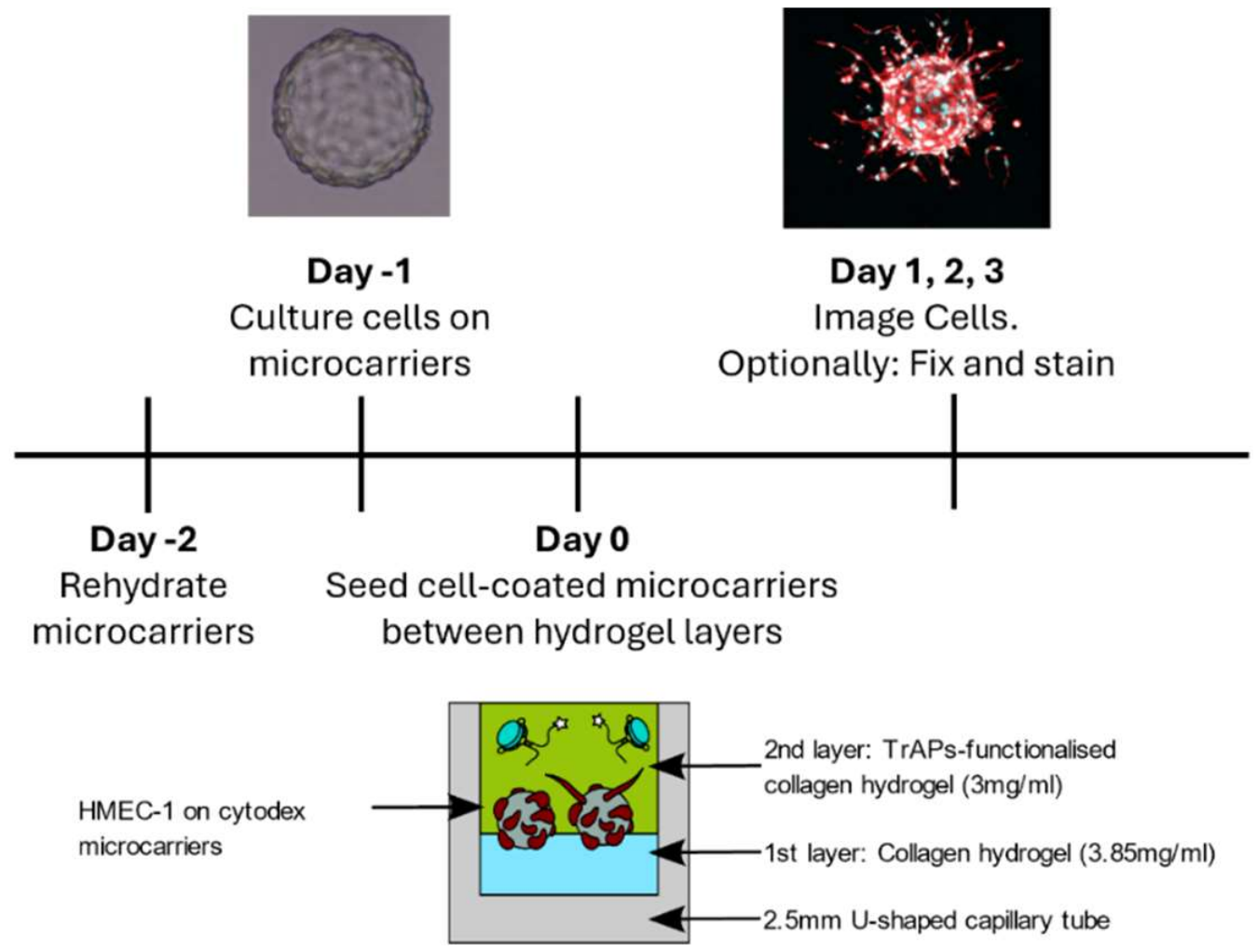

Supplementary Figure 4: Workflow of the angiogenic sprouting assay. Carrier beads were deposited on a bottom layer of unfunctionalised collagen, followed by the addition of TrAP-functionalised collagen on top.

	Reagents	% of total gel volume
1st layer (3.85 mg/ml)	5.71 mg/ml collagen in 0.2% Acetic acid (Roche, 11179179001)	67.41
	Basal MV 2 Media (Promocell, C-22221)	30.51
	1 M NaOH	2.08
2nd layer (3 mg/ml)	5.71 mg/ml collagen	52.5
	500 µM maleimide-PEG2- succinimidyl ester	2.0 (10 µM in final solution)
	8uM TrAPs/ scrTrAPs/ Cysteine	12.5 (1 µM in final solution)
	Starvation ECGM MV2 Media (0.2% FBS, 100 U/ml penicillin-streptomycin, 2 mM GlutaMax)	31.4
	1 M NaOH	1.6

Supplementary Table 4: Percentage ratio of reagents to collagen solution for the 1st and 2nd layer of collagen gel. The collagen solution was prepared on ice and mixed by gently pipetting and tapping of the Eppendorf tube, while avoiding any air bubbles, before transferring to the U-shaped capillary tube.

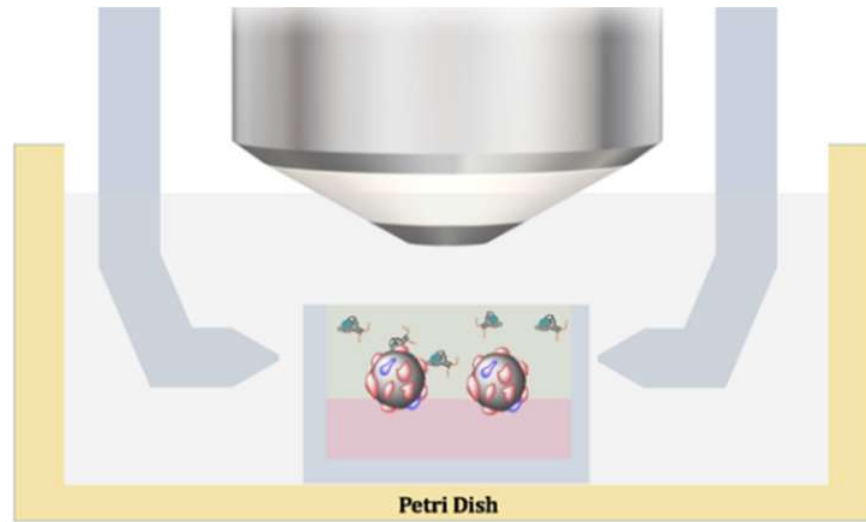

Supplementary Figure 5: Light-sheet imaging of endothelial sprouts within capillary tubes. Illustration of the light sheet microscope set-up used for three-dimensional imaging of endothelial sprouts along the capillary tube.

Sprouting Assay Characterisation and Analysis – Supplementary Methods

Sprout lengths of both primary and secondary sprouts were manually quantified using MATLAB where primary sprouts were defined as distance from the tip of longest connected sprout to surface of the bead, while secondary sprouts were defined as the distance from the tip of branching sprout/protrusions to the body of the primary sprout. Since filopodia protrusions at tip cells are early initiation of branching/sprouting induced by the binding of VEGF-A to VEGFR2[5], these filopodia-like protrusions were also quantified as sprouts to capture the effect of cell-activated VEGF-A release overtime, that could lead to varying stages of angiogenesis at the captured timepoint. For the quantification of number of sprouts, both primary and secondary sprouts were included (Supplementary Figure 6). Sprout thickness was measured at the widest portion of each sprout (Supplementary Figure 6).

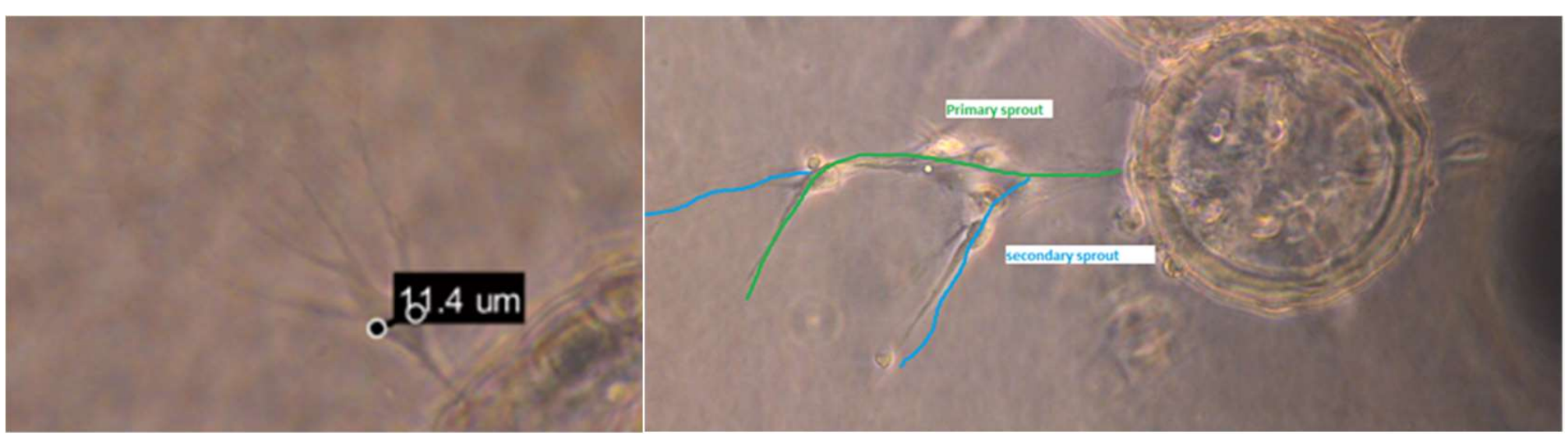

Supplementary Figure 6: Identification of endothelial sprouts in an in vitro angiogenic sprouting assay. Images showing identification of primary sprout (green), defined as the longest continuous sprout from the bead, and secondary sprouts (blue), defined as sprouts or protrusions extending from a primary sprout.

Sprouting Assay – Supplementary Notes

It is important to note that cells are highly sensitive to different substrate characteristics, which can alter membrane tension, actin polymerisation dynamics, focal adhesion maturation, and cytoskeletal organisation, thereby altering traction force generation[6,7]. The curvature of surfaces is one well-established regulator of focal adhesion characteristics; cells on convex surfaces form numerous but short-lived focal adhesions and lower intracellular tension, while cells on concave surfaces develop fewer but more stable focal adhesions with higher intracellular tension, which is mediated by actomyosin contractility and nucleo-cytoskeletal coupling[8]. The magnitude of this

curvature-dependent response is logarithmically related to the radius of curvature, with the strongest effects observed when the radius is similar to or smaller than the cell body (approximately 10-50 µm), and weaker effects at larger radii [8–10].

In the in vitro bead sprouting assays, Cytodex 3 microcarrier beads had manufacturing variability in diameter (131-215 µm; radii of 65-107 µm), placing them in a size regime where curvature-dependent differences in focal adhesion tension are expected to be modest compared to cell-scale curvatures[8]. Cells adhering to these beads encounter a convex geometry, which may result in focal adhesions that are less tensed and shorter-lived than those formed on flat or concave substrates. However, the data show that cells are still able to activate TrAPs. TrAP unfolding requires only that cells exceed a threshold force sufficient to mechanically unfold the aptamer construct, which based on the data, is achieved. This threshold is expected to be in a range similar to the force required to unfold aptamers using optical tweezers, which have found the unfolding of both RNA-based riboswitch aptamers[11] and DNA aptamers[12]to occur in the piconewton range, a force achievable by even nascent, immature adhesive structures.

Filopodial integrin bonds, which precede the formation of mature focal adhesions and are less tensed than stable focal adhesions, have been shown to exert forces in the range of tens of piconewtons[13]. Notably, short DNA duplexes with an unzipping force of approximately 10 pN for dwell times of seconds have been shown to unzip under filopodial integrin tension alone[13]. This demonstrates that even the least mature adhesive contacts can mechanically unfold DNA constructs under physiologically relevant loading conditions. Furthermore, convex geometries can promote filopodial activity: dynamic introduction of convex curvature reduces membrane tension[14], which is a facilitator of filopodia formation[15]. This means that the bead geometry could favour a type of nascent adhesive contact that can trigger TrAP activation, although given the curvature to cell size difference, enhanced filopodia formation is not predicted to be significant. Since TrAPs are nucleic acid constructs in a folded conformation, these studies do suggest that even the less-tensed, nascent adhesive contacts characteristic of cells exploring their surroundings are likely sufficient to trigger TrAP unfolding, without requiring the higher forces associated with mature, stable focal adhesions.

Nonetheless, the primary consideration here is not whether curvature affects the absolute magnitude of traction forces on individual beads, but whether bead-size variability results in a systematic difference in TrAP activation between experimental groups due to reducing the magnitude of traction forces below the required activation threshold. This scenario is unlikely for the following reasons:

First, beads from the same batch were used across experiments and randomly allocated to TrAP, scrTrAP, and control groups within each experiment. As a result, any potential modest curvature-dependent impact on focal adhesion tension and filopodia generation should be equally distributed across all groups and would not produce a systematic difference between TrAPs and scrTrAPs.

Second, if curvature were introducing a systematic mechanobiological bias, the effect size would be expected to vary with the bead size distribution across experiments.[8] However, the consistent results observed within groups for beads of different sizes across four independent experiments, each using bead populations prepared independently and with distinct size distributions and different cell passages, does not provide evidence that this systematic mechanobiological bias is present at a biologically significant scale.

Modulation of Angiogenesis In Vivo During Rat Femur Defect Repair

Bone Growth – Results

The trabecular bone volume to total volume fraction (BV/TV) was measured from CT images. The data shows that none of the treatments triggered any significant bone growth over time (Supplementary Figure 7), which is expected, given that the treatment is pro-angiogenic (VEGF-A delivery) rather than pro-osteogenic (BMP-2). More importantly, delivery of VEGF-A with TrAPs did not have a negative impact on osteogenesis, compared to empty sponges.

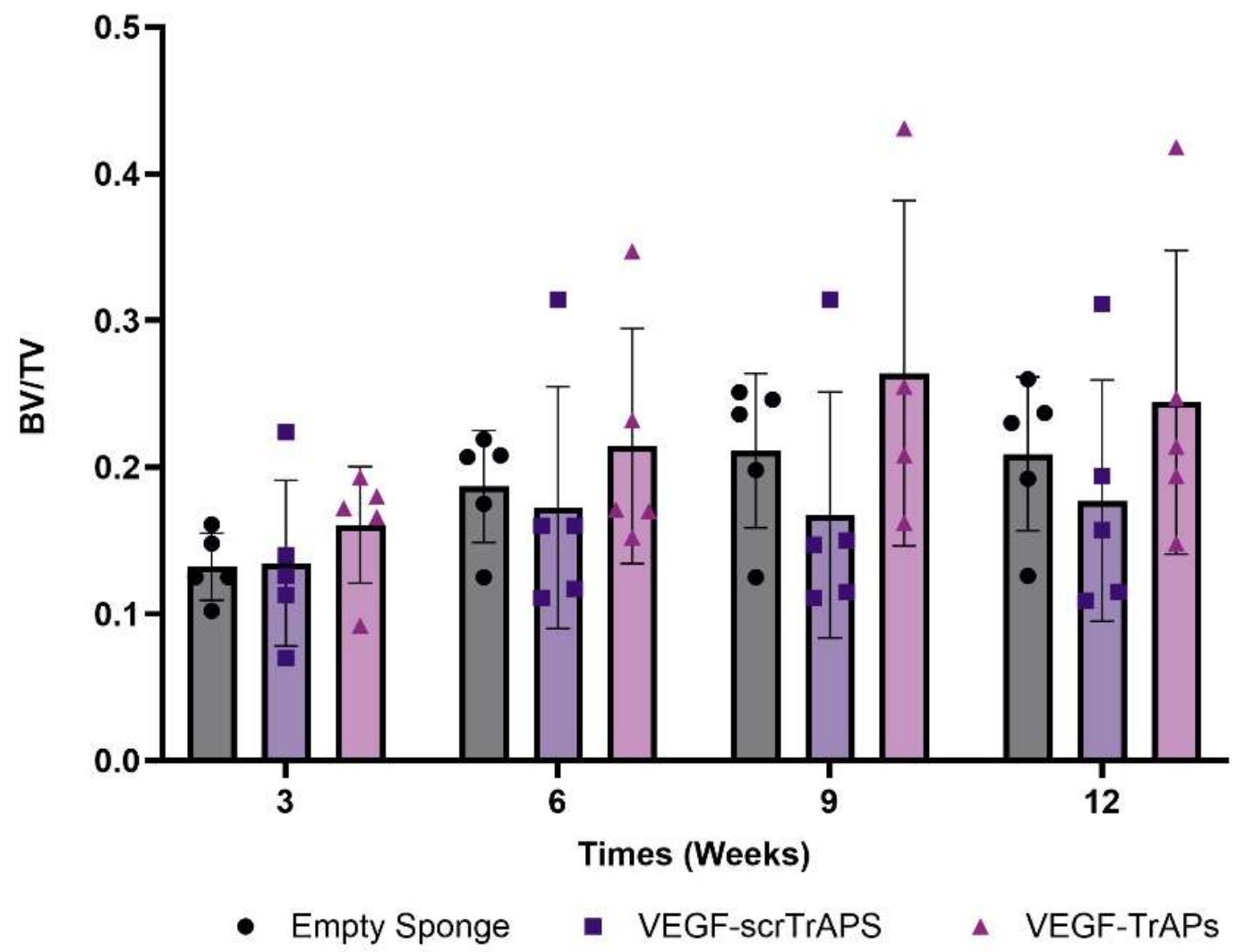

Supplementary Figure 7: Trabecular bone volume to total volume fraction (BV/TV) of rats following treatment. Quantification of BV/TV in rats treated with empty sponges, VEGF-TrAPs or VEGF-scrTrAPs at 3, 6, 9 and 12 weeks post-surgery. n = 5 animals per group unless otherwise states; n = 4 rats (VEGF-TrAPs at week 9). Statistical analysis was performed using two-way ANOVA followed by Tukey-Kramer post-hoc test. No statistically significant differences were detected between groups. Data are presented as mean ± SD.

Animal Well-Being – Results

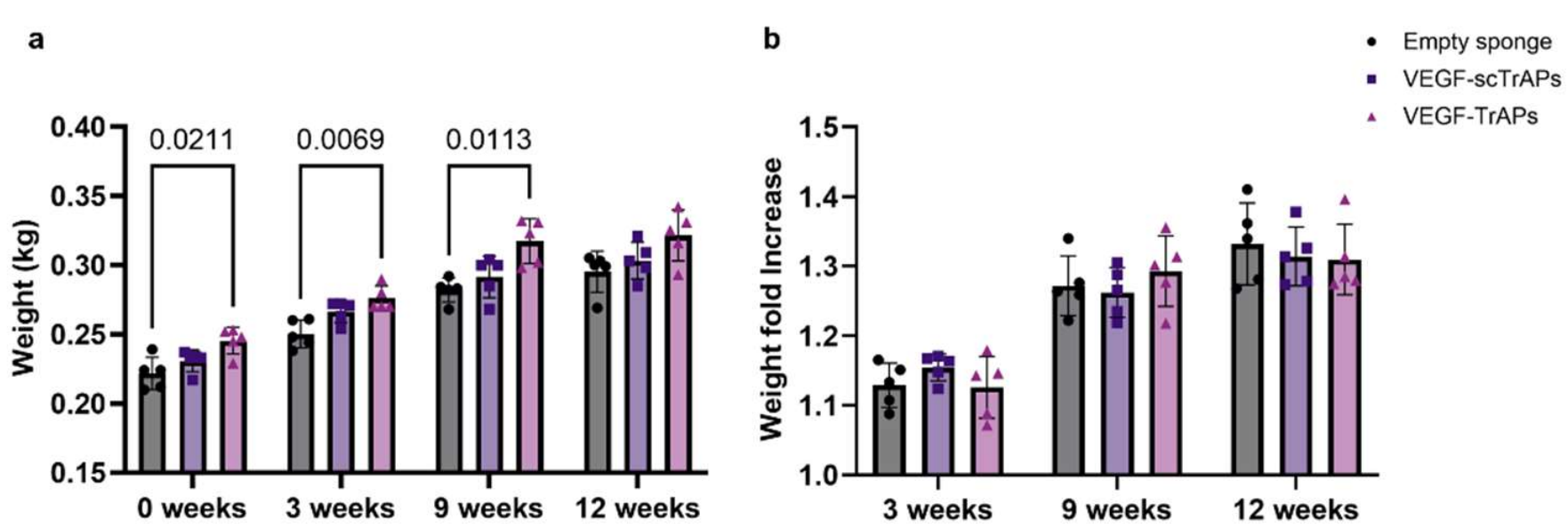

Supplementary Figure 8: Rat weight over experimental time. a, rat weight (kg) and **b,** weight fold increase over 12 weeks. For **a, b,** n = 5 rats. Statistical analysis was performed using two-way ANOVA followed by Tukey post-hoc test. Only comparisons where p≤0.05 are indicated on the graph. Data are presented as mean ± SD.

Adverse event	# Rats
Rat was lame for up to 3 days post-surgery	1/78
Wound breakdown post-surgery (without infection)	3/78
Issues placing/tightening screws	3/78
Apnoea after anesthesia induction, with cyanosis. (Rat was stabilized and had uneventful recovery).	1/78

Supplementary Table 5: Adverse effects and complications from surgical procedures, and their incidence.

Harvesting of Endogenous VEGF-A and HGF from Cells

Empty VEGF-TrAPs - Supporting Results

Collagen hydrogels functionalised with empty VEGF-TrAPs or empty scrTrAPs were not incubated with recombinant growth factors prior to seeding of cells. To account for any potential effects RGD peptide itself might have on HMEC-1 sprouting, a random sequence of single-stranded DNA (ssDNA) of the same length as TrAPs was conjugated to the RGD peptide (named: Random ssDNA_RGD) and functionalised in the tube formation and bead sprouting assay, incubated with VEGF-A121 and rinsed. However, the Random ssDNA_RGD neither promoted increased vascular area nor promoted longer sprouts compared to the scrTrAPs. This suggests that the increased vascular area and longer sprouts observed in the VEGF-TrAPs and empty VEGF-TrAPs group were due to the release of VEGF-A (Supplementary Figures 9 and 10).

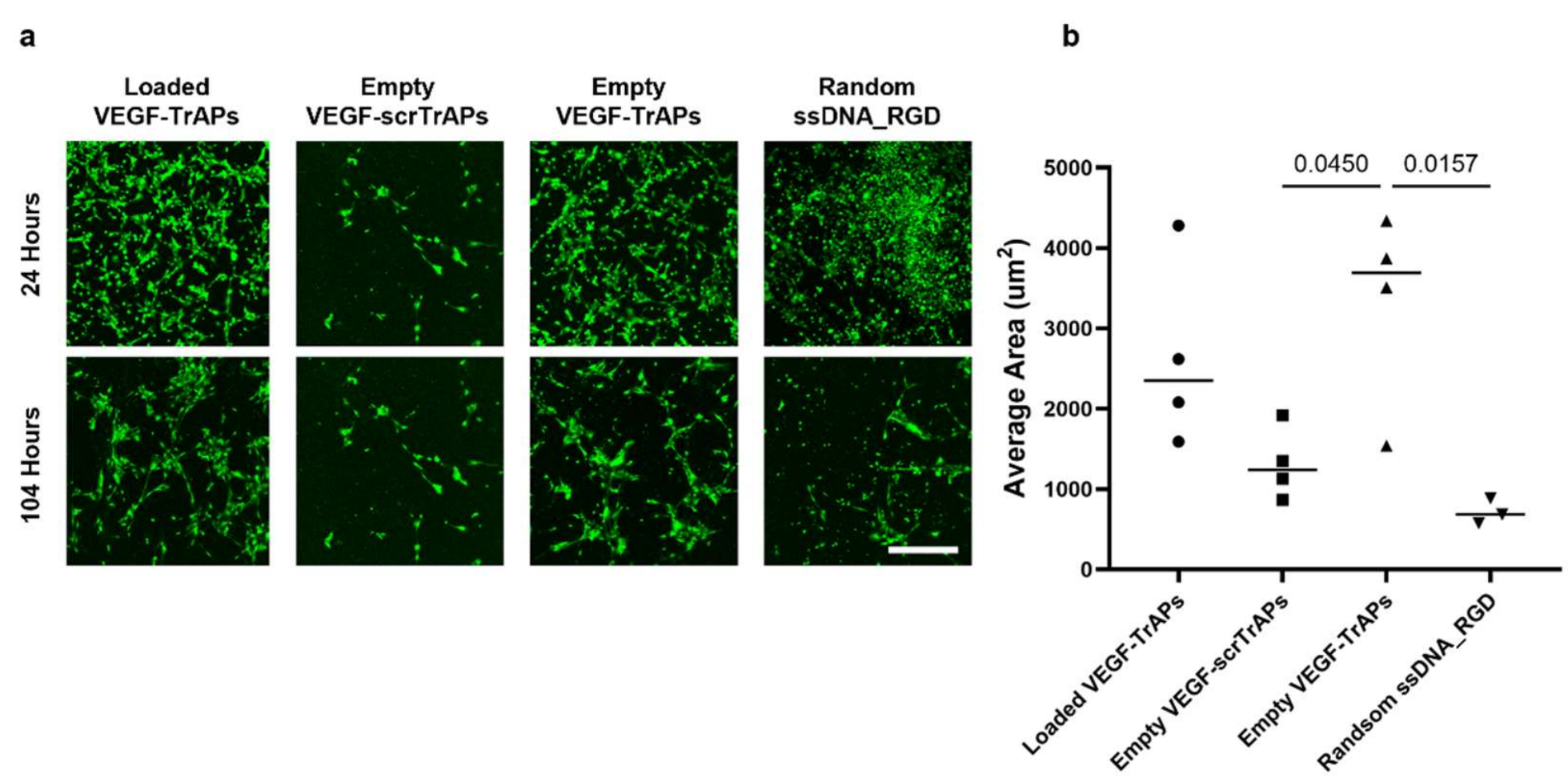

Supplementary Figure 9: Empty VEGF-TrAPs-functionalised collagen gel may support increased HMEC-1 vascular network areas. a, Maximum-intensity projection images of HMEC-1 networks 24 hours post seeding (Celltracker Green CMFDA: green, scale bar = 400 µm. **b,** Quantification of the mean vascular network area. n = 4 samples per group unless otherwise stated; Random ssDNA_RGD, n = 3 samples. For each sample, the mean vascular network area was calculated from identified network structures or objects within the analysed image. Statistical analysis was performed using one-way ANOVA followed by Tukey post-hoc test. Data presented as individual data points; horizontal lines indicate median.

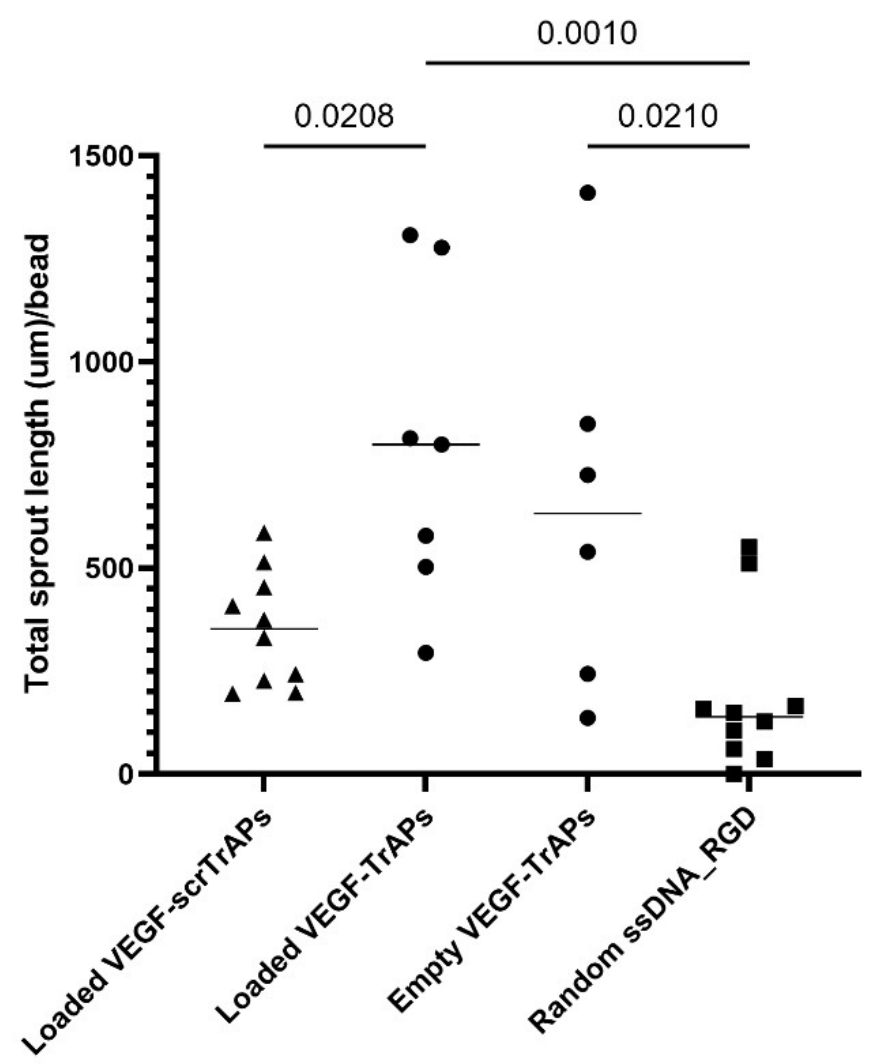

Supplementary Figure 10: In vitro endothelial sprouting in response to empty and VEGF-loaded TrAPs-functionalised collagen gels. Quantification of total endothelial sprout length per bead 24 hours post-seeding in empty VEGF-TrAPs, VEGF-loaded TrAPs, VEGF-loaded scrTrAPs and Random ssDNA groups. n = 10 beads (Loaded VEGF-loaded scrTrAPs, Random ssDNA), n = 7 beads (loaded VEGF-loaded TrAPs), n = 6 beads (empty VEGF -TrAPs). Statistical analysis was performed using one-way ANOVA followed by Tukey-Kramer post-hoc test. Data presented as individual data points; horizontal lines indicate median.

HMEC-1 Response to Recombinant HGF

HMEC-1 response to HGF - Supporting Results

HMEC-1 cells were first validated for their response to recombinant hepatocyte growth factor (HGF). After 24 hours, cell metabolic activity was measured using the PrestoBlue Cell Viability assay[16]. The metabolic activity of HMEC-1 increased in response to increasing doses of recombinant HGF (0.01 ng/mL, 10 ng/mL, 100 ng/mL, and 200 ng/mL), showing a dose-dependent effect (Supplementary Figure 11a). Since HGF is known as a scattering growth factor, a migration assay was also performed. HMEC-1 cells cultured on collagen beads were seeded on glass coverslips with or without the presence of HGF (Supplementary Figure 11b), in serum-reduced ECGM MV2 media. By 14 hours, HMEC-1 cultured in the presence of 100 ng/ml of HGF were qualitatively observed to scatter more from its origin (the closest bead).

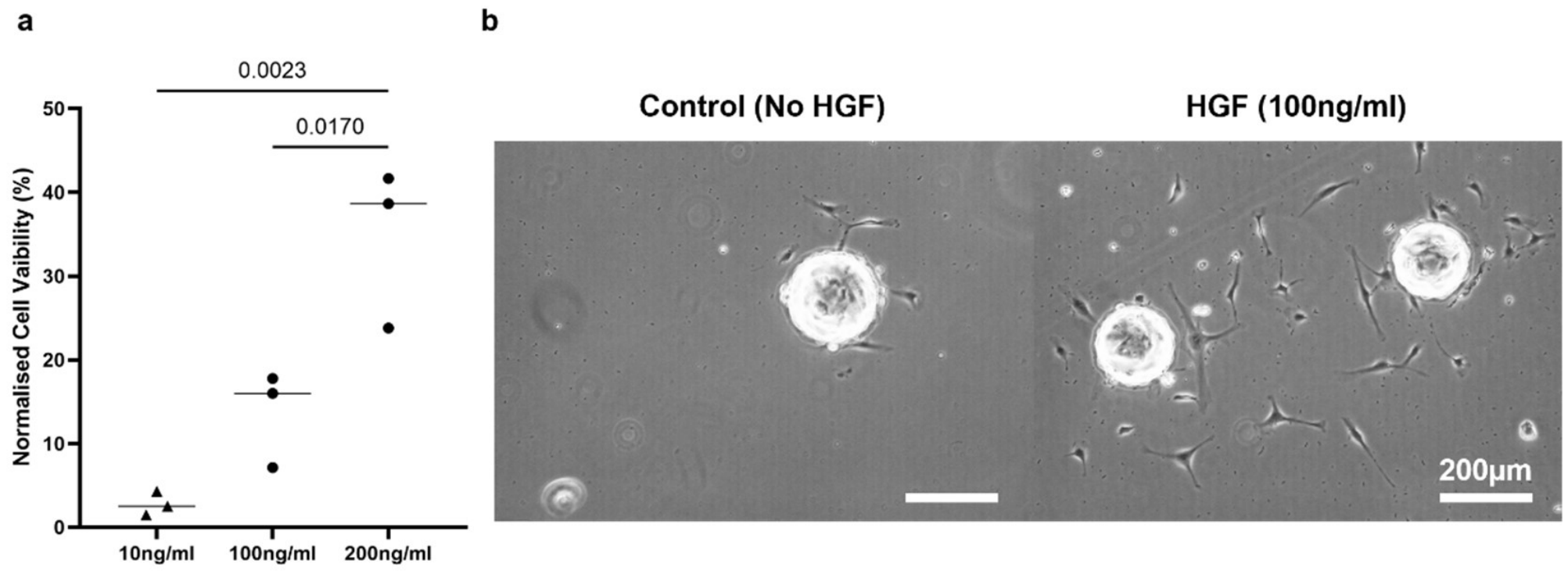

Supplementary Figure 11: HMEC-1 responses to hepatocyte growth factor (HGF). a, Dose response analysis of HMEC-1 exposed to recombinant HGF. **b,** Images of HMEC-1 migration from their origin (microcarrier beads), 14 hours post-seeding, in the presence of recombinant HGF. For **a,** n = 3 samples. Statistical analysis was performed using one-way ANOVA followed by Tukey post-hoc test. Data presented as individual data points; horizontal lines indicate median.

HMEC-1 response to HGF-TrAPs - Supporting Results

Once HMEC-1 were confirmed to be HGF responsive, experiments were conducted to evaluate the function and inhibitory capacity of the synthesised HGF-TrAPs (Supplementary Table 1). Between 24 hours and 100 hours, HMEC-1 cultured on HGF-TrAPs-functionalised coverslips, pre-loaded with recombinant HGF, scattered farther away from their origin (the nearest bead) compared to cells on cysteine or scrTrAPs-functionalised coverslips (Supplementary Figure 12); The HGF-functionalised coverslips supported a greater number of migrated cells, which in turn supported a significantly greater total distance covered by migrated cells per bead (Supplementary Figure 12b,c). Together, these results suggest the successful inhibition and delivery of recombinant HGF through activation of HGF-TrAPs by HMEC-1 cells.

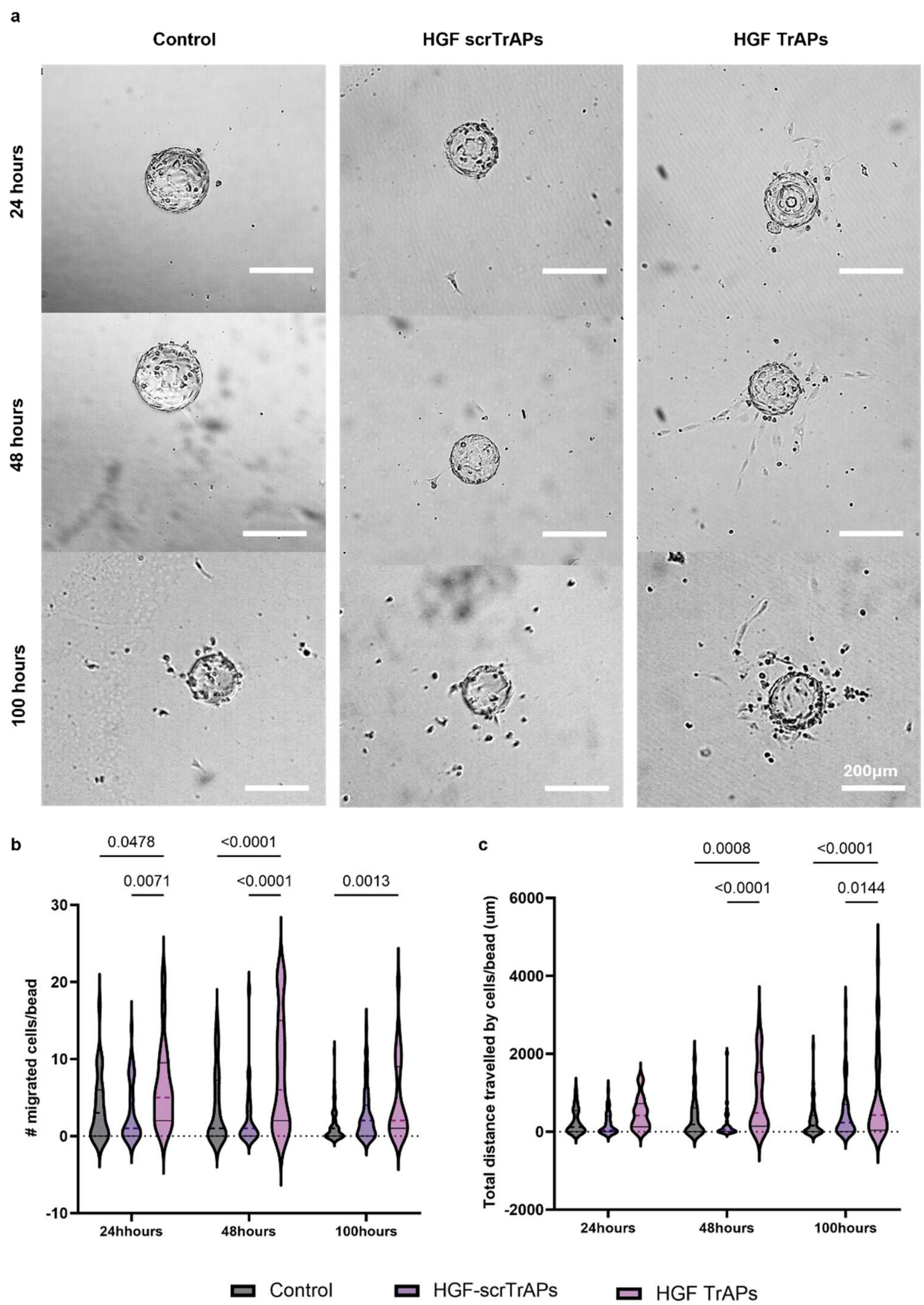

Supplementary Figure 12: HGF-TrAPs mediated HMEC-1 migration on functionalised coverslips. a, Images (**a**) and quantification of HMEC-1 **(b, c)** migration at 24, 48 and 100 hours. Functionalised coverslips were loaded with 200 ng/ml of recombinant HGF overnight before being rinsed thoroughly and seeded with HMEC-1 cell-coated microcarrier beads. **b,** Number of migrated cells per bead. **c,** Total distance travelled by migrated cells per bead. For **b**, **c,** number of analysed beads were: 24 hours, n = 30 (control), n = 38 (HGF-scrTrAPs), n = 37 (HGF-TrAPs) beads. 48 hours, n = 46 (control), n = 30 (HGF-scrTrAPs), n = 47 (HGF-TrAPs). 100 hours, n = 48 (control), n = 55 (HGF-scrTrAPs, HGF-TrAPs). Statistical analysis was performed using two-way ANOVA followed by Tukey-Kramer post-hoc test. Data presented as violin plots; horizontal dashed lines indicate median and horizontal solid lines indicate quartiles.

Human Dermal Fibroblast Response to Recombinant FGF-2

HDF Dose-Response to FGF-2 – Supporting Results

PDGF-BB-TrAPs[16], VEGF-TrAPs[16] and HGF-TrAPs were previously validated to bind and inhibit recombinant growth factors. To validate FGF-2-TrAPs, Passage 2 human dermal skin fibroblasts (HDF) were first validated for its response to Human FGF-2 (Thermofisher, PHG0261).

4,000 HDF cells were seeded onto each well of a 96-well plates and incubated with supplemented low glucose DMEM (10% FBS, 100 U/ml penicillin-streptomycin, 2 mM GlutaMax) for 24 hours. The supplemented media was then replaced with starvation media (DMEM low glucose, 0.1% FBS, 100 U/ml penicillin-streptomycin, 2 mM GlutaMax) and incubated overnight, for at least 16 hours. After which, the starvation media was replaced with appropriate doses of human recombinant FGF-2 in starvation media. After 48 hours of incubation, cells were incubated for 2 hours with 1:9 volumetric ratio of PrestoBlue to media and measured for its fluorescence. The relative cell viability between treatment groups were then quantified and normalised based on the fluorescence signal detected. Cell viability of HDF were observed to increase in response to higher concentrations of FGF-2, confirming that HDFs are responsive to FGF-2 (Supplementary Figure 13).

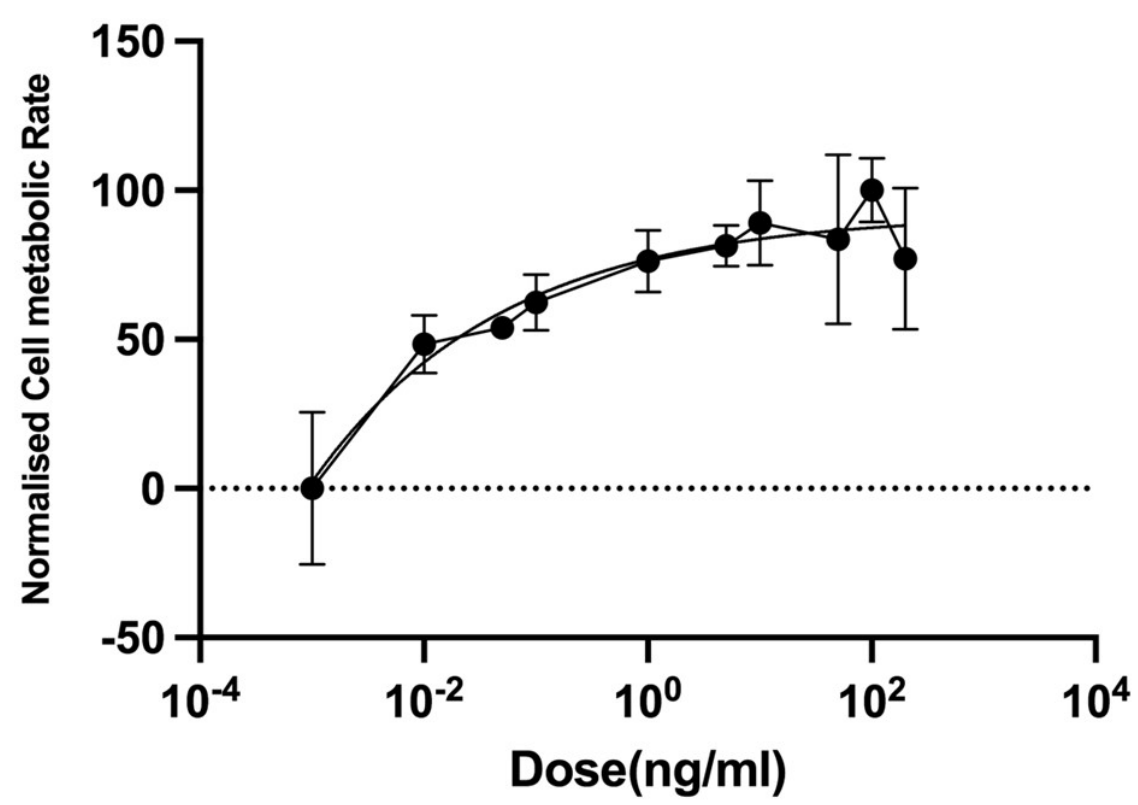

Supplementary Figure 13: Human dermal fibroblast (HDF) response to recombinant FGF-2. HDF cell viability was measured across increasing concentrations of recombinant FGF-2. n = 4 samples per concentration unless otherwise stated; 0.001 ng/ml FGF-2, n = 2 samples. Data presented as mean ± SD.

HDF Response to FGF-2-TrAPs – Supporting Results

To determine if FGF-2-TrAPs are inhibitory and can be used to deliver FGF-2 in response to cellular traction forces, FGF-2-TrAP-functionalised collagen sponges were incubated with recombinant FGF-2 (rhFGF-2), washed, and then seeded with HDFs. After 4 days, the collagen sponges functionalised with FGF-2 supported a significantly higher number of HDFs compared to sponges functionalised with scrTrAPs or cysteine (Supplementary Figure 14a, b). These results suggest the successful inhibition and delivery of FGF-2.

In a complementary migration assay, FGF-2-TrAPs were assessed for their ability to promote HDF migration. HDFs cultured on cytodex microcarrier beads were encapsulated within cysteine-, scrTrAPs- or TrAP-functionalised collagen hydrogels. After 96 hours, migrated cells were classified based on morphology, and the proportion of elongated cells were quantified. Cells exhibiting lengths greater than the mean cell length were considered elongated, whereas shorter cells were classified as non-elongated. FGF-2-TrAPs and soluble rhFGF-2 (positive control) supported a higher percentage of elongated cells compared to the cysteine control (negative control) and FGF-2 scrTrAPs group (Supplementary Figure 14 c, d).

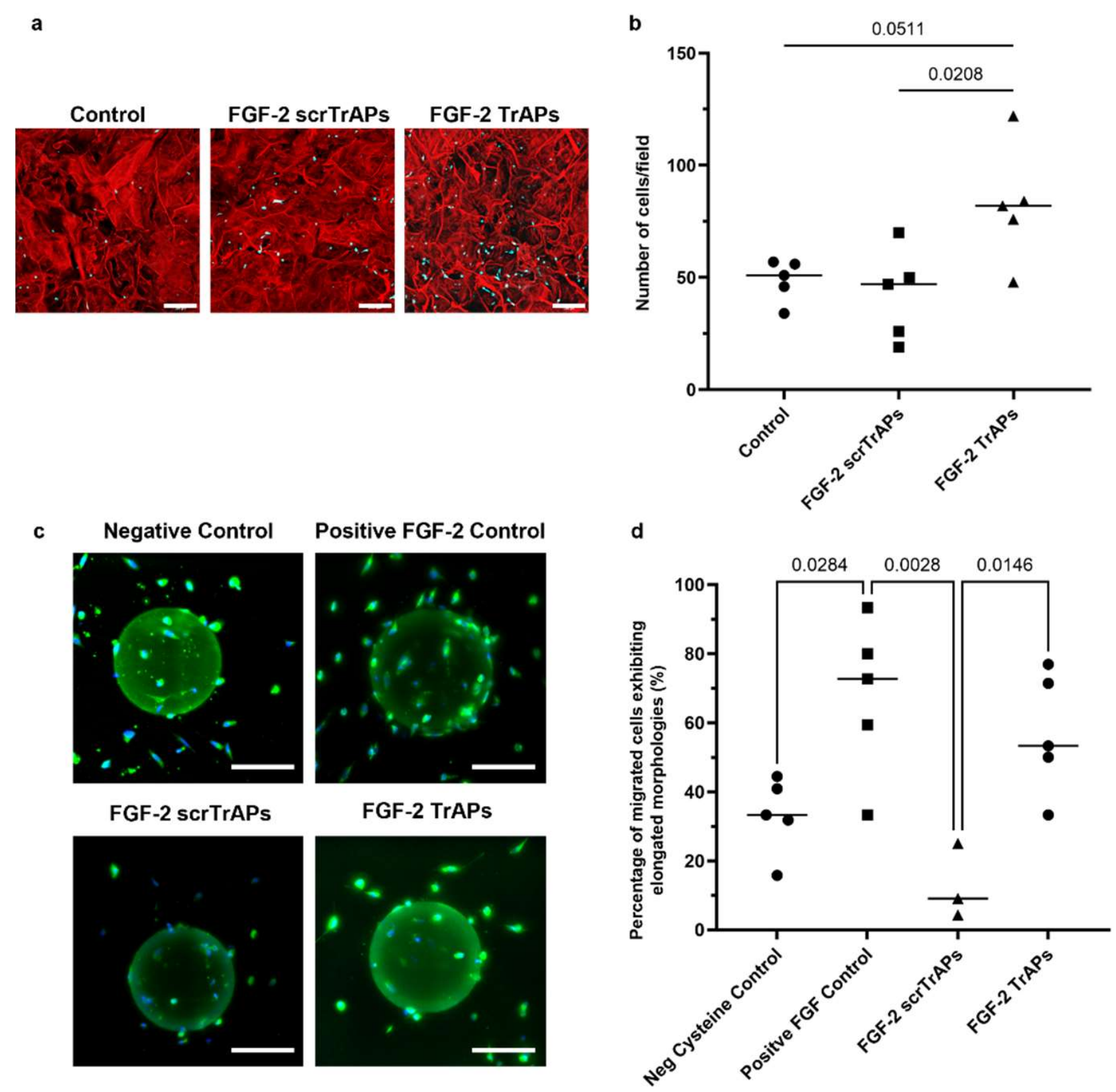

Supplementary Figure 14: FGF-2-TrAPs-mediated human dermal fibroblast proliferation and migration in vitro. a, Fluorescence images of HDFs cultured within control, FGF-2 scrTrAPs and FGF-2-TrAP-functionalised collagen sponges at 96 hours post-seeding (CellTracker Green CMFDA: cyan, Texas Red-labelled sponge: red. Scale bar: 200 µm). **b,** Quantification of HDF number within the captured imaging field of collagen sponges. **c,** Maximum intensity projections images of HDF migration from microcarrier beads in collagen gel, 96 hours post-seeding (DAPI: blue, F-actin: green, scale bar: 100µm). **d,** Percentage of migrated cells with elongated morphologies, defined as cells with lengths greater than the mean cell length of the negative control group. For **b,** n = 5 sponges per group. For **d,** n = 5 beads per group unless otherwise stated; FGF-2 scrTrAPs, n = 3 beads. For **b, d,** statistical analysis was performed using one-way ANOVA followed by Tukey-Kramer post-hoc test. Data presented as individual data points; horizontal lines indicate median.

Quantification of Growth Factors in Blood Lysate

To detect the presence of these growth factors in hPL, an enzyme-linked immunosorbent assay (PDGF BB R&D Systems DY220, FGF-2 R&D Systems DY233, HGF Cambridge Bioscience ELH-HGF-CL-1 and VEGF R&D Systems, DY293B) was used (Supplementary Table 6) according to the manufacturer's protocol. The average concentration of PDGF-BB, FGF-2, HGF and VEGF-A were found to be 6900 pg/ml, 660 pg/ml, 2250 pg/ml and 650 pg/ml respectively.

Estimated concentration of growth factor in HPL	
Growth Factor	Concentration (pg/ml)
PDGF-BB	6900
FGF-2	660
HGF	2000-2500
VEGF-A	600-700

Supplementary Table 6: Estimated concentration of growth factors present in HPL as measured against the standards, with ELISA kits. A range is given when two different batches of HPL was measured.

Implications for Maximum TrAP Loading – Supplementary Notes

These concentrations influence the maximum possible loading of growth factors onto TrAPs from hPL. However, it is important to highlight a fundamental conceptual point: for the TrAPs system, the total amount of growth factor bound to TrAPs is not equivalent to the therapeutically active dose, unlike conventional controlled-release systems.

In conventional controlled-release systems such as degradable polymers, hydrogels, and microspheres, growth factors are released passively into the surrounding environment, where they act in bulk solution. In these systems, the total loaded amount and cumulative release profile are appropriate measures of therapeutic input because the loaded dose is a reasonable measure of the active dose. With TrAPs, the mechanism is fundamentally different: growth factors are bound and inhibited by high-affinity aptamers and are released only upon mechanical unfolding by cellular traction forces at the point of cell-material contact. Growth factors that remain bound are not biologically active. Passive release is near or below the detection limits of standard ELISA assays, meaning that the vast majority of bound growth factor remains inactive in the absence of cellular engagement.

The scrTrAP controls provide direct experimental evidence for this distinction. scrTrAPs and TrAPs are loaded with identical concentrations of growth factor from hPL (or recombinant growth factor for the rat femur experiment), creating the same total bound amount, yet produce dramatically different biological outcomes across every experimental system tested in this study: in vitro sprouting and migration assays, ex vivo human skin integration, in vivo rat femur vascularisation, and in vivo mouse wound closure. If total bound dose were the relevant quantity, scrTrAPs and TrAPs would produce identical outcomes. However, the combined results demonstrate that the biologically

relevant dose is not the total amount bound to the scaffold, but the amount released by cell-mediated activation, a quantity that is inherently dynamic, spatially localised, and cell-dependent.

Therefore, it is worth highlighting that the total bound dose is not a representative measure for therapeutic activity. Reporting it as such can misrepresent the system by suggesting that cells are exposed to the full amount, when in reality only a small, cell-activated fraction is biologically active at any given time. This is demonstrated clearly in the rat femur experiment, where despite adding supplemental recombinant VEGF-A to the scrTrAP group, the biological response corresponds to an available VEGF-A level that is below the endogenous level during repair, despite additional VEGF-A being present. This concept is a defining feature of the TrAP mechanism, rooted in the bioinspired design of the strategy, and the primary reason TrAPs can operate at doses orders of magnitude below conventional delivery systems while still producing measurable biological effects.

Thus, we have focused on using functional cell and tissue responses - which directly report on whether growth factor release has reached a biologically stimulatory threshold, as the primary and most appropriate measure of "dose" for this system. To contextualise the doses used in this study and assess their physiological relevance, we additionally present a theoretical upper-bound estimate of growth factor loading onto TrAP-functionalised scaffolds from hPL. We emphasise that this calculation deliberately overestimates actual loading and is presented specifically to demonstrate that even under the most favourable assumptions, TrAP-associated doses remain orders of magnitude below existing clinical standards. The actual effective dose delivered to cells is lower still for several reasons:

(i) Competitive binding and steric hindrance: hPL contains thousands of proteins, many of which will compete with target growth factors for non-specific adsorption sites on the scaffold and may partially occlude aptamer binding sites or compete with TrAPs for binding of the targeted growth factors.
(ii) Growth factor degradation: proteases present in hPL will degrade a fraction of the available growth factors during the incubation period, reducing the amount available for capture.

Therefore, we present the following upper-bound calculation, which assumes 100% capture of all available growth factor molecules by TrAPs, which is an assumption that overestimates both the actual loading and the therapeutic dose that is activated by cells.

The large molar excess of aptamers over growth factors (TrAPs functionalised at 1 µM; growth factor concentrations in 1/3-diluted hPL: VEGF-A: 0.01 nM; FGF-2: 0.01 nM; HGF: 0.01 nM; PDGF-BB: 0.09 nM) means that the aptamers are present in approximately 1,000-fold excess over their targets. For the weakest-affinity aptamer used (HGF, $\mathrm{K_d} = 20$ nM), equilibrium binding can be calculated as:

$$K_D = \frac{[Aptamer]_{free}[HGF]_{free}}{[AptamerHGF]_{bound}}$$

With $[\text{Aptamer}]_{total} = 1000$ nM and $[\text{HGF}]_{total} = 0.01$ nM, solving the quadratic gives $[\text{AptamerHGF}]_{bound} = 0.0098$ nM, corresponding to ~98% capture efficiency. For higher-affinity aptamers (VEGF-A: $K_d = 0.2$ nM; PDGF-BB: $K_d = 0.1$ nM; FGF-2: $K_d = 3$ nM), capture efficiencies approach 100% under these conditions. This confirms that the limiting factor on loading is the amount of growth factor present in hPL, not the capture efficiency of the aptamers, meaning that the 100% retention assumption used below is a reasonable upper bound on the amount of growth factor that could possibly be loaded, noting that the factors above that will reduce actual loading.

Importantly, even under this most conservative upper-bound assumption, TrAP-associated doses are orders of magnitude lower than existing clinical growth factor delivery strategies, as shown in Supplementary Table 8. Moreover, the actual effective dose at the cell surface is lower still, because:

(i) only a small fraction of loaded TrAPs are activated simultaneously by cells
(ii) growth factors released locally at the cell-material interface are unlikely to significantly increase bulk solution concentrations
(iii) cells co-localise receptor tyrosine kinases near integrins, increasing the probability of local receptor engagement following release.

This implies that TrAPs promote efficient biological responses at doses that are lower than the upper-bound calculations suggest and diverge from responses associated with a total concentration of growth factor.

2D Proliferation and Migration Assays – Method Notes

	Cysteine	scrTrAPs	TrAPs	scrRDG peptide (G**R<u>D</u>G**SPC)	RGD peptide (G**RGD**SPC)
Control Cysteine group	3.5 µM	-	-	3.5 µM	25 µM
scrTrAPs	-	3.5 µM	-	-	25 µM
TrAPs	-	-	3.5 µM	3.5 µM	21.5 µM

Supplementary Table 7: Concentrations of cysteine, scrTrAPs, TrAPs, scrambled (scr) RDG peptide and RGD peptide used in the functionalisation of 2D coverslips. Total RGD and scrRDG concentrations on the coverslips were matched across groups, to ensure cell behaviour in assays were not biased by the available amount of adhesive peptides.

HDF response to HPL-loaded PDGF-BB-TrAPs – Biological Repeats

To account for donor-bias observations, experimental conditions were repeated with HDF isolated from additional donors, on 2D coverslips (Supplementary Figure 15) and 3D collagen sponges (Supplementary Figure 16). All repeats demonstrated a similar trend, where PDGF-TrAPs supported significantly higher HDF cell numbers than the control and scrTrAPs group.

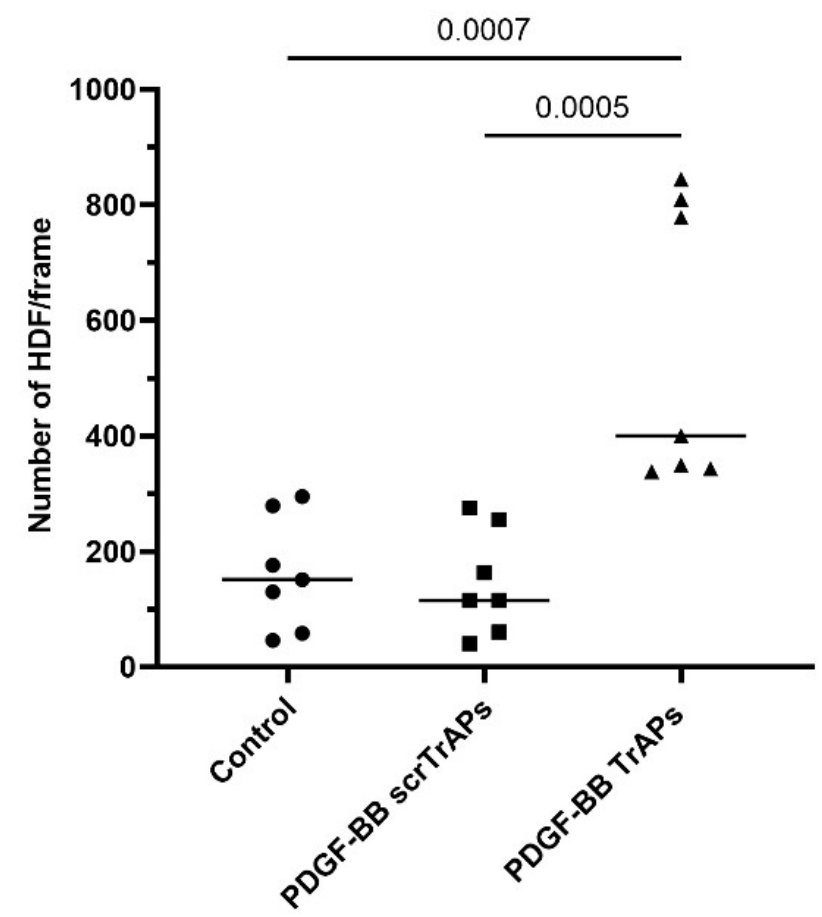

Supplementary Figure 15: In vitro validation of PDGF-BB-TrAPs-mediated HDF proliferation on coverslips, using a biologically different donor. Experimental repeat of Figure 4bi using HDFs isolated from a different biological donor. Quantification of HDF number within imaging fields captured from the control, PDGF-BB scrTrAPs and PDGF-BB-TrAPs group, 72 hours post-seeding. n = 7 coverslips per group. Statistical analysis was performed using one-way ANOVA followed by Tukey post-hoc test. Data presented as individual data points; horizontal lines indicate median.

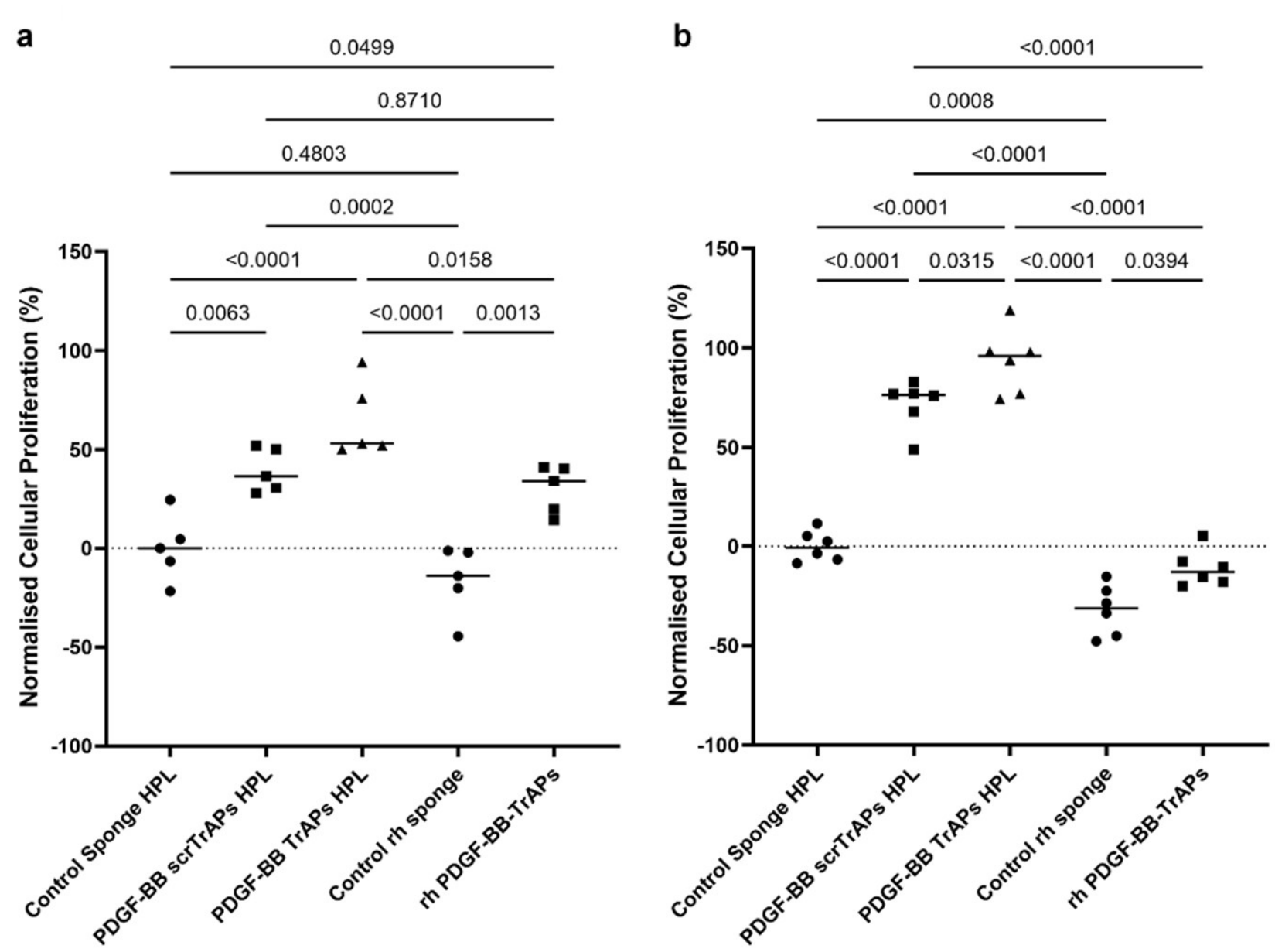

Supplementary Figure 16: In vitro validation of PDGF-BB-TrAPs-mediated HDF proliferation on 3D collagen sponges, using biologically different donors. Experimental repeats of Figure 4bii performed using HDFs isolated from two additional biological donors, to account for donor variability. Control sponges do not contain scrTrAPs or TrAPs but were subjected to the same loading conditions, using either human platelet lysate (HPL) or recombinant human PDGF-BB (rh PDGF-BB), and subsequent washing procedures as the scrTrAPs and TrAPs group. PrestoBlue Cell Viability Reagent was used to quantify cellular metabolic activity as a measure of proliferation. **a,** Donor 1, n = 5 sponges per group. **b,** Donor 2 , n = 6 sponges per group. Statistical analysis was performed using one-way ANOVA followed by Tukey post-hoc test. Data presented as individual data points; horizontal solid lines indicate median.

Combinatorial TrAPs - Supplementary Results

FGF-2 and PDGF-BB-TrAPs Collagen Sponge In Vitro Evaluation – Preliminary Study

The use of multiple TrAPs to harvest and deliver a combination of growth factors from hPL were first evaluated in a preliminary in vitro model. A combination of FGF-2-TrAPs and PDGF-BB-TrAPs, as well as their respective scrTrAPs, were functionalised on collagen sponges, incubated with hPL, washed and cultured with HDF (Supplementary Figure 17). 2 weeks post-seeding, significantly increased HDF cell numbers were observed on sponges functionalised with both FGF-2 and PDGF-BB-TrAPs, compared to all other groups (Supplementary Figure 17), including those functionalised with at least one type of TrAPs (eg. PDGF-BB-TrAPs + FGF-2 scrTrAPs or FGF-2-TrAPs + PDGF-BB-scrTrAPs). This is unsurprising, as FGF-2-TrAPs and PDGF TrAPs have both been demonstrated to promote increased cell proliferation, and hence, their combined effects were expected to be greater than each individually. Thus, this preliminary study supports the use of TrAPs to deliver multiple endogenous growth factors, to promote cellular processes. Interestingly, the combination of TrAPs + scrTrAPs of another growth factor did not promote increased HDF numbers compared to the cysteine control group. This lack of effect could be due to empty scrTrAPs capturing endogenously produced growth factors from HDF, limiting the effect of the other TrAPs group. Moreover, with 1 μM of either TrAPs or scrTrAPs functionalised to the sponges and only about 0.37 pM PDGF-BB (9.2 mg/ml in hPL) or 0.0275 pM FGF-2 (0.66 ng/ml in hPL) from hPL bound to TrAPs, there are approximately one million times more available scrTrAPs that can harvest away secreted growth factors from HDF. Nonetheless, this initial study supports the benefits of delivering multiple growth factors to promote cell proliferation/survival.

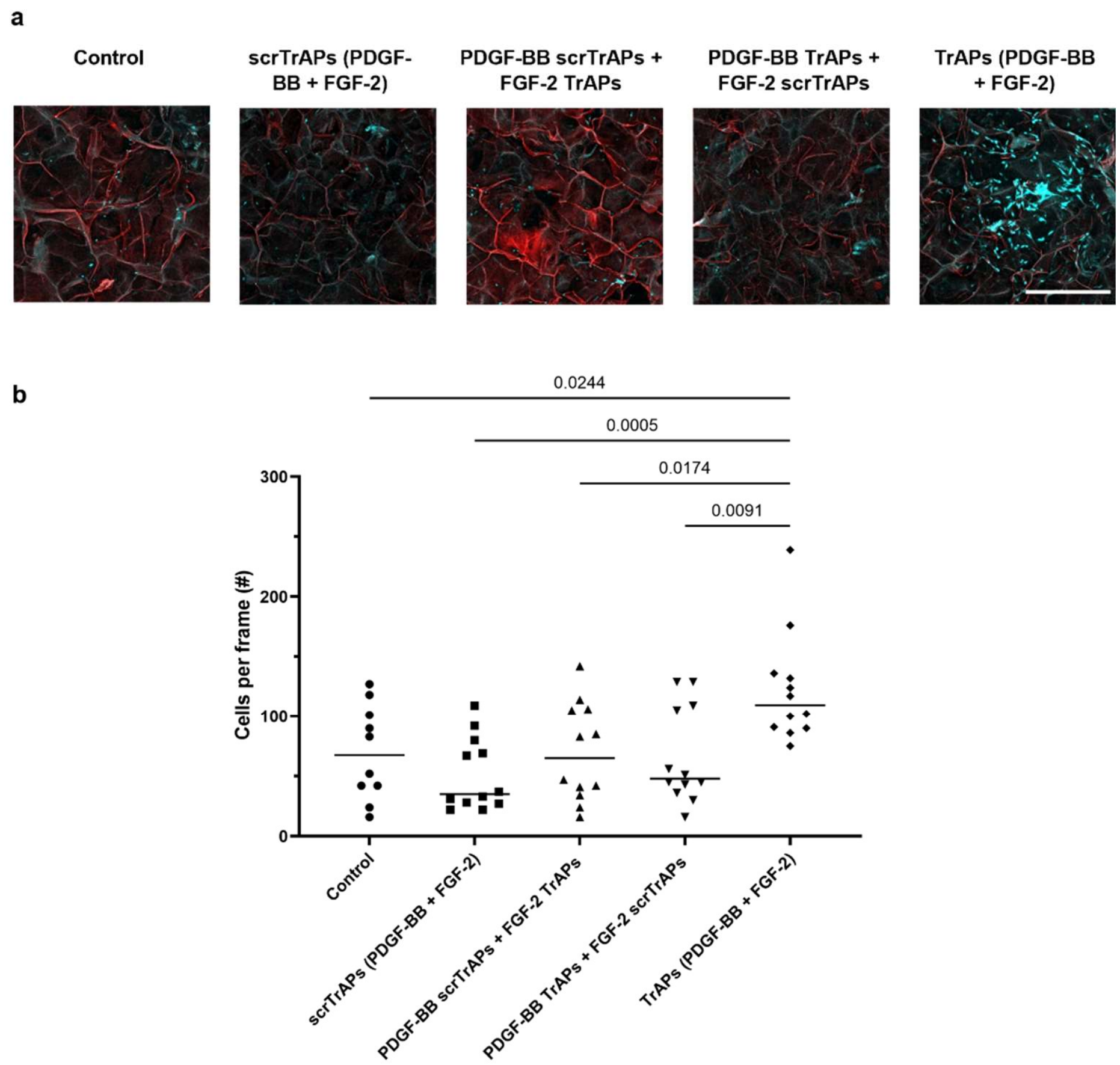

Supplementary Figure 17: Combined PDGF-BB- and FGF-2-TrAPs-mediated HDF proliferation in collagen sponges. a, Maximum intensity projection fluorescence images of HDF in collage sponge 2 weeks post-seeding (cyan: *CellTracker Green CMFDA*, red: Texas Red-labelled sponge, scale bar: 500 µm). **b,** Quantification of HDF number within imaging fields captured 2 weeks post-seeding. n = 12 imaging fields per group (2 imaging fields per sponge) unless otherwise indicated; control n = 10 imaging fields. Statistical analysis was performed using one-way ANOVA, Tukey-Kramer post-hoc test. Data presented as individual data points; horizontal solid lines indicate median.

FGF-2, PDGF-BB and HGF-TrAPs Collagen Sponges In Vitro Evaluation – Supporting study

A combination of three growth factors from human platelet lysate (hPL) - FGF-2, PDGF-BB, and HGF - was subsequently assessed in vitro (Supplementary Figure 18). After 4 days, the combined TrAPs group (FGF-2, PDGF-BB and HGF) promoted significantly greater number of HDFs compared to their individual TrAPs groups, collective scrTrAPs group, and cysteine control group (Supplementary Figure 18a,b). Interestingly, even the combination of scrTrAPs led to an increase in cell numbers compared to the cysteine control group, albeit to a significantly lower extent than the combination of TrAPs. This observation is likely due to the small amount of passive release of PDGF-AA, which can bind to PDGF-BB-TrAPs with low affinity (PDGF-AA K_d=72 nM versus PDGF-BB K_d=0.093 nM)[2], combined with otherwise insignificant leakage of FGF-2 and HGF from their respective scrTrAPs may have positively influenced cell survival and/or proliferation when present in combination[17]. Nonetheless, this study further validates the increased therapeutic efficacy of using multiple types of TrAPs, in combination, to harvest and deliver complementary endogenous growth factors.

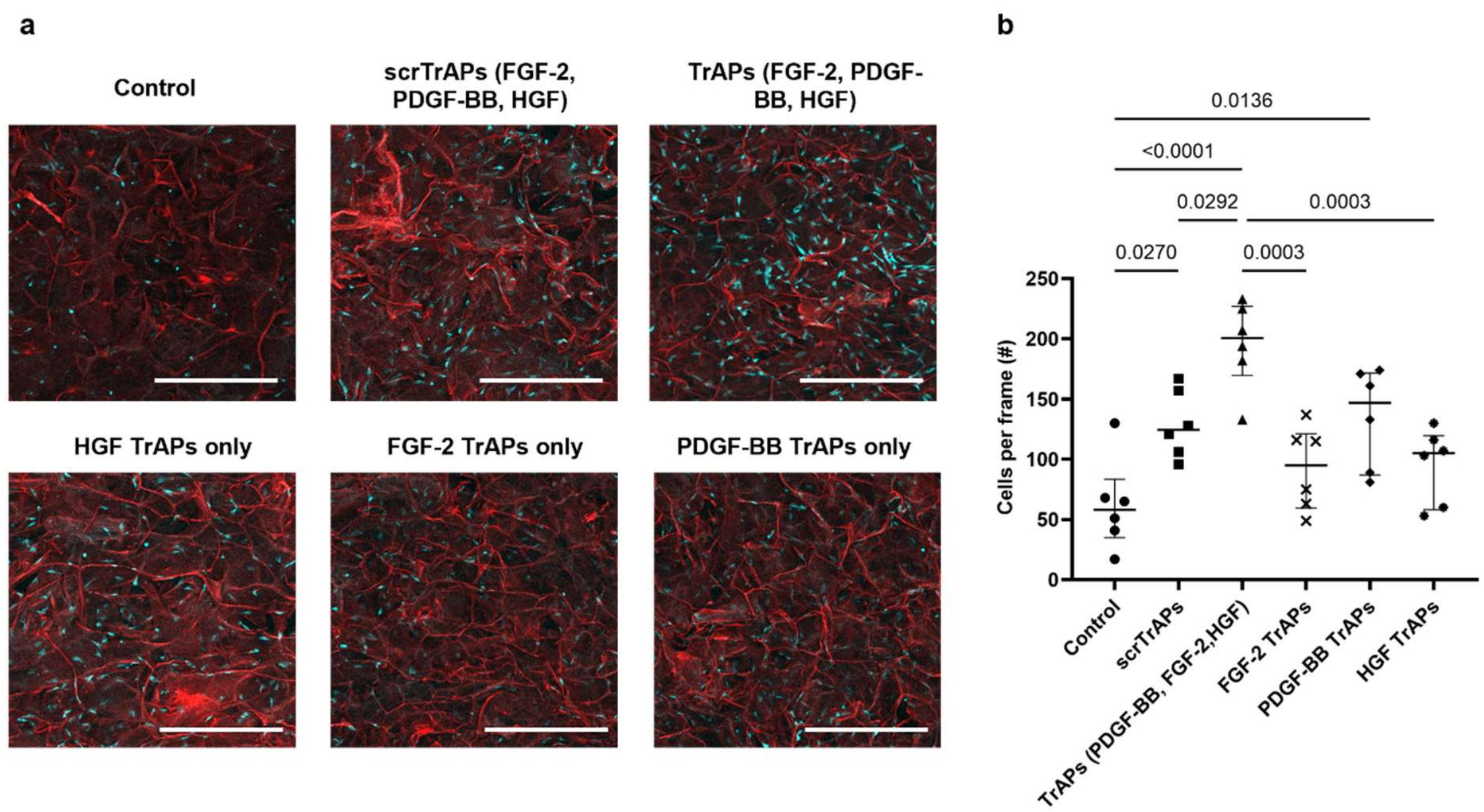

Supplementary Figure 18: Combination of PDGF-BB, FGF-2 and HGF-TrAPs promote HDF proliferation within collagen sponges. a, Fluorescence images of HDFs (cyan) cultured on functionalised collagen sponges (red) 4 days post-seeding. Scale bar: 500 µm**. b)** Quantification of HDF number within z-stacked confocal imaging fields. n = 6 sponges per group. Statistical analysis was performed using one-way ANOVA followed by Tukey post-hoc test. Data presented as median. Data presented as individual data points; horizontal solid lines indicate median.

FGF-2, PDGF-BB and HGF-TrAPs in Ex Vivo Human Skin – Preliminary study

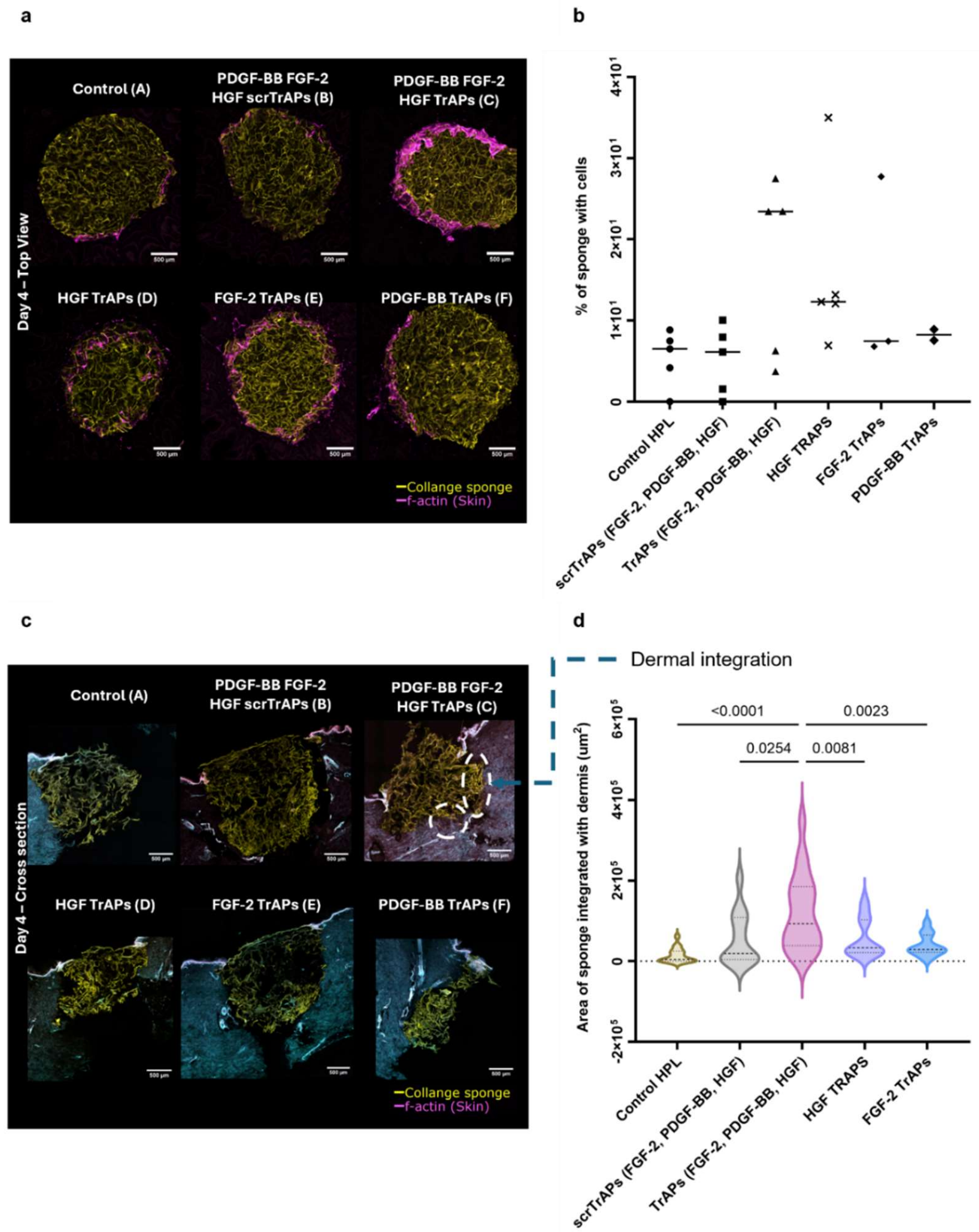

Supplementary Figure 19: Assessment of skin-sponge integration in ex vivo human skin wounds. a, Whole mount skin wounds (F-actin: Magenta, Texas Red-labelled sponge: yellow), 4 days post-injury. Scale bar: 500 µm. **b,** Quantification of skin-sponge overlap from confocal images of whole-mount skin. **c,** Skin cross-sections 4 days post-injury (DAPI: cyan, f-actin: magenta, sponge: yellow). Scale bar: 500 µm. **d,** Quantification of dermal-sponge integration from skin cross-sections. For **b,** n = 5 skin samples per group unless otherwise indicated; FGF-2-TrAP, n = 3 samples and PDGF-BB-TrAPs, n = 2 samples. Data presented as individual data points; horizontal solid lines indicate median. For **d,** n = 17 (control), n = 13 (scrTrAPs, FGF-2-TrAPs), n = 20 (combined TrAPs), n = 21 (HGF-TrAPs) skin cross-sections. Data presented as violin plots; horizontal dashed lines indicate median and horizontal dotted lines indicate quartiles. For **b, d,** statistical analysis was performed using one-way ANOVA followed by Tukey-Kramer post-hoc test.

Ex Vivo Human Skin Model – Method Notes

Parameters	Microscope setting
UV laser for DAPI (nuclei)	405 nm excitation
Super continuum white light laser for Actin 555ready probe (f-actin)	541 nm excitation
Super continuum white light laser for Texas Red (sponges)	595 nm excitation
Objective	HC PL APO CS2 10x/0.40 DRY
Format	1024x1024
Speed	600
Pinhole	3.00 AU
Zoom factor	1
Z section size	260 µm
Z-step size	20.00 µm
Field size (for tilescan)	4x4
Merge	10% overlap, smooth

Supplementary Table 8: Microscope settings for ex vivo whole mount skin imaging

Parameters	Microscope setting
405nm laser for DAPI (nuclei)	405 nm excitation
White Light Laser for Actin 555ready probe (f-actin)	541 nm excitation
White Light Laser for Texas Red (sponges)	595 nm excitation
Objective	HCPL APO CS2 10x/0.40 DRY
Format	1024x1024
Speed	600
Pinhole	2.00 AU
Zoom factor	1
Z section size	160 µm
Z-step size	20.00 µm
Field size (for tilescan)	Dependent on cross section
Merge	10% overlap, smooth

Supplementary Table 9: Microscope settings for ex vivo histology cross-section skin imaging

Tensile Pull-Out Test of Sponge-Skin Mechanical Interface

Supplementary Discussion

During sponge pull-out, occasional stretching of skin attached to the sponge was observed. Varying failure interfaces - between skin-skin, skin-sponge and sponge-sponge - were also observed across and within samples. These variations did not appear to correlate strongly with force-displacement graphs, peak force or work done. Confocal imaging following pull-out revealed uneven dermal surfaces (Extended Data Figure 4), suggesting potential tissue disruption caused by the test. Fluorescently labelled sponge fragments (with Texas Red) were detected in the wound bed post-removal, and sectioned wound slices showed regions where the collagen sponge had likely been recently degraded by the healing skin, retaining fluorescence in the Texas Red channel (Extended Data Figure 4). These findings suggest that by Day 8, regions of integrated collagen sponges are actively being degraded and replaced by new tissue.

This interplay between integration and degradation may account for the absence of correlation between mechanical testing results and the observed degree of skin integration. Additionally, the mechanical stress applied during the pull-out tests at early healing stages (Day 5-8), likely disrupted the fragile, newly formed skin matrix. Therefore, chemical fixation with PFA prior to analysis appears to be the most reliable method to preserve the integrity of skin-sponge interactions. Quantifying the probability of sponge retention after fixation and sectioning (Extended Data Figure 4a) may offer a more accurate proxy for skin-sponge integration, as poor integration would result in insufficient crosslinking, rendering the sponge weaker than even immature tissue matrices.

In Vivo Imaging of Mouse Skin Wound Healing

On day 3 and day 10 post-implantation, 40% and 60% of the mice respectively, were anaesthetized and imaged using a Bruker Ultima Investigator laser scanning microscope and a Spectra-Physics Ti:sapphire laser. An Olympus 20x, 1.0 NA water-immersion objective was used to acquire images. The microscope was outfitted with four Hamamatsu GaAsP photomultiplier tubes (PMT) that collected photons at <430 nm, 460 ± 20 nm, 525 ± 25 nm, and 630 ± 37 nm. Using 855 nm excitation, second-harmonic generation (SHG) signal emanating from the sponge was collected with the <430 nm channel, and fluorescence from the surrounding tissue was collected with the 525 nm and 630 nm channels. A sequence of 50 images (512 x 512 pixels; 584 x 584 µm; 13-bit depth) were taken en face at three random locations of the sponge within the wound using 8 kHz resonant galvanometric scanning system that allows for rapid image acquisition. Fourier-based cross-correlation was used to achieve registration of the 50 images prior to frame averaging, which allowed for the removal of any blurred image frames and the correction of motion between frames (https://www.doi.org/10.5281/zenodo.20707807)[18] Fluorescence images were normalized by laser power and PMT gain to µM concentrations of fluorescein to account for differences in PMT voltage and laser power across days. Using MATLAB (https://www.doi.org/10.5281/zenodo.20708392)[19], the SHG signal produced by the collagen in the sponge was used to develop a convex hull that encloses the sponge's boundaries. The mean fluorescence intensity detected at 525 nm emission within the convex hull was obtained for every image to quantify the autofluorescence produced by the tissue surrounding the sponge. Mice were euthanized immediately after two-photon imaging.

At Day 3, label-free multi-photon imaging at the centre of the sponge at Day 3, shows a trend towards an increase in auto-fluorescence in the 525 ± 25nm channel in the combined TrAPs group (Supplementary Figure 20), in response to an 855nm excitation. As autofluorescence have been

associated with cells[20] the auto-fluorescence could perhaps, be due to cellular infiltration or tissue integration. However, we were unable to confirm this hypothesis due to regions lost from the delicate sponge after sectioning and staining (Supplementary Figure 21). The decrease in autofluorescence subsequently at Day 10 in the combined TrAPs group (Supplementary Figure 20), may be due to increased sponge degradation and/or increased light scattering due to a higher density of ECM and cells (Figure 6).

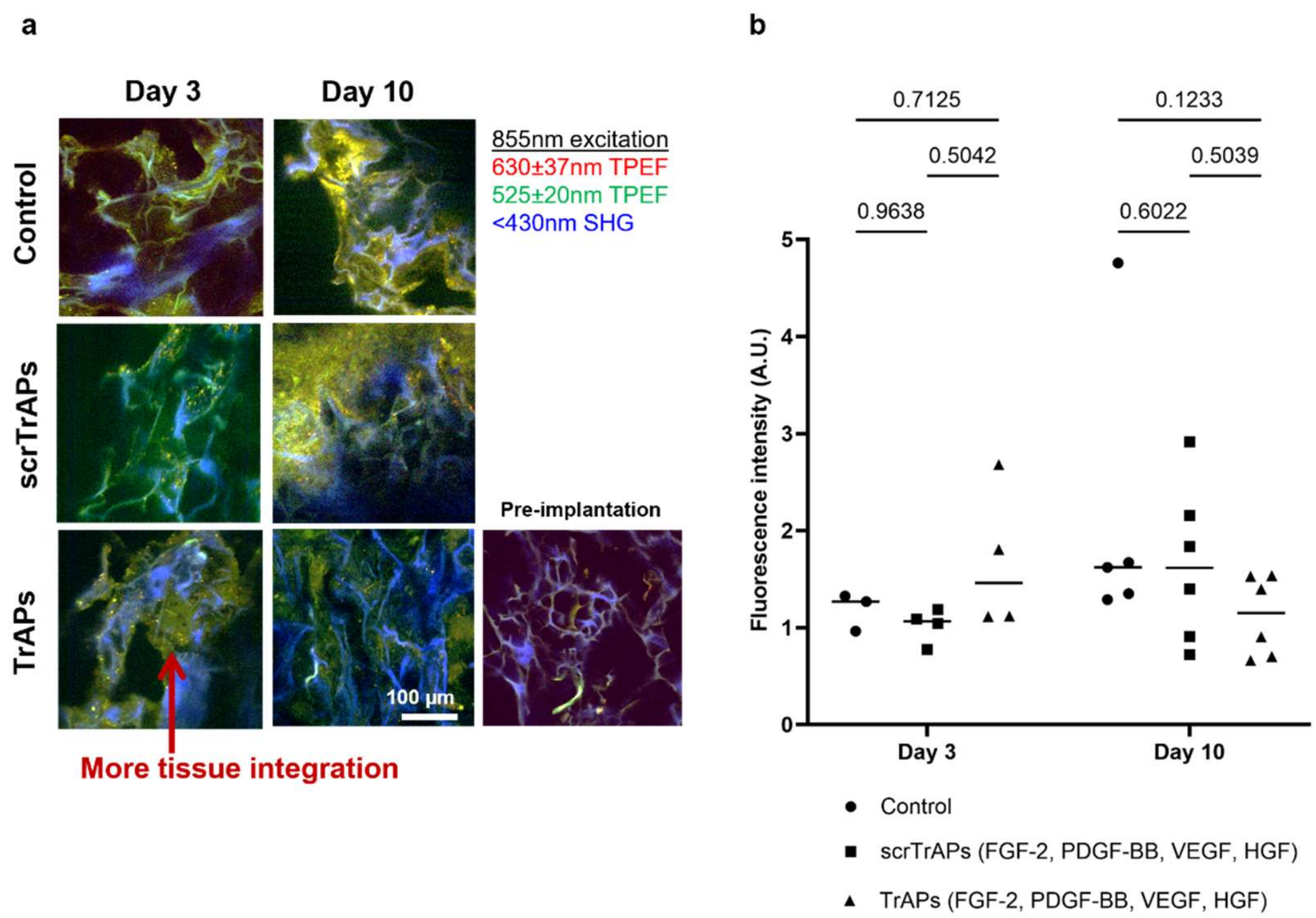

Supplementary Figure 20: In vivo multiphoton imaging of collagen sponge integration with full-thickness mouse skin wound. a, Multiphoton microscopy images of control, combined scrTrAPs-functionalised, and combined TrAPs-functionalised collagen sponges that were implanted on upper dorsum skin wounds of C57Bl/6J mice immediately post-wounding. Images were acquired at 855 nm excitation on day 3 and day 10 post-implantation. Red corresponds to 630 ± 37 nm emission, green to 525 ± 25 nm, and blue to collagen second harmonic generation signal (<430 nm emission) arising from the collagen sponge. A crosslinked collagen sponge was imaged as a reference control for comparison with the autofluorescence signal associated with tissue infiltration during wound healing. **b,** Quantification of the mean fluorescence intensity of the 525 ± 25 nm emission, from a 855 nm excitation, measured at three randomly selected locations within each sponge. Day 3: Control, n = 3 sponges; scrTrAPs, n = 4 sponges; TrAPs n = 4 sponges. Day 10: Control, n = 5 sponges; scrTrAPs, n = 6 wounds; TrAPs, n = 6 sponges. One sponge within a wound was analysed per mouse. Statistical analysis was performed using two-way ANOVA followed by Tukey-Kramer post-hoc test. Data presented as individual data points; horizontal lines indicate median.

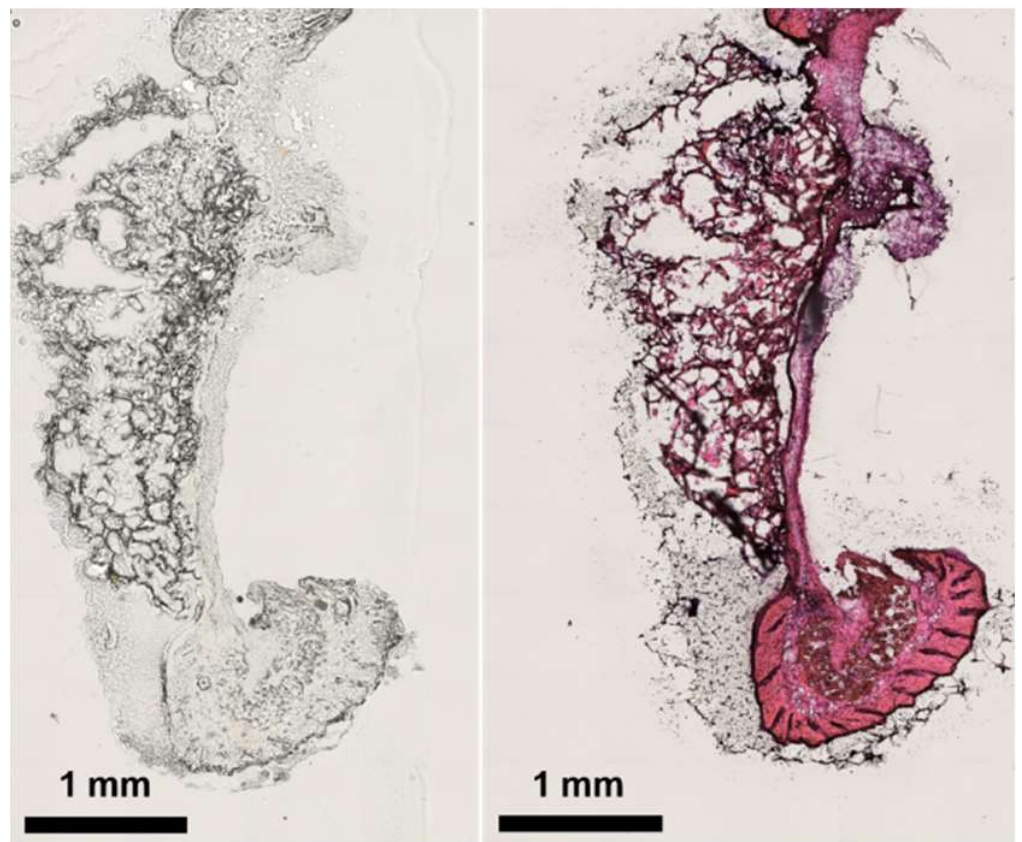

Supplementary Figure 21: Comparison of unstained and H&E-stained skin wound sections. 20 µm sections of the same mouse wound 3 days post-injury.

Calculations and Comparison of Growth Factors Used per Volume

INFUSE Bone graft for spinal fusion (BMP-2)	FGF-2 therapy for leg ulcer, as calculated from literature	TrAPs: VEGF-A for in vivo rat femur	TrAPs: PDGF-BB, FGF-2, HGF and VEGF-A in ex vivo human skin
1.36 $mg/\boldsymbol{cm^3}$	0.54 mg/$\boldsymbol{cm^3}$	0.00051 mg/$\boldsymbol{cm^3}$	0.000003-0.0000116 mg/$\boldsymbol{cm^3}$

**In vivo rat femur study – TrAP-functionalisation**<br>Sponge parameters<br>Diameter: 0.5 cm<br>Height: 0.3 cm<br>Volume:0.0588 cm^3<br>Growth factor added: 0.150 ml of 200 ng/ml VEGF-A per sponge<br>Assuming 100% retention of VEGF-A, the total amount of growth factor per sponge: 30 ng<br>**Growth factor per volume: $\boldsymbol{30/0.0588 = 510\ ng/cm^3}$**
**Ex vivo human skin study – TrAPs-functionalisation**<br>Sponge parameters<br>Diameter: 0.3 cm<br>Height: 0.3 cm<br>Volume:0.0212 cm^3<br>Total amount of HPL loaded onto each sponge at a 33.3% dilution = 0.300ml<br><br>Assuming 100% retention of growth factors in the sponge and using the amount of growth factors present in hPL, determined through ELISA (PDGF-B: 6.90 ng/ml, FGF-2: 0.66 ng/ml, HGF: 2.5 ng/ml, VEGF-A: 0.7ng/ml):<br><br>Total amount of growth factors loaded per 3 mm sponge:<br>**PDGF-B** per sponge volume: (0.3*0.33*6.9) = **0.68 ng**

**FGF-2** in sponge: (0.3*0.33*0.66)= **0.065 ng**<br>**HGF** in sponge: (0.3*0.33*2.5)= **0.25 ng**<br><br>Total amount of growth factor per sponge volume:<br>**PDGF-B** per sponge volume: (0.3*0.33*6.9)/0.0212=**32.2 ng/cm^3**<br>**FGF-2** in sponge: (0.3*0.33*0.66)/0.0212=**3.08 ng/cm^3**<br>**HGF** in sponge: (0.3*0.33*2.5)/0.0212=**11.6 ng/cm^3**<br><br>The wound size in the ex vivo human skin wound was the same size as the sponge – 3mm diameter.
**In vivo mouse skin study – TrAPs-functionalisation**<br>Sponge parameters<br>Diameter: 0.6 cm<br>Height: 0.3 cm<br>Volume:0.084 cm^3<br>Total amount of HPL loaded onto each sponge at a 50% dilution = 0.400 ml<br><br>Assuming 100% retention of growth factors in the sponge and using the amount of growth factors present in hPL, determined through ELISA (PDGF-B: 6.90 ng/ml, FGF-2: 0.66 ng/ml, HGF: 2.5 ng/ml, VEGF-A: 0.7 ng/ml)<br><br>Total amount of growth factors loaded per 0.6 cm diameter sponge:<br>**PDGF-B** per sponge volume: (0.4*0.5*6.9) = **1.38 ng**<br>**FGF-2** in sponge: (0.4*0.5*0.66)= **0.132 ng**<br>**HGF** in sponge: (0.4*0.5*2.5)= **0.5 ng**<br>**VEGF-A** in sponge: (0.4*0.5*0.7)= **0.14 ng**<br><br>Total amount of growth factor per sponge volume:<br>**PDGF-B** per sponge volume: (0.4*0.5*6.9)/0.084 = **16.4 ng/cm^3**<br>**FGF-2** in sponge: (0.4*0.5*0.66)/ 0.084 = **1.57 ng/cm^3**<br>**HGF** in sponge: (0.4*0.5*2.5)/ 0.084 = **5.96 ng/cm^3**<br>**VEGF-A** in sponge: (0.4*0.5*0.7)/ 0.084 = **1.6 ng/cm^3**
**Medtronics INFUSE bone graft** [21]<br><br>Multiple sizes are available. We conduct calculations of two available options, with the amount of growth factor loaded based on the supplier's instructions.<br><br>Device Option 1:<br>Diameter: 1.4 cm<br>Height: 2 cm<br>Estimated volume:3.077 cm^3<br>BMP-2 recommended: 2.8ml*1.5 mg/ml=4.2 mg<br>Assuming 100% retention of BMP-2 in device<br>**Growth factor per volume:** $\frac{4200000}{3.077} = 1,364,965\ ng/cm^3$<br><br>Device Option 2<br>Diameter: 1.8 cm<br>Height: 2.6 cm<br>Volume:6.62 cm^3

BMP-2 recommended: 8 ml*1.5 mg/ml=12 mg<br>Assuming 100% retention of BMP-2 in device<br>**Growth factor per volume:** $\frac{\mathbf{12,000,000}}{\mathbf{6.62}} = \mathbf{1,812,688}\ \boldsymbol{ng/cm^3}$
Data for use of FGF-2 therapy was determined from literature[22,23]<br><br>Growth factor used per day: 30,000 ng<br>Period of use: daily for 3 months<br>Total growth factor used: 30,000 ng*90 days=2,700,000 ng<br><br>Diameter of ulcer was 5-10 cm. As the main goal of the reported study was to achieve re-epithelisation, the wound depth was assumed to be 0.1cm.<br><br>Estimated volume of wound= 5*10*0.1=5 $\boldsymbol{cm^3}$<br><br>Estimated growth factor per volume = $\frac{\mathbf{2,700,000}}{\mathbf{5}} = \mathbf{540,000}\ \boldsymbol{ng/cm^3}$

Supplementary Table 10: Calculations of growth factors used per volume. Note that all TrAP doses are calculated assuming 100% capture efficiency from hPL, a theoretical upper bound that overestimates actual loading. Actual effective doses at the cell surface are lower still, as only a fraction of loaded TrAPs are activated simultaneously by cells. These upper-bound values are presented to demonstrate that even the maximum possible TrAP dose is orders of magnitude below existing clinical value.

Supporting References

1. Gold, Larry. & Janjic, N. High-affinity oligonucleotide ligands to vascular endothelial growth factor (VEGF). *Patent US 7153948 B2* (2003).

2. Green, L. S. *et al.* Inhibitory DNA ligands to platelet-derived growth factor B-Chain. *Biochemistry* **35**, 14413–14424 (1996).

3. Jellinek, D., Lynott, C. K., Rifkin, D. B. & Janjic, N. High-affinity RNA ligands to basic fibroblast growth factor inhibit receptor binding. *Proc. Natl. Acad. Sci. USA* **90**, 11227–11231 (1993).

4. Saito, T. & Tomida, M. Generation of inhibitory DNA aptamers against human hepatocyte growth factor. *DNA Cell Biol.* **24**, 624–633 (2005).

5. Gerhardt, H. *et al.* VEGF guides angiogenic sprouting utilizing endothelial tip cell filopodia. *J. Cell Biol.* **161**, 1163–1177 (2003).

6. Kalukula, Y. *et al.* Unlocking the therapeutic potential of cellular mechanobiology. *Sci. Adv.* **11**, 44 (2025).

7. Geiger, B., Spatz, J. P. & Bershadsky, A. D. Environmental sensing through focal adhesions. *Nat. Rev. Mol. Cell Biol.* **10**, 21–33 (2009).

8. Pieuchot, L. *et al.* Curvotaxis directs cell migration through cell-scale curvature landscapes. *Nat. Commun.* **9**, 3995 (2018).

9. Luciano, M., Tomba, C., Roux, A. & Gabriele, S. How multiscale curvature couples forces to cellular functions. *Nat. Rev. Phys.* **6**, 246–268 (2024).

10. Werner, M., Petersen, A., Kurniawan, N. A. & Bouten, C. V. C. Cell-perceived substrate curvature dynamically coordinates the direction, speed, and persistence of stromal cell migration. *Adv. Biosyst.* **3**, 1900080 (2019).

11. Greenleaf, W. J., Frieda, K. L., Foster, D. A. N., Woodside, M. T. & Block, S. M. Direct observation of hierarchical folding in single riboswitch aptamers. *Science* **319**, 630–633 (2008).

12. Casanova-Morales, N. *et al.* Structural characterization of the saxitoxin-targeting APTSTX1 aptamer using optical tweezers and molecular dynamics simulations. *PLoS One* **14**, e0222468 (2019).

13. Tu, Y., Pal, K., Austin, J. & Wang, X. Filopodial adhesive force in discrete nodes revealed by integrin molecular tension imaging. *Curr. Biol.* **32**, 4386–4396 (2022).

14. Blonski, S. *et al.* Direction of epithelial folding defines impact of mechanical forces on epithelial state. *Dev. Cell* **56**, 3222–3234 (2021).

15. Raucher, D. & Sheetz, M. P. Cell spreading and lamellipodial extension rate is regulated by membrane tension. *J. Cell Biol.* **148**, 127–136 (2000).

16. Stejskalová, A., Oliva, N., England, F. J. & Almquist, B. D. Biologically Inspired, Cell-Selective Release of Aptamer-Trapped Growth Factors by Traction Forces. *Adv. Mater.* **31**, 1806380 (2019).

17. Simmons, C. A., Alsberg, E., Hsiong, S., Kim, W. J. & Mooney, D. J. Dual growth factor delivery and controlled scaffold degradation enhance in vivo bone formation by transplanted bone marrow stromal cells. *Bone* **35**, 562–569 (2004).

18. Quantitative Optical Imaging Lab. kylepquinn/3D-MPM-registration: v1.0 - Initial Release. *Zenodo https://doi.org/10.5281/zenodo.20707807* (2026)

19. Quantitative Optical Imaging Lab. kylepquinn/autofluorescence-infiltration-nature-materials: v1.0 - Initial Release. *https://doi.org/10.5281/zenodo.20708392* (2026)

20. Georgakoudi, I. & Quinn, K. P. Label-Free Optical Metabolic Imaging in Cells and Tissues. *Annu. Rev. Biomed. Eng.* **25**, 413–443 (2023).

21. Wyeth Pharmaceuticals, Inc. Summary of safety and effectiveness data*,* PMA: *P000054* (2002).

22. Kurokawa, I., Hayami, J. & Kita, Y. A therapy-resistant chronic leg ulcer treated successfully with topical basic fibroblast growth factor. *J. Int. Med. Res.* **31**, 149–151 (2003).

23. Abdelhakim, M., Lin, X. & Ogawa, R. The Japanese experience with basic fibroblast growth factor in cutaneous wound management and scar prevention: a systematic review of clinical and biological aspects. *Dermatol. Ther. (Heidelb).* **10**, 569–587 (2020).
