# Supplementary material for: Force-responsive biomaterials drive tissue repair by harnessing endogenous growth factors
Source: Nat Mater. 2026 Jul 27;25(9):1646–57. doi: 10.1038/s41563-026-02682-8 (PMC13518237; doi:10.1038/s41563-026-02682-8)
Supplement: Supplementary file 2 [file 41563_2026_2682_MOESM2_ESM.pdf]

nature portfolio

Corresponding author(s): Benjamin D Almquist

Last updated by author(s): Jun 16, 2026

# Reporting Summary

Nature Portfolio wishes to improve the reproducibility of the work that we publish. This form provides structure for consistency and transparency in reporting. For further information on Nature Portfolio policies, see our Editorial Policies and the Editorial Policy Checklist.

## Statistics

For all statistical analyses, confirm that the following items are present in the figure legend, table legend, main text, or Methods section.

| n/a | Confirmed | |
|---|---|---|
| ☐ | ☒ | The exact sample size (*n*) for each experimental group/condition, given as a discrete number and unit of measurement |
| ☐ | ☒ | A statement on whether measurements were taken from distinct samples or whether the same sample was measured repeatedly |
| ☐ | ☒ | The statistical test(s) used AND whether they are one- or two-sided<br>*Only common tests should be described solely by name; describe more complex techniques in the Methods section.* |
| ☒ | ☐ | A description of all covariates tested |
| ☐ | ☒ | A description of any assumptions or corrections, such as tests of normality and adjustment for multiple comparisons |
| ☐ | ☒ | A full description of the statistical parameters including central tendency (e.g. means) or other basic estimates (e.g. regression coefficient) AND variation (e.g. standard deviation) or associated estimates of uncertainty (e.g. confidence intervals) |
| ☐ | ☒ | For null hypothesis testing, the test statistic (e.g. *F*, *t*, *r*) with confidence intervals, effect sizes, degrees of freedom and *P* value noted<br>*Give P values as exact values whenever suitable.* |
| ☒ | ☐ | For Bayesian analysis, information on the choice of priors and Markov chain Monte Carlo settings |
| ☒ | ☐ | For hierarchical and complex designs, identification of the appropriate level for tests and full reporting of outcomes |
| ☒ | ☐ | Estimates of effect sizes (e.g. Cohen's *d*, Pearson's *r*), indicating how they were calculated |

*Our web collection on statistics for biologists contains articles on many of the points above.*

## Software and code

Policy information about availability of computer code

Data collection

1. Leica Stellaris 5 Light Sheet with LAS X software
2. Leica DMi1 Inverted Microscope
3. Leica SP8 Confocal Microscope
4. Leica  Stellaris 8 FALCON FILM microscope
5. Bruker Ultima Investigator laser scanning microscope
6. Olympus BX51 Microscope
7. Hamamatsu NanoZoomer-SQ Digital Slide Scanner
8. Thermo Scientific Varioskan® Flash
9. Biomomentum Mach-1 Mechanical Testing System (Model: V500css) with a MA410 single axis loadcell
10. Synergy HT Platereader
11. Thermofisher Nanodrop

Data analysis

1. GraphPad PRISM 9, 11
2. MATLAB_R2023b
3. Imaris 9.7.0
4. FIJI/ImageJ2 (1.54)
5. Mach-1 Motion Software v4.4.0.17

Custom MATLAB code that was used to enable image registration for in vivo multiphoton imaging is available via Zenodo at https://www.doi.org/10.5281/zenodo.20707807
Custom MATLAB code for establishing the collagen sponge boundary within the in vivo wounds is available via Zenodo at https://www.doi.org/10.5281/zenodo.20708392

For manuscripts utilizing custom algorithms or software that are central to the research but not yet described in published literature, software must be made available to editors and reviewers. We strongly encourage code deposition in a community repository (e.g. GitHub). See the Nature Portfolio guidelines for submitting code & software for further information.

## Data

Policy information about availability of data

All manuscripts must include a data availability statement. This statement should provide the following information, where applicable:

- Accession codes, unique identifiers, or web links for publicly available datasets
- A description of any restrictions on data availability
- For clinical datasets or third party data, please ensure that the statement adheres to our policy

All data that support the findings of this study are provided with the paper and its Supplementary Information. Source data are provided with the paper.

## Research involving human participants, their data, or biological material

Policy information about studies with human participants or human data. See also policy information about sex, gender (identity/presentation), and sexual orientation and race, ethnicity and racism.

| | |
|---|---|
| Reporting on sex and gender | Human abdominal skin with adipose tissue from female patients aged 25–50 years were obtained from patients undergoing Abdominoplasty procedures at a private clinic, with informed written consent from the patients. |
| Reporting on race, ethnicity, or other socially relevant groupings | Race or ethnicity was not used as recruitment of the ex vivo human skin samples obtained through tummy tuck procedures |
| Population characteristics | Female patients aged 25–50 years were obtained from patients undergoing Abdominoplasty procedures at a private clinic |
| Recruitment | Female patients undergoing Abdominoplasty procedures at a private clinic consented the use of their waste abdominal tissue for research. |
| Ethics oversight | All research protocols were approved by the Imperial College Joint Research Compliance Office. Consent forms used were approved by the Imperial College Research Ethics Committee (REC#21IC6723). |

Note that full information on the approval of the study protocol must also be provided in the manuscript.

# Field-specific reporting

Please select the one below that is the best fit for your research. If you are not sure, read the appropriate sections before making your selection.

☒ Life sciences ☐ Behavioural & social sciences ☐ Ecological, evolutionary & environmental sciences

For a reference copy of the document with all sections, see nature.com/documents/nr-reporting-summary-flat.pdf

# Life sciences study design

All studies must disclose on these points even when the disclosure is negative.

| | |
|---|---|
| Sample size | For the rat femur study, sample sizes were estimated from a power calculation performed at the project licence application stage (assuming a difference of means of 40%, SD of 27%, power of 0.90, $\alpha = 0.05$). For the mouse skin study, sample sizes were set at IACUC-approved minimum levels for a pilot study (IACUC protocol 23034), guided by results from previous wound healing protocols at the same institution. For ex vivo human skin, at least 3 biological donors were used to account for inter-donor variability. |
| Data exclusions | In vitro angiogenic studies: No data from the centre of the hydrogel was excluded.<br>In vitro coverslip experiments: No data were excluded.<br>In vivo rat femur: No data were excluded<br>Ex vivo human skin: No data from non-contaminated samples were excluded. For collection of data from cross-sectional slices, no data was excluded from slices closest to the middle of the wound.<br>In vivo mouse skin: Wounds where the sponge had fallen off during the study were excluded from analysis. |

| | |
|---|---|
| Replication | Multiple in vitro experiments were repeated and reported to demonstrate the reproducibility of TrAPs-enabled delivery of growth factors across cell types (e.g. human dermal fibroblasts and HMEC-1), types of growth factors (e.g. VEGF, HGF, PDGF-BB and FGF-2) and species (e.g. in vivo rat femur, in vivo mouse skin and ex vivo human skin). When primary cells and ex vivo human skin were used, experiments were repeated with cells or/and skin isolated from additional donors to account for potential donor-bias observations. |
| Randomization | For in vivo studies, animals were randomly assigned into groups prior to the start of the experiment, with equal number of female and male mouse where applicable. For preparation of TrAPs-functionalised collagen scaffolds, coverslips and hydrogels, materials were prepared in the same batch, and randomly assigned into groups only at the step were TrAPs or scrTrAPs were conjugated onto the scaffold. Cells and skin samples used in different treatments groups were likewise processed in the same flask/from the same skin, up till the step where different TrAP-scaffolds or scr-TrAPs were randomly assigned to a skin sample or well-plate/coverslip. |
| Blinding | For the in vivo rat femur study, outcome assessment and data analysis were performed blinded to group allocation, in accordance with refinement provisions of the project licence. For the in vivo mouse skin study and all in vitro and ex vivo experiments, investigators were not blinded during data collection and assessment; data were collected and assessed uniformly across groups using pre-determined, objective quantification methods. |

# Reporting for specific materials, systems and methods

We require information from authors about some types of materials, experimental systems and methods used in many studies. Here, indicate whether each material, system or method listed is relevant to your study. If you are not sure if a list item applies to your research, read the appropriate section before selecting a response.

## Materials & experimental systems

| n/a | Involved in the study |
|---|---|
| ☐ | ☒ Antibodies |
| ☐ | ☒ Eukaryotic cell lines |
| ☒ | ☐ Palaeontology and archaeology |
| ☐ | ☒ Animals and other organisms |
| ☒ | ☐ Clinical data |
| ☒ | ☐ Dual use research of concern |
| ☒ | ☐ Plants |

## Methods

| n/a | Involved in the study |
|---|---|
| ☒ | ☐ ChIP-seq |
| ☒ | ☐ Flow cytometry |
| ☒ | ☐ MRI-based neuroimaging |

## Antibodies

| | |
|---|---|
| Antibodies used | From Abcam: Anti-CD-31 (ab182981), Anti-a-SMA (ab7817), Goat anti-rabbit (ab97049), goat anti-mouse (ab6788)<br>From Thermo Fisher: Anti-vWF (MAS-14029), Goat anti-Chicken Alexa 488 (A-11039)<br>From Biolegend: anti-Keratin 14 (SIG-3476-100) |
| Validation | All antibodies were verified by the vendor, with validation data available on the manufacturer's website (see below). These antibodies have also been used and cited by published papers, as listed on the manufacturer's website.<br><br>1.CD-31 (ab182981): "Specificity and sensitivity confirmed in IHC with multi-tissue microarray (TMA) validation." https://www.abcam.com/en-us/products/primary-antibodies/cd31-antibody-epr17259-ab182981<br><br>2. Anti-a-SMA (ab7817): Validated by Abcam for IHC use. https://www.abcam.com/en-us/products/primary-antibodies/alpha-smooth-muscle-actin-antibody-1a4-ab7817<br><br>3. Goat anti-rabbit (ab97049): validated by Abcam for IHC use. https://www.abcam.com/en-us/products/secondary-antibodies/goat-rabbit-igg-h-l-biotin-ab97049<br><br>4. Goat anti-mouse (ab6788): validated by Abcam for IHC use. https://www.abcam.com/en-us/products/secondary-antibodies/goat-mouse-igg-h-l-biotin-ab6788<br><br>5.Anti-vWF (MA5-14029): validated by Thermo Fisher for IHC use. https://www.thermofisher.com/antibody/product/VWF-Antibody-clone-F8-86-Monoclonal/MA5-14029<br><br>6. anti-Keratin 14 (SIG-3476-100): validated by Biolegend for IHC use. https://www.biolegend.com/en-us/search-results/purified-anti-keratin-14-antibody-13379<br><br>7. Goat anti-Chicken Alexa 488 (A-11039): validated by Thermo Fisher for IHC use: https://www.thermofisher.com/antibody/product/Goat-anti-Chicken-IgY-H-L-Secondary-Antibody-Polyclonal/A-11039 |

## Eukaryotic cell lines

Policy information about cell lines and Sex and Gender in Research

| | |
|---|---|
| Cell line source(s) | Human microvascular endothelial cells (HMEC-1, obtained from supplier ATCC, CRL-3243 ) |

| | |
|---|---|
| Cell line source(s) | Primary human skin dermal fibroblasts (HDFs) derived from tummy tucks of healthy female individuals were gifted from Dr Claire Higgins in the Department of Bioengineering at Imperial College London |
| Authentication | A-11039<br><br>For HMEC-1, STR profiling was performed by supplier as stated: "Amelogenin: X,Y<br>CSF1PO: 10, 12<br>D13S317: 11, 12<br>D16S539: 11, 12<br>D5S818: 12<br>D7S820: 8, 10<br>THO1: 7<br>TPOX: 8, 11<br>vWA: 15, 17"<br><br>HDFs were not authenticated |
| Mycoplasma contamination | Not detected during culture facility screening |
| Commonly misidentified lines (See ICLAC register) | N/A |

## Animals and other research organisms

Policy information about studies involving animals; ARRIVE guidelines recommended for reporting animal research, and Sex and Gender in Research

| | |
|---|---|
| Laboratory animals | Female Wistar rats were purchased at age 5 to 9 weeks from Charles River Laboratories and used one week after allowing to acclimatise.<br><br>C57BL/6J mice purchased from Jackson Laboratory (age: 12 weeks; 50/50 sex split) |
| Wild animals | Did not involve wild animals |
| Reporting on sex | Female Wistar rats were used for the in vivo rat femur study.<br>Equal number of female and male mice were used for the in vivo mouse skin study (except for a female mouse in the control group that passed upon arrival, before the commencement of the experiment). |
| Field-collected samples | Did not involve animals collected from the field |
| Ethics oversight | The in vivo rat femur study was conducted in compliance with the Home Office license ([Scientific Procedures] Act 1986 (PPL PD51CDDA3).<br><br>The in vivo mouse skin study was approved and performed according to the University of Arkansas IACUC (Protocol #23034) |

Note that full information on the approval of the study protocol must also be provided in the manuscript.

## Plants

| | |
|---|---|
| Seed stocks | N/A |
| Novel plant genotypes | N/A |
| Authentication | N/A |
